# Enhanced Multi-Strategy Slime Mould Algorithm for Global Optimization Problems

**DOI:** 10.3390/biomimetics9080500

**Published:** 2024-08-17

**Authors:** Yuncheng Dong, Ruichen Tang, Xinyu Cai

**Affiliations:** 1 School of Highway and Construction Engineering, Yunnan Communications Vocational and Technical College, Kunming 650500, China; ycdong@ynjtc.edu.cn; 2 College of Electrical Engineering and Information, Northeast Agricultural University, Harbin 150030, China; a12220362@neau.edu.cn; 3 College of Business, Jiaxing University, Jiaxing 314001, China

**Keywords:** Slime Mould Algorithm, non-monopoly search, restart mechanism, numerical optimization, CEC 2017 test suite, CEC 2022 test suite

## Abstract

In order to further improve performance of the Slime Mould Algorithm, the Enhanced Multi-Strategy Slime Mould Algorithm (EMSMA) is proposed in this paper. There are three main modifications to SMA. Firstly, a leader covariance learning strategy is proposed to replace the anisotropic search operator in SMA to ensure that the agents can evolve in a better direction during the optimization process. Secondly, the best agent is further modified with an improved non-monopoly search mechanism to boost the algorithm’s exploitation and exploration capabilities. Finally, a random differential restart mechanism is developed to assist SMA in escaping from local optimality and increasing population diversity when it is stalled. The impacts of three strategies are discussed, and the performance of EMSMA is evaluated on the CEC2017 suite and CEC2022 test suite. The numerical and statistical results show that EMSMA has excellent performance on both test suites and is superior to the SMA variants such as DTSMA, ISMA, AOSMA, LSMA, ESMA, and MSMA in terms of convergence accuracy, convergence speed, and stability.

## 1. Introduction

**Figure 1:**
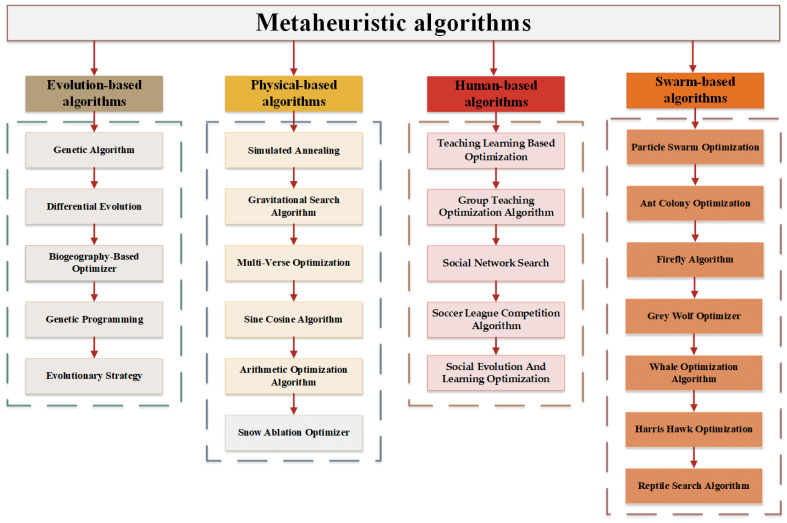
Summary of some metaheuristic algorithms.

In the present era of rapidly changing technology, numerous complex engineering optimization problems have emerged in many fields, such as social life and scientific research [1]. The emergence of these problems has undoubtedly increased the importance of optimization algorithms and artificial intelligence technology [2]. The central goal of optimization is to find an optimal solution to the objective function while adhering to a set of constraints [3]. Previously, the optimal solution has often been searched for by relying on information about the gradient of the function involved [4]. Although this approach has shown its effectiveness in some specific cases, as a variety of real-world optimization problems become nonconvex, discontinuous, nonlinear, and multimodal, traditional gradient-based optimization methods are far from being able to meet the growing practical demands [5,6]. In these cases, it may not be possible to achieve satisfactory results using traditional methods, and therefore, it becomes particularly necessary to discover alternative methods. Faced with the challenge of traditional optimization methods that are difficult to cope with due to the enormity of the problem size or the complexity of the solution space, metaheuristic algorithms have emerged as an effective alternative with their unique search strategies and high adaptability [7]. Metaheuristic algorithms are algorithms that mimic natural or biological evolutionary mechanisms, and they are gaining popularity among researchers. A significant advantage of these algorithms is that they do not rely on explicit problem formulations or mathematical equations and can cope with challenging problems even in the absence of gradient information, offering the possibility of finding near-optimal solutions [8]. In addition, metaheuristics are known for their simplicity, adaptability, lack of need for a derivation framework, and ability to overcome local optima [9]. These properties empower them to solve complex mathematical problems and provide valuable approximate solutions even when the optimal solution is difficult to achieve. In the last few years, there has been an explosion in the research and application of metaheuristic algorithms. In addition to classical numerical optimization, metaheuristic algorithms have demonstrated their effectiveness in a wide range of optimization tasks including, but not limited to, feature selection [10,11,12], traveling sales problems [13,14], image segmentation problems [15,16,17,18], wireless sensor coverage problems [19,20,21], and mission planning [22,23,24]. Depending on their source of inspiration, these algorithms can be broadly categorized into four main groups: evolution-based, physical-based, human-based, and swarm-based. Each category provides a unique perspective and approach to solving optimization problems. The different kinds of algorithms that will be presented below are given in Figure 1.

Evolution-based algorithms are a class of metaheuristics inspired by evolutionary mechanisms. These algorithms are based on Darwin’s theory of natural selection, which leads to a gradual solution through superiority and inferiority. Genetic algorithms (GA) mimic the natural evolutionary process through mutation, recombination and natural selection [25]. In addition, the differential evolution (DE) proposed by Price et al. optimizes the solution by simulating natural selection and genetic mechanisms using the differences between individuals in a population [26]. These two algorithms are the most well-known evolutionary algorithms. Other evolution-based algorithms include Biogeography-Based Optimizer (BBO) [27], Genetic Programming (GP) [28], Evolutionary Strategies (ES) [29], etc.

Physics-based algorithms draw from the laws of physics or mathematical methods to mimic natural physical phenomena. These algorithms use physical concepts such as energy, force, and temperature to guide the search process. Simulated annealing (SA) [30] simulates the slow cooling process of the metal to minimize defects. The Gravity Search Algorithm (GSA) is inspired by the law of gravity in physics [31]. Other popular physics-based algorithms are Multi-Verse Optimization (MVO) [32], the Sine Cosine Algorithm (SCA) [33], and the Arithmetic Optimization Algorithm (AOA) [34].

Human-based algorithms are usually inspired by human social behavior and interaction styles. These algorithms mimic the characteristics of human social and personal behavior and aim to explore and develop the problem space efficiently through this approach. Brainstorming optimization (BSO) is inspired by the process of generating and developing ideas in collective discussions [35]. Rao et al. proposed teaching–learning-based Optimization (TLBO) by simulating the teaching and learning process between teachers and students [36]. Other human-inspired algorithms include the Group Teaching Optimization Algorithm (GTOA) [37], Social Network Search (SNS) [38], Soccer League Competition Algorithm (SLC) [39], and Social Evolution and Learning Optimization (SELO) [40], among others.

Swarm-based algorithms are inspired by the group behavior of organisms such as ants, bees, birds, and fish. The field of swarm-based algorithms is very active, and many experts and scholars have proposed various algorithms inspired by the behavior of living creatures. Particle Swarm Optimization (PSO) is one of the most classical algorithms inspired by the behavior of flocks of birds [41]. Ant Colony Optimization (ACO) is inspired by the foraging behavior of ants [42]. Other swarm-based algorithms include Firefly Algorithm (FA) [43], Grey Wolf Optimizer (GWO) [44], Whale Optimization Algorithm (WOA) [45], Harris Hawk Optimization (HHO) [46], Reptile Search Algorithm (RSA) [47], Dwarf Mongoose Optimization (DMO) [48], Tuna Swarm Optimization (TSO) [49], Remora Optimization Algorithm (ROA) [50], and Crayfish Optimization Algorithm (COA) [51]. In conclusion, swarm-based algorithms provide an effective method for finding optimal or near-optimal solutions to complex optimization problems, and their rapid development has had a profound impact on modern human life.

The basic framework of metaheuristic algorithms consists of two phases: the exploration phase and the development phase [46]. Metaheuristic algorithms need to strike a balance between these two phases so as to ensure optimal results in one or more specific applications. In the exploration phase, the algorithm focuses primarily on searching the solution space extensively to discover potential solutions. In the exploitation phase, the algorithm concentrates on further improving and optimizing the discovered solutions to enhance their quality and performance. Although there is a wide variety of optimization algorithms, according to the “no free lunch” theorem [52], it is almost impossible for one optimization algorithm to solve all optimization problems well. Different algorithms have different effects in solving different problems. Therefore, new optimization methods or improved versions of existing techniques must be proposed to solve tasks in different domains. Recently, inspired by the central concept of slime mold model, Li et al. proposed a new technique to mimic the behavior of slime mold swarms, namely the Slime Mould Algorithm (SMA) [53]. The algorithm was evaluated on several benchmark functions and compared with other well-known metaheuristics on four engineering design algorithms, thus affirming its superiority and efficiency. Due to its simple structure and clear principles, SMA has attracted much attention since its introduction in 2020 and has been used to solve a large number of real-world optimization problems [54,55,56,57].

Although SMA has been successfully applied in several fields, SMA still has some drawbacks, such as imbalance between exploration and exploitation, and the tendency to fall into local optimization at the late stage of iteration. Considering these limitations, the Enhanced Multi-Strategy Slime Mould Algorithm (EMSMA) is proposed in this study. The main contributions of this research are summarized as follows:

1. The leader covariance learning strategy is used as an alternative to the anisotropic search operator to utilize the information of the dominant population to make the search toward more promising regions, thus enhancing the quality of candidate solutions and improving the search performance of SMA.
2. An improved non-monopoly search mechanism is designed to execute further search on the optimal agent so as to refine the quality of the optimal individual and help the SMA to avoid local optimal traps.
3. A random differential restart mechanism is developed. Perturbation is applied to the agents when the search becomes stagnant, thus enhancing the population diversity and avoiding premature convergence of SMA.
4. The effects of parameter selection and strategy on SMA are discussed on the CEC2017 and CEC2022 test suites, and the performance of EMSMA is evaluated by comparison with several SMA variants. The superior performance of EMSMA is demonstrated using mean, standard deviation, convergence curves, box plots, the Wilcoxon rank sum test, and the Friedman test.

The rest of the paper is organized as follows: Section 2 introduces the basic principles of SMA. Section 3 presents the details of EMSMA, followed by time complexity analysis. Section 4 is the experimental part, where the performance of the proposed EMSMA is evaluated by means of the CEC2017 and CEC2022 test suites and compared to other SMA variants. We summarize the study and provide an outlook for the future in Section 5.

## 2. Overview of Slime Mould Algorithm

In the initialization phase, the initial candidate solutions are randomly generated throughout the search region. The initial candidate solution can be calculated as follows.

(1) $$X_{i}^{Ini}=rand\times (ub-lb)+lb,i=1,2,\ldots ,N$$

where $X_{i}^{Ini}$ indicates the position of the *i*th initial individual and ub and lb are the upper and lower bounds of the optimization problem. N represents the number of agents. The solution matrix *X* is described as follows.

(2) $$X=\left(\begin{array}{cccccc} x_{1,1} & \ldots & x_{1,j} & x_{1,j+1} & \ldots & x_{1,D}\\ \vdots & \ddots & \vdots & \vdots & \ddots & \vdots \\ x_{i,1} & \cdots & x_{i,j} & x_{i,j+1} & \cdots & x_{i,D}\\ x_{i+1,1} & \ldots & x_{i+1,j} & \ddots & \ldots & x_{i+1,D}\\ \vdots & \ddots & \vdots & \vdots & \ddots & \vdots \\ x_{N,1} & \cdots & x_{N,j} & x_{N,j+1} & \cdots & x_{N,D} \end{array}\right)$$

where *D* denotes the dimension of the optimization problem.

In this section the mathematical model of SMA will be presented. SMA is able to approach the food according to the level of odor concentration and mainly simulates the different morphological variations of slime moulds. These morphologies are calculated by the following three equations.

(3) $$X_{i}^{t+1}=rand\times (ub-lb)+lb,rand<z$$

(4) $$X_{i}^{t+1}=X_{best}+vb\times (W\times X_{a}^{t}-X_{b}^{t}),rand<p$$

(5) $$X_{i}^{t+1}=vc\times X_{i}^{t},rand\geq p$$

where t denotes the current number of iterations. rand represents a random number randomly generated between 0 and 1. z is a constant that takes the value of 0.03 in SMA. $X_{best}$ denotes the global best agent. vb are random numbers in the range $\left[-a,a\right]$. W denotes the fitness weight of the candidate agents, which is obtained from Equation (8). $X_{a}^{t}$ and $X_{b}^{t}$ are two different agents randomly selected from the population. vc is a number that decreases linearly from 1 to 0.

(6) $$p=\tanh\left|F_{i}-DF\right|,i=1,2,3,\ldots ,N$$

(7) a=arctanh(−(t/T)+1)

(8) $$W(smellindex(i))=\left\{\begin{array}{l} 1+rand\times \log(\frac{bF-F_{i}}{bF-\omega F}),condition\\ 1-rand\times \log(\frac{bF-F_{i}}{bF-wF}),others \end{array}\right.$$

(9) smellindex=sort(F)

where $F_{i}$ denotes the fitness of agents. DF is the current best fitness. T stands for the maximum number of iterations. condition denotes the top half of the agents with better fitness. bF and wF denote the best fitness and the worst fitness of current population, respectively.

## 3. Proposed EMSMA

The modifications to SMA mainly contain three parts. Firstly, the leader covariance learning strategy is adopted in the anisotropy search stage to fully utilize effective information of the excellent population and correctly guide the direction of population evolution. Then, an improved non-monopoly search mechanism is adopted for the best agent to further enhance the quality of best agent. Finally, the random differential restart mechanism is employed to help SMA jump out of the local optimum and enhance the population diversity when the agents become stagnant. The details are shown below.

### 3.1. Leader Covariance Learning Strategy (LCLS)

In the basic SMA, when rand<z, the population can explore any region of the problem space through a stochastic search method (Equation (1)), but this equation is rarely used. In addition, Equation (1) has very powerful exploration capability, but the search efficiency is low, so it is necessary to use other methods instead. This paper proposes a leader covariance learning strategy (LCLS) based on Gaussian distribution estimation methods [58]. The Gaussian estimated distribution method is an algorithm that relies on sampling probability distributions, where the Gaussian distribution model is more effective in guiding the algorithm to explore more promising regions. In addition, to further decentralize the search, the strategy is modified with regards to the location of the best agent. The formulas for the leader covariance learning strategy are expressed as follows:

(10) $$X_{i}^{t+1}=\left(X_{leader}+X_{mean}+X_{i}^{t}\right)/3+y,y\sim N\left(0,C_{i}\right)$$

(11) $$X_{leader}\in \left\{X_{best},X_{\mathrm{second}},X_{third}\right\}$$

(12) $$C_{i}=\frac{1}{\left|S\right|}\sum_{i=1}^{\left|S\right|}\left(S_{i}-X_{mean}\right)\times {\left(S_{i}-X_{mean}\right)}^{T}$$

(13) $$X_{mean}=\sum_{i=1}^{\left|S\right|}\omega_{i}\times X_{i}$$

(14) $$\omega_{i}=\ln\left(\left|S\right|+1\right)/\left(\sum_{i=1}^{\left|S\right|}\left(\ln\left(\left|S\right|+1\right)-\ln\left(i\right)\right)\right)$$

where S is the set consisting of the better performing half of the individuals in the population. $\left|S\right|$ denotes the cardinality of the set S. $C_{i}$ denotes the covariance of each agent. $X_{leader}$ is an individual randomly selected from the best three agents. $\omega_{i}$ is the weight of each agent. In SMA, a discussion on the value of z is necessary after LCLS replaces Equation (1). The magnitude of the z value determines how often LCLS is used. In this paper, the value of z is set to 0.3 after experiments. Details are listed in the experimental section.

### 3.2. Improved Non-Monopoly Search Mechanism (INSM)

The quality of the optimal agent affects the search direction of the whole population. When the optimal agent search becomes stagnant, there is a possibility that the whole population cannot carry out the search, so it is necessary to make further amendments to the optimal agent. To enhance the quality of optimal individual, this paper proposes an improved non-monopoly search mechanism (INSM). The non-monopoly search mechanism is a novel local search method [59] proposed by Abualigah in 2024. The mechanism controls exploitation and exploration according to the iterative process and has the ability to escape from local optimality by relying on stochastic operators. Specifically, the optimal individual will be explored in the first half of iterations as in Equation (14) and exploited in the second half of iterations as in Equation (15).

(15) $$X_{best}(j)=rand\times X_{best}(RP)$$

(16) $$X_{best}(j)=X_{best}(j)-\left(X_{best}(RP)\times rand\right)\times eps-\left(X_{best}(j)-1\right)$$

where $X_{best}(RP)$ is a randomly selected dimension from the best agent. The marine predator algorithm utilizes Levy flight and Brownian motion to construct foraging strategies with better results [60]. Among them, Levy flights are mainly small-scale movements and occasional large step-length movements. Brownian motion, on the other hand, can be used to track and explore various areas of the domain by virtue of uniform and controllable step lengths. Considering the characteristics of the two approaches, this paper combines Brownian motion into Equation (15) and Levy flight into Equation (16) for a more comprehensive exploration and more refined exploitation of the problem space. The improved non-monopolized search mechanism is represented as follows:

(17) $$X_{best}(j)=RB\times X_{best}(RP)$$

(18) $$X_{best}(j)=X_{best}(j)-\left(X_{best}(SRP)\times rand\right)\times eps-RL\times \left(X_{best}(j)-1\right)$$

where RB and RL are random numbers obeying Brownian and Levy distributions, respectively. eps is a small value and takes the value $2.2204^{-16}$.

### 3.3. Random Differential Restart Mechanism (RDRM)

**Figure 2:**
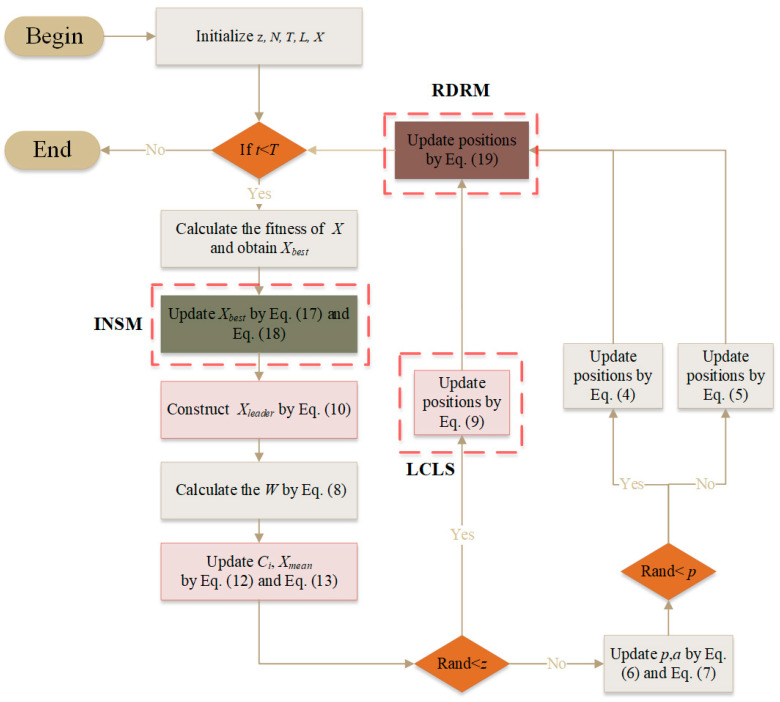
The flow chart of the EMSMA.

To facilitate the algorithm to jump out of the local optimum, this paper develops a random differential restart mechanism. Specifically, RDRM is applied to an individual when the stagnation count L of an agent accumulates to the restart threshold. The stagnation count L is calculated by adding 1 to the count L when the children of this agent are inferior to the parent and vice versa with no change. When RDRM is used, if the child individual is superior to the parent, the count L is reset to 0 and vice versa plus 1. The RDRM is represented as follows:

(19) $$X_{i}^{t+1}=\left\{\begin{array}{l} X_{i}^{t}+{\left(1-\frac{t}{T}\right)}^{\frac{2t}{T}}\times \left(lb+rand\times \left(ub-lb\right)\right)\times U,rand\leq 0.2\\ X_{i}^{t}+\left(0.2\times \left(1-rand\right)\right)+rand\times \left(X_{c}^{t}-X_{d}^{t}\right),rand>0.2 \end{array}\right.$$

(20) $$U_{i,j}=\left\{\begin{array}{c} 1,rand<0.2\\ 0,rand\geq 0.2 \end{array}\right.,i=1,2,\ldots ,N;j=1,2,\ldots ,D$$

where $X_{c}^{t}$ and $X_{d}^{t}$ are two different candidates randomly selected from the population. The pseudo code and flowchart of the EMSMA are shown in Algorithm 1 and Figure 2.
**Algorithm 1** Pseudo code of the EMSMABegin: //Initialization   Initialize z, *N*, *T*, *L*;  Initialize the the positions of agent *X* //Main loop  While (*t* < *T*) do   Calculate the fitness of *X* and obtain $X_{best}$
   Construct $X_{leader}$ by Equation (10)   Calculate the *W* by Equation (8)   Update $C_{i}$, $X_{mean}$ by Equations (12) and (13)   For *i* = 1: *N* do    Update *p,a* by Equations (4) and (7)    If rand < z then     Update positions by Equation (9) //Leader covariance learning strategy    else     Update positions by Equations (4) and (5)    End if   End For   Update $X_{best}$ by Equations (17) and (18) //Improved non-monopoly search mechanism   For *i* = 1: *N* do    Update positions by Equation (19) //Random differential restart mechanism   End for   *t* = *t* + 1  End WhileReturn: the best fitness and $X_{best}$$X_{best}$


### 3.4. Time Complexity Analysis

Time complexity analysis is also an important part of evaluating the performance of an algorithm. The time complexity depends on the following three parameters: the number of populations N, the problem dimension D, and the number of iterations T. The specific analysis is described below:

For SMA, the initialization phase time is $O\left(N\times D\right)$. The time complexity of position updating is $O\left(T\times N\times D\right)$. In general, the total time complexity of SMA is $O\left(SMA\right)=O\left(N\times D\right)+O\left(T\times N\times D\right)=O\left(\left(T+1\right)\times N\times D\right)=O\left(T\times N\times D\right)$.

For EMSMA, the initialization phase time is $O\left(N\times D\right)$. The time complexity of the improved non-monopoly search mechanism is $O\left(T\times D\right)$.The time complexity of the leader covariance learning strategy is $O\left(T\times D\times 0.3N\right)$. The time complexity of position updating using Equations (2) and (3) is $O\left(T\times D\times 0.7N\right)$. For the random differential restart mechanism, we assume each agent is reinitialized by the restart mechanism every *Limit* time, so the time complexity is $O\left(T/Limit\times D\times N\right)$. Therefore, the complexity of the EMSMA can be expressed as below:

$$\begin{array}{l} O\left(EMSMA\right)=O\left(N\times D\right)+O\left(T\times D\times 0.3N\right)+O\left(T\times D\times 0.7N\right)+O\left(T\times D\right)+O\left(T/Limit\times D\times N\right)\\ =O\left(N\times D+N\times D\times T+D\times T+T/Limit\times D\times N\right)\\ =O\left(T\times N\times D\left(1+1/Limit\right)\right) \end{array}$$

To summarize, the time complexity of EMSMA is higher than SMA, but the performance of EMSMA is significantly improved compared to SMA, so this is acceptable.

## 4. Experimental Results and Discussion

In this section, comparative experiments are performed on the CEC2017 and CEC2022 test suites, where the competitors contain several SMA variants. Through these experiments, the performance of EMSMA is evaluated from different perspectives, including convergence accuracy analysis, stability analysis, Friedman test analysis, and Wilcoxon rank sum test analysis. In addition, this section discusses the effects of z-values and different improvement strategies on EMSMA.

### 4.1. Experimental Environment and Parameter Settings

All the experiments in this paper were performed on MATLAB R2020a and Windows 10 operating system. The hardware is as follows: AMD R9 7950X CPU (4.5 Ghz) and 32 GB RAM. To evaluate the performance of EMSMA, six SMA variants participated in the test as competitors. The performance of the algorithms was evaluated using the mean (Mean), standard deviation (STD), and minimum (Best), and Friedman scores were calculated for each algorithm. The Wilcoxon rank sum test was then used to assess whether there were significant differences between the EMSMA algorithm and the competitors. For fair comparison and to reduce randomness, all algorithms use the same common parameters, and each algorithm is run independently 51 times with a population size of 50 and an iteration number of 1000. The parameter settings for the competitors are referenced from the original literature and are shown in Table 1. In addition, Table 1 presents the improvement strategies for each SMA variant.

### 4.2. Benchmark Test Functions

The CEC2017 and CEC2022 test suites were used to evaluate the performance of EMSMA and competitors. The CEC2017 test suite has 29 functions, including 2 unimodal, 7 multimodal, 10 hybrid, and 10 composite functions [66]. The CEC2022 test suite has 12 functions, including 1 unimodal, 3 basic, 3 hybrid, and 4 composite functions [67]. The details of the two test suites are presented in Table 2 and Table 3.

### 4.3. Sensitivity of EMSMA to Parameter z

**Figure 3:**
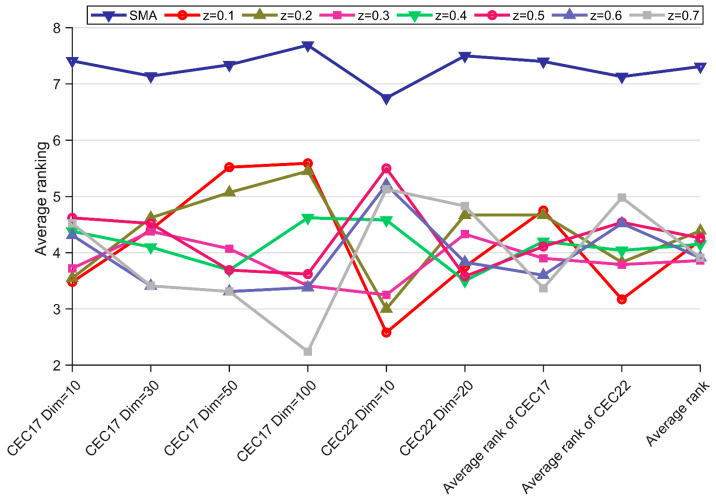
Rankings of EMSMA with different z settings obtained from the Friedman test.

In order to fully utilize the leader covariance learning strategy, the parameter z in EMSMA needs to be discussed for optimal settings. In this subsection, the z values are set to 0.1, 0.2, 0.3, 0.4, 0.5, 0.6 and 0.7. To demonstrate the effect of the best parameter setting, we evaluate the performance of the EMSMA under different z values by using the test functions under different dimensions of the CEC2017 and CEC2022 test suites. Table 4 presents the Friedman scores and *p*-values of EMSMA at different z-values, and Figure 3 visualizes the results. The *p*-values in Table 4 are all less than 0.05, indicating that there is a significant difference between EMSMA and SMA regardless of the parameter z setting. As can be seen in Figure 3, EMSMA has an overall mean score of 3.86 when z is set to 0.3. Although EMSMA has the highest score in the CEC 2017 (CEC 2022) test suite when z = 0.6 (0.1), EMSMA also ranks in the top three for the separate mean scores of the two test suites and first for the overall mean scores when z = 0.3. Therefore, in this paper, the value of parameter z is set to 0.3 to fully demonstrate the performance of EMSMA.

### 4.4. Strategies Effectiveness Analysis

**Figure 4:**
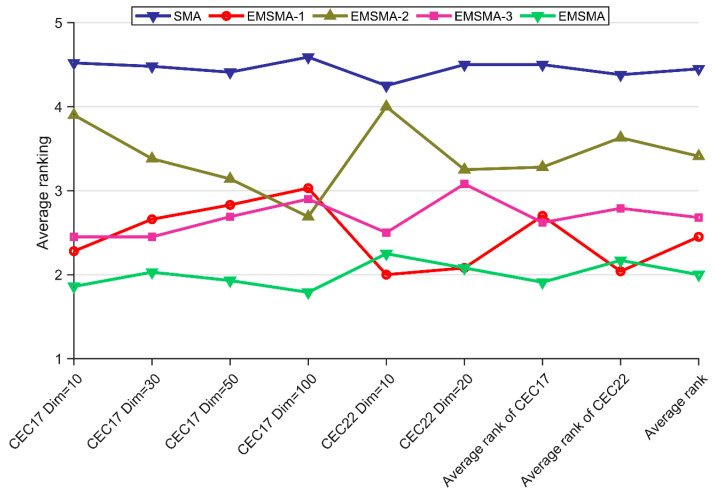
Rankings of EMSMA and its variants.

Similarly, we evaluate the effectiveness of strategies proposed in this paper in CEC2017 and CEC2022 test suites. In this subsection, three EMSMA variants are set up, namely EMSMA-1, EMSMA-2, and EMSMA-3. The three EMSMA variants are obtained by EMSMA with LCLS, INSM and RDRM removed, respectively. Specific numerical results are shown in Table A1, Table A2, Table A3, Table A4, Table A5 and Table A6 in Appendix A. The results of the Friedman test for EMSMA and the three EMSMA-derived algorithms are given in Table 5. As can be seen from Table 5, the *p*-value is less than 0.05 in all cases, which indicates that there is a significant difference between the algorithms experimented in this section. Figure 4 visualizes the ranking of EMSMA and its derived algorithms.

As can be seen in Figure 4, EMSMA has the best ranking in the average rank of total column, which means that the overall performance of EMSMA is better than the comparison algorithms. It is noteworthy that EMSMA and its variants are ranked higher than SMA in all cases, which reflects the significant effect of the improvement strategy in this paper. The specific analysis is as follows. EMSMA-2 with RDRM removed ranked last among the three variants, suggesting that RDRM is effective in improving the performance of SMA. EMSMA-1 and EMSMA-3 ranked similarly, indicating that the two strategies, LCLS and INSM, have similar levels of effectiveness for SMA. Although EMSMA is worse than EMSMA-1 in CEC2022 Dim = 10, the overall performance is still the best. In summary, through the experiments in this subsection, it can be concluded that LCLS, INSM, and RDRM are all effective in boosting SMA.

### 4.5. Comparison with SMA Variants

**Figure 5:**
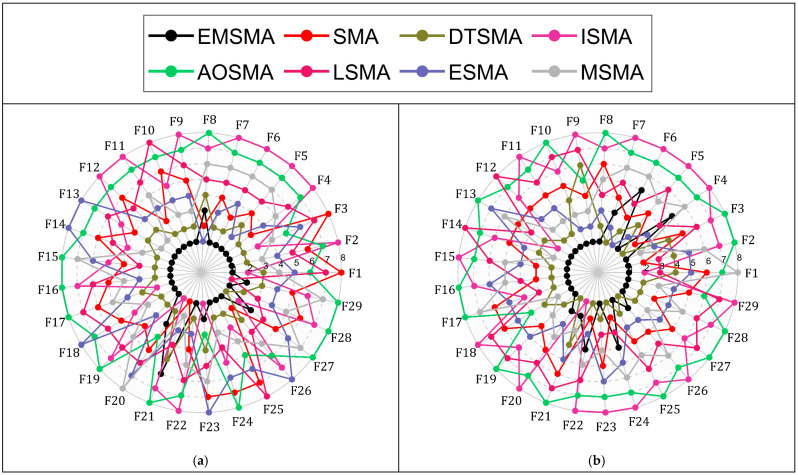

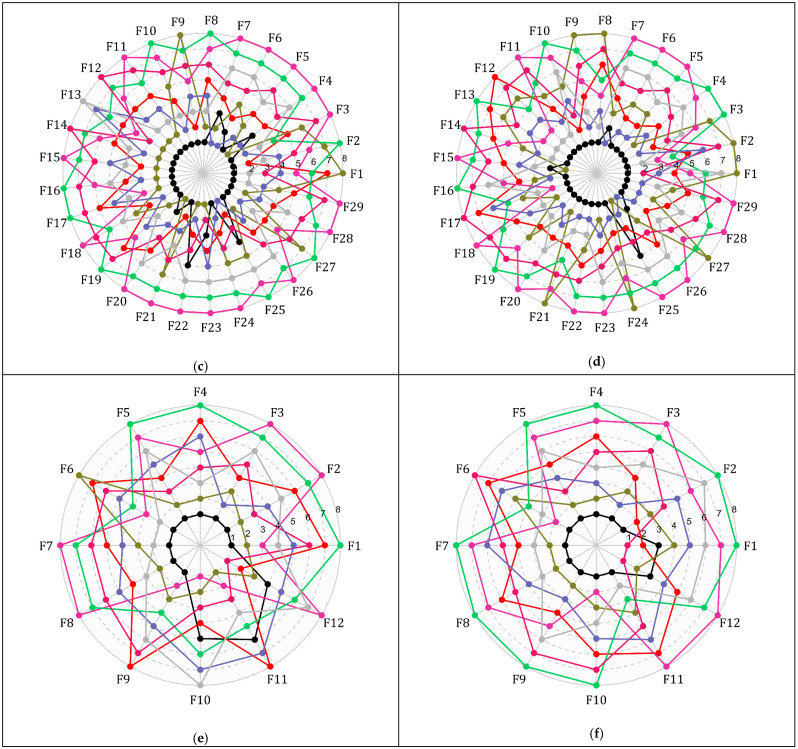
Rankings based on “Mean” of EMSMA and SMA variants on benchmark function. (**a**) Ranking of EMSMA and SMA variants (CEC2017 10D). (**b**) Ranking of EMSMA and SMA variants (CEC2017 30D). (**c**) Ranking of EMSMA and SMA variants (CEC2017 50D). (**d**) Ranking of EMSMA and SMA variants (CEC2017 100D). (**e**) Ranking of EMSMA and SMA variants (CEC2012 10D). (**f**) Ranking of EMSMA and SMA variants (CEC2017 20D).

Six SMA variants are used in this subsection for comparison with EMSMA. These SMA variants include DTSMA, ISMA, AOSMA, LSMA, ESMA, and MSMA. The parameter settings for the competitors are consistent with those suggested in the original literature, and the specific parameter settings are listed in Table 1. Detailed results for the EMSMA and the competitors for the CEC2017 and CEC2022 test sets are summarized in Table A7, Table A8, Table A9, Table A10, Table A11 and Table A12 in Appendix A. The rankings are based on the average values obtained for each algorithm in Table A7, Table A8, Table A9, Table A10, Table A11 and Table A12 in Appendix A. The rankings were used to draw spider plots to visualize the performance of EMSMA and the competitors on the benchmark function, as shown in Figure 5. The smaller the area enclosed by each curve in the graph, the better the performance of the algorithm represented by that curve. From Figure 5, it can be roughly concluded that EMSMA outperforms SMA and SMA variants in all cases. In the next few subsections, the results obtained will be further analyzed using statistical tests.

#### 4.5.1. Analysis Using the Wilcoxon Rank Sum Test

Wilcoxon rank sum test was used to check if there is a significant difference between two algorithms. In this paper, Wilcoxon rank sum test is applied to judge the difference between EMSMA and the competitor on each function. The results of the Wilcoxon rank sum test at the significance level a = 0.05, with respect to the CEC2017 and CEC2022 test suites, are recorded in Table A7, Table A8, Table A9, Table A10, Table A11 and Table A12 in Appendix A. Table 6 summarizes the number of EMSMA outperforms, resembles, and underperforms the competitors. EMSMA achieved the best scores of 1.47 and 1.67 on the CEC2017 and CEC2022 test sets, respectively, which is consistent with the analysis of the Wilcoxon rank sum test in Section 4.5.1.

From the results summarized in Table 6, it can be seen that the number of “+” is more than the number of “−” in all cases, which means that EMSMA significantly outperforms all competitors. It is worth noting that the gap between EMSMA and other competitors becomes larger as the dimensionality increases, which demonstrates the excellent performance of EMSMA under complex multi-dimensional problems. The test results are summarized below.

(a) For CEC2017 Dim = 10, EMSMA is superior (inferior) to SMA on 26(2) benchmark functions, DTSMA on 23(3) benchmark functions, ISMA on 26(2) benchmark functions, AOSMA on 29(0) benchmark functions, LSMA on 27(2) benchmark functions, ESMA on 27(2) benchmark functions, and MSMA on 28(1) benchmark functions.
(b) For CEC2017 Dim = 30, EMSMA is superior (inferior) to SMA on 23(2) benchmark functions, DTSMA on 18(4) benchmark functions, ISMA on 27(0) benchmark functions, AOSMA on 29(0) benchmark functions, LSMA on 22(0) benchmark functions, ESMA on 24(1) benchmark functions, and MSMA on 26(0) benchmark functions.
(c) For CEC2017 Dim = 50, EMSMA is superior (inferior) to SMA on 22(1) benchmark functions, DTSMA on 20(4) benchmark functions, ISMA on 29(0) benchmark functions, AOSMA on 29(0) benchmark functions, LSMA on 25(0) benchmark functions, ESMA on 21(1) benchmark functions, and MSMA on 28(0) benchmark functions.
(d) For CEC2017 Dim = 100, EMSMA is superior (inferior) to SMA on 27(0) benchmark functions, DTSMA on 24(1) benchmark functions, ISMA on 29(0) benchmark functions, AOSMA on 29(0) benchmark functions, LSMA on 28(0) benchmark functions, ESMA on 22(1) benchmark functions, and MSMA on 28(0) benchmark functions.
(e) For CEC2022 Dim = 10, EMSMA is superior (inferior) to SMA on 10(1) benchmark functions, DTSMA on 8(0) benchmark functions, ISMA on 10(2) benchmark functions, AOSMA on 10(1) benchmark functions, LSMA on 9(1) benchmark functions, ESMA on 11(0) benchmark functions, and MSMA on 11(0) benchmark functions.
(f) For CEC2022 Dim = 20, EMSMA is superior (inferior) to SMA on 10(0) benchmark functions, DTSMA on 10(0) benchmark functions, ISMA on 12(0) benchmark functions, AOSMA on 12(0) benchmark functions, LSMA on 10(0) benchmark functions, ESMA on 11(0) benchmark functions, and MSMA on 12(0) benchmark functions.

In conclusion, based on the Wilcoxon rank sum test results, it can be concluded that EMSMA has superior performance to DTSMA, ISMA, AOSMA, LSMA, ESMA, MSMA, and SMA.

#### 4.5.2. Analysis Using the Friedman Test

**Figure 6:**
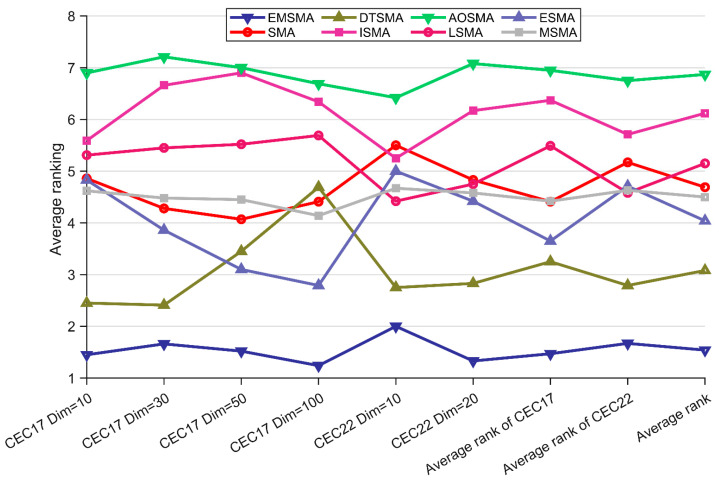
Rankings of EMSMA and SMA variants.

The Friedman test was also used to perform statistical analyses of the data in Table A7, Table A8, Table A9, Table A10, Table A11 and Table A12 for overall comparisons between EMSMA and competitors in addition to the two-by-two paired Wilcoxon test. Table 7 illustrates the Friedman scores as well as *p*-values for the EMSMA and SMA variants. It can be seen that the *p*-value of all Friedman tests is not greater than 0.05, which indicates that there is a significant difference in performance between EMSMA and competitors. Figure 6 visualizes the Friedman ranking of each algorithm.

**Figure 7:**
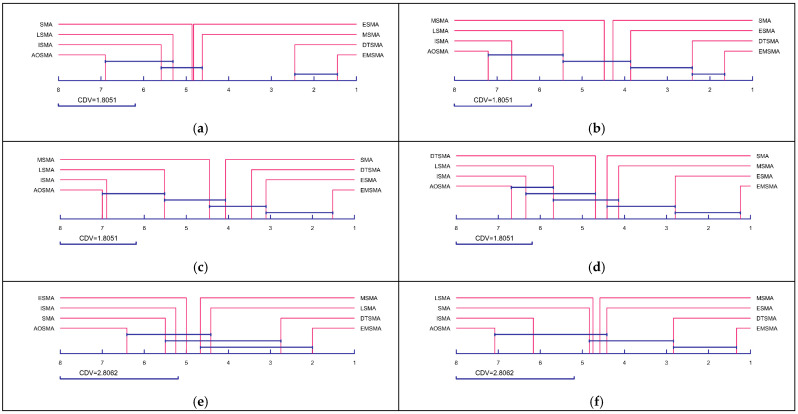
Differences between EMSMA and SMA variants. (**a**) CEC2017 10D. (**b**) CEC2017 30D. (**c**) CEC2017 50D. (**d**) CEC2017 100D. (**e**) CEC2022 10D. (**f**) CEC2022 20D.

As can be seen in Figure 6, EMSMA consistently achieves the best rankings in different scenarios, and the corresponding curves fluctuate very little, which indicates that EMSMA has good scalability and is able to show excellent performance in various scenarios. In addition, to further analyze the magnitude of differences between EMSMA and competitors. The Iman–Davenport test and the Nemenyi test are employed as post hoc tests based on the Friedman test [68]. Figure 7 presents the differences magnitude between EMSMA and the competitors, and there is no significant difference between the algorithms for CDV connection. As shown in Figure 7, EMSMA is not remarkably different from DTSMA and significantly outperforms the rest of the competitors on CEC2017 (Dim = 10/30) and CEC2022 (Dim = 10/20). EMSMA is superior to all SMA variants except ESMA on CEC2017 (Dim = 50/100). In conclusion, EMSMA further improves the performance of SMA and is significantly different from most SMA variants.

#### 4.5.3. Analysis Using the Convergence Curves

**Figure 8:**
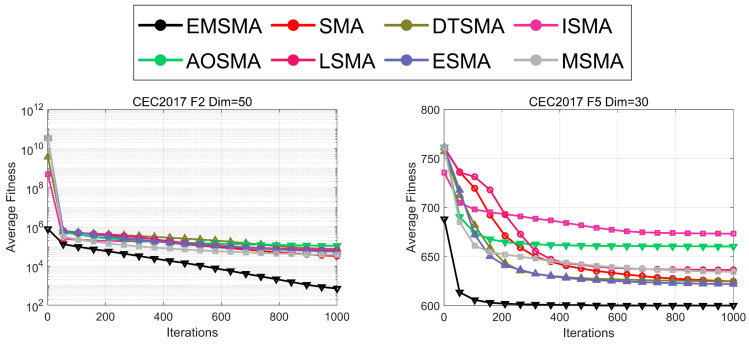

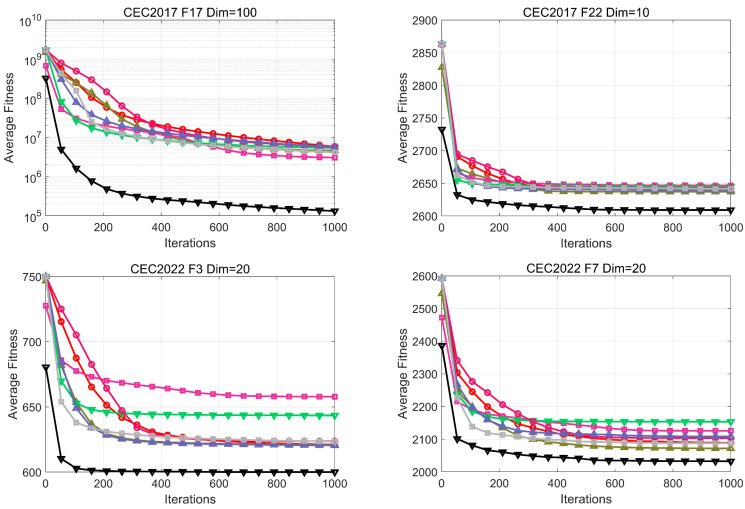
Convergence curves of EMSMA and SMA variants.

**Figure A1:**
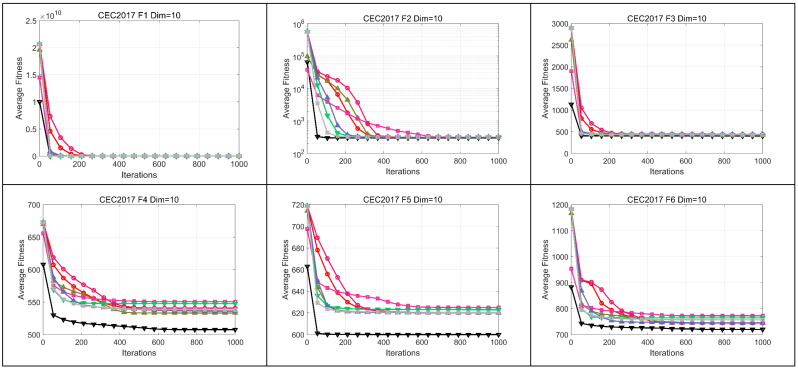

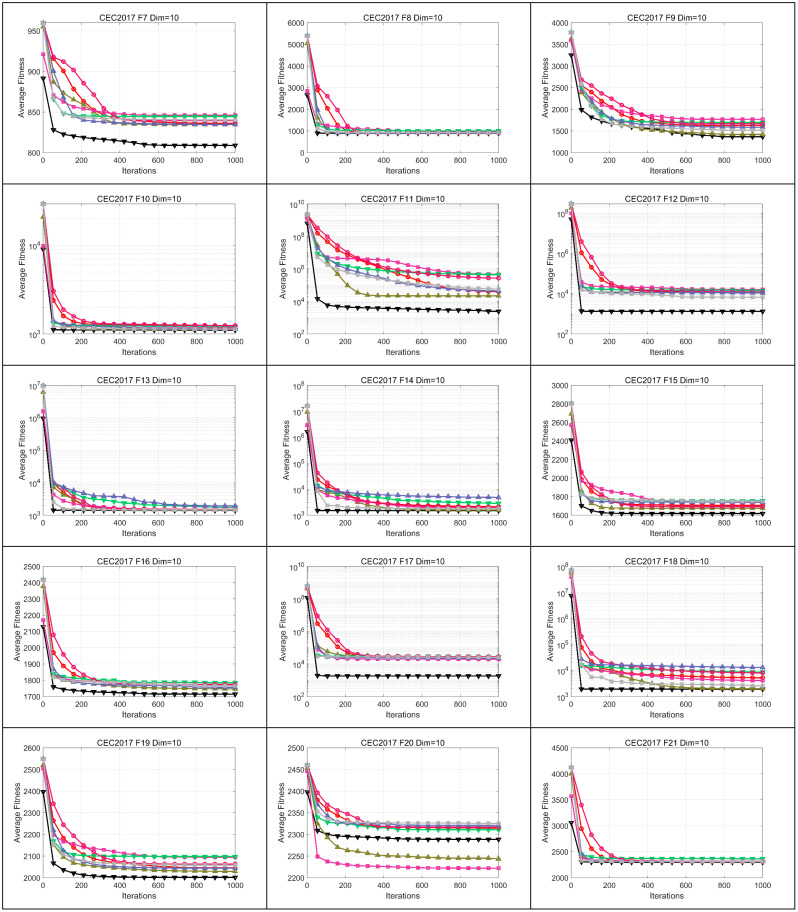

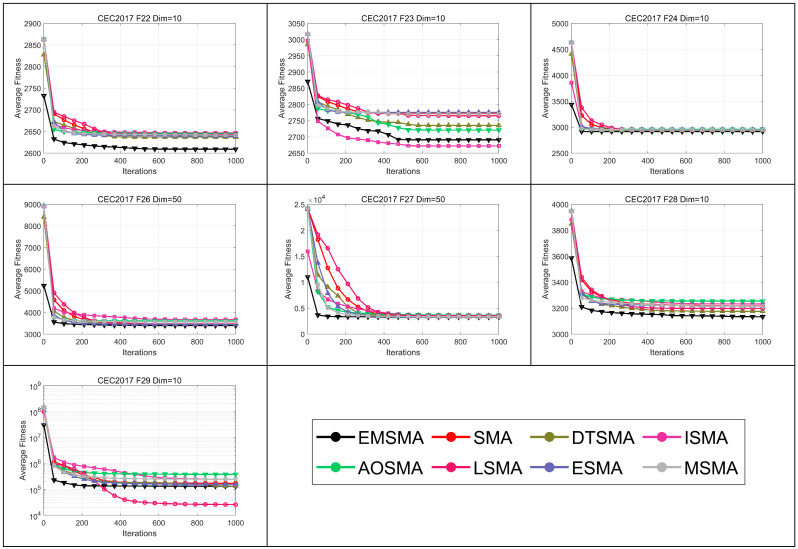
Convergence curves of EMSMA and SMA variants for CEC 2017 (Dim = 10).

**Figure A2:**
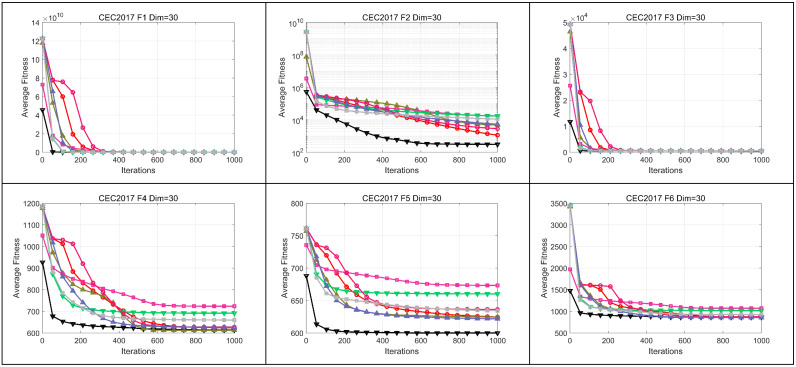

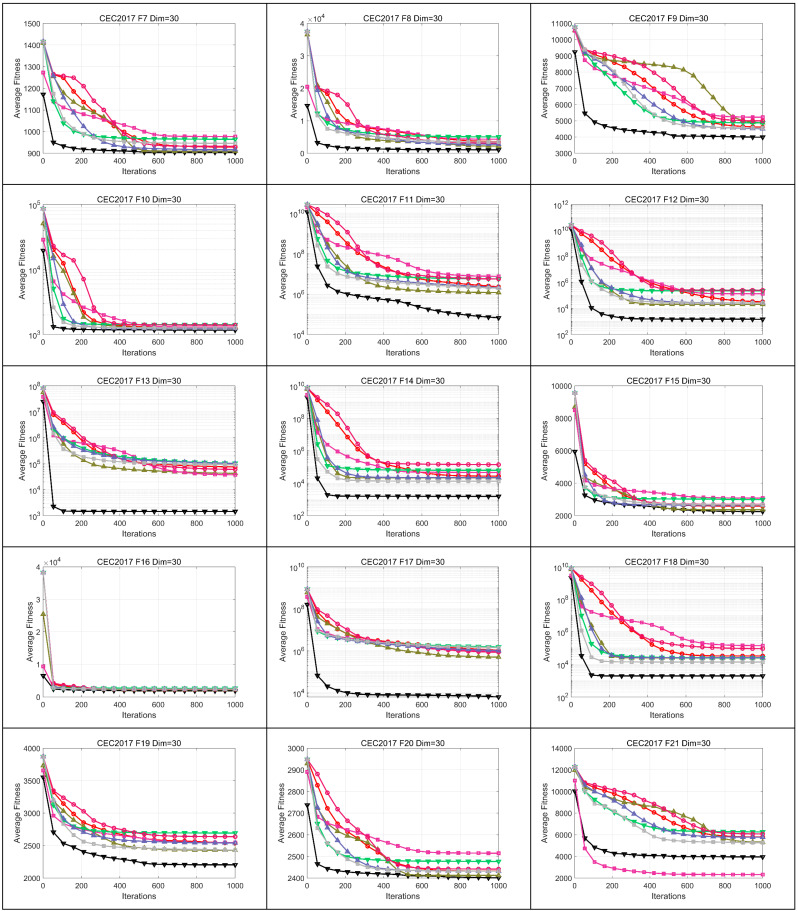

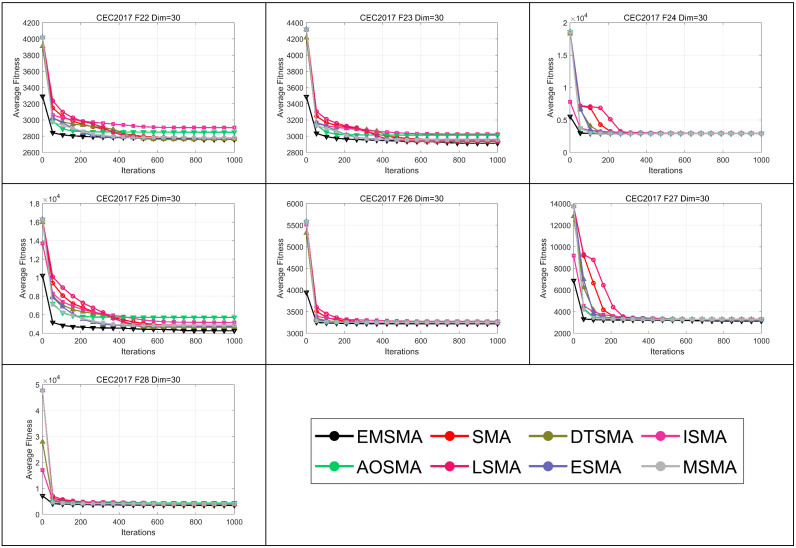
Convergence curves of EMSMA and SMA variants for CEC 2017 (Dim = 30).

**Figure A3:**
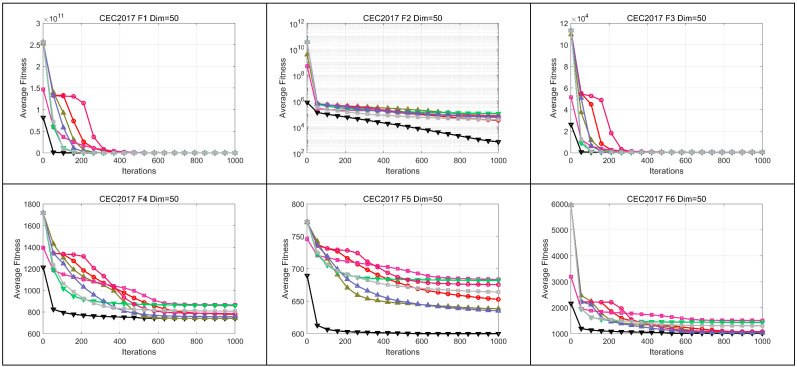

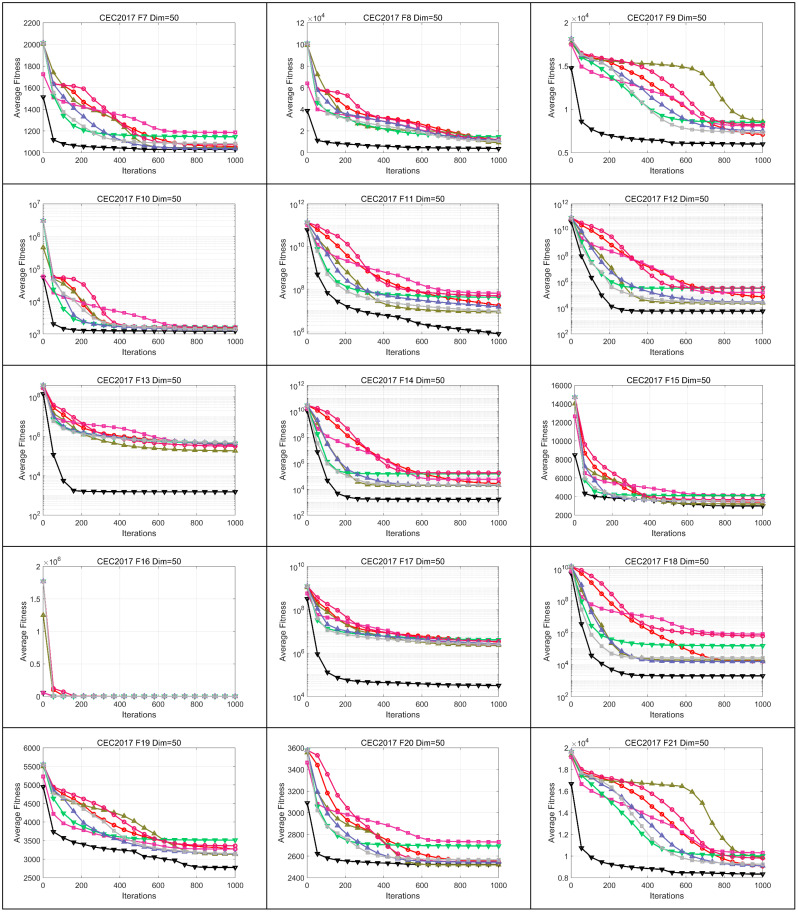

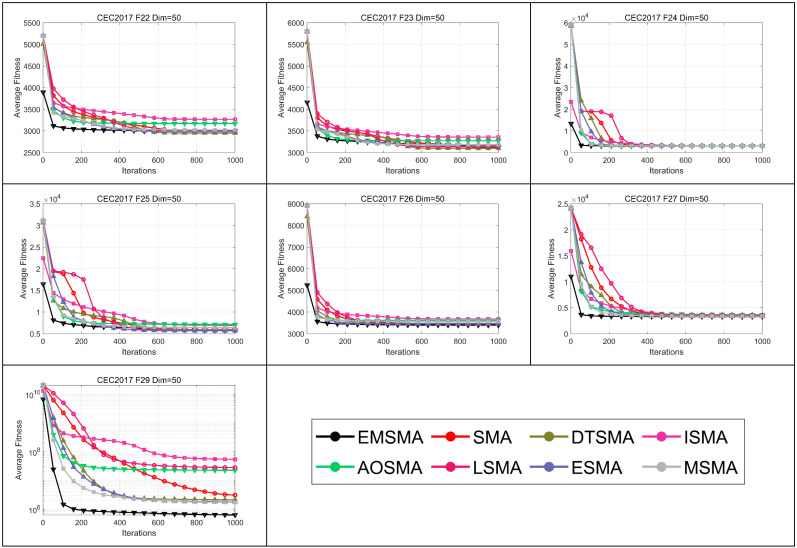
Convergence curves of EMSMA and SMA variants for CEC 2017 (Dim = 50).

**Figure A4:**
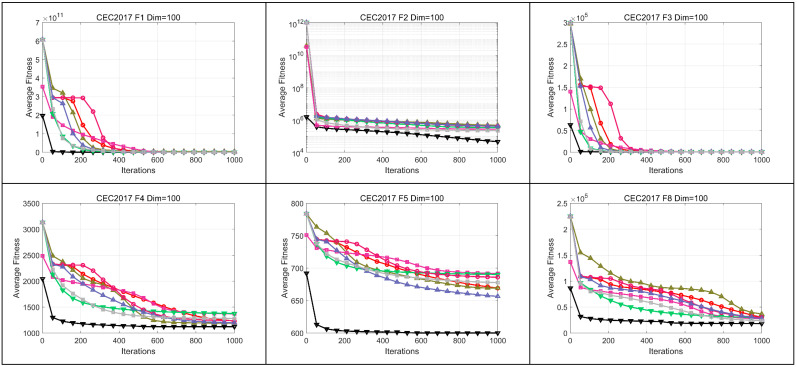

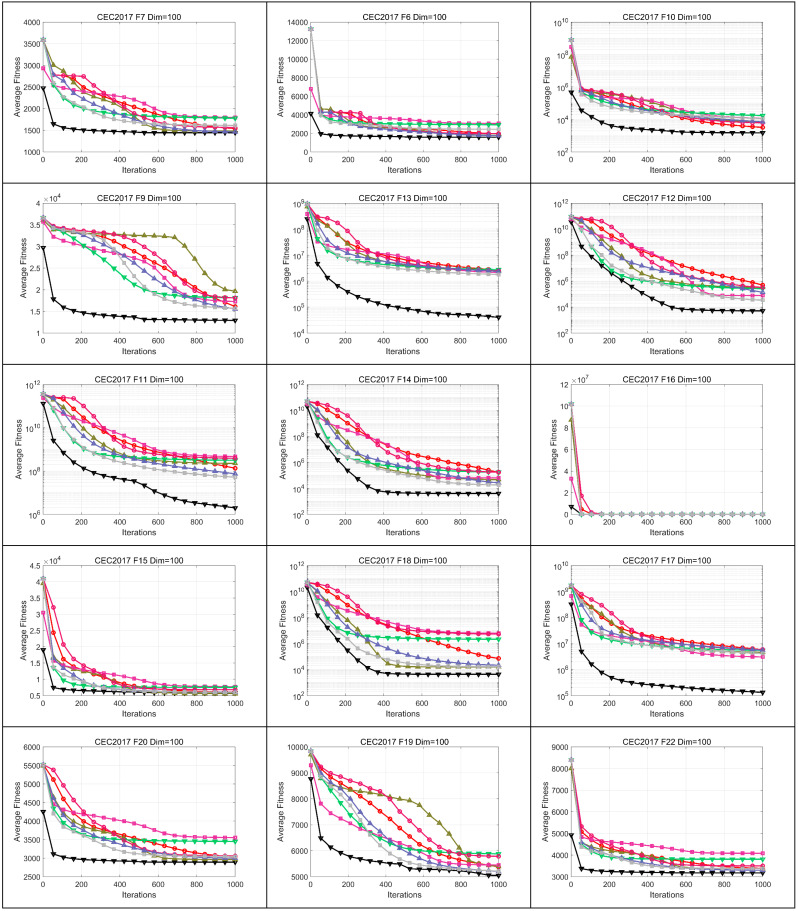

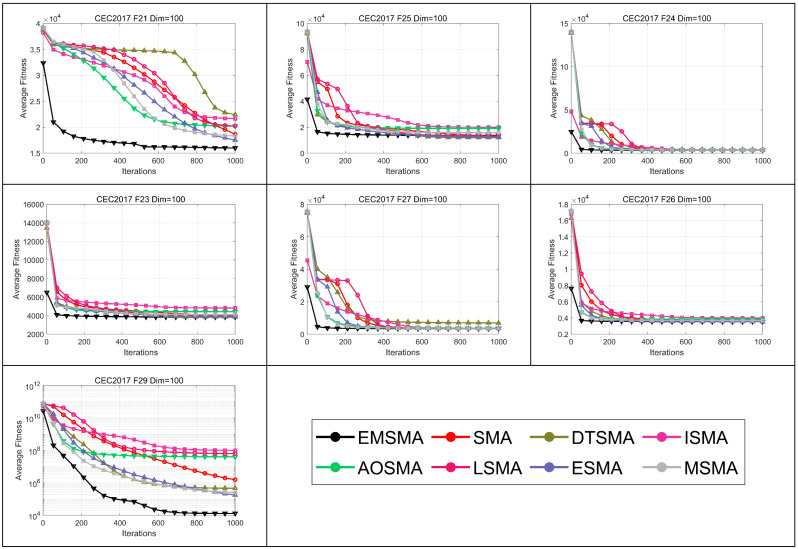
Convergence curves of EMSMA and SMA variants for CEC 2017 (Dim = 100).

**Figure A5:**
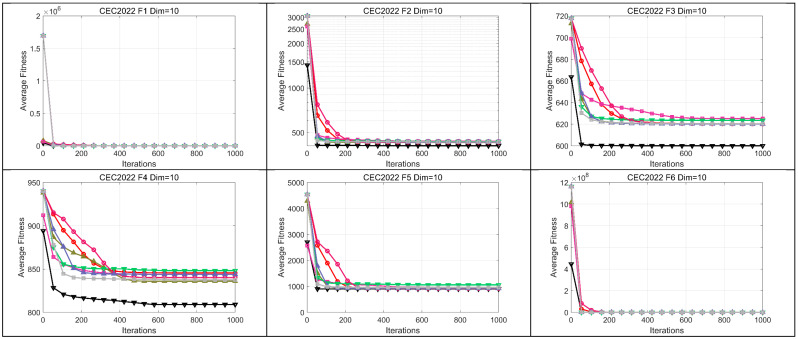

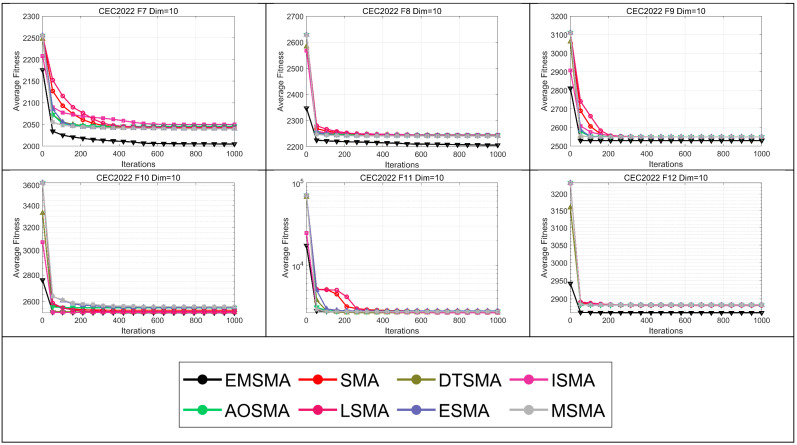
Convergence curves of EMSMA and SMA variants for CEC 2022 (Dim = 10).

**Figure A6:**
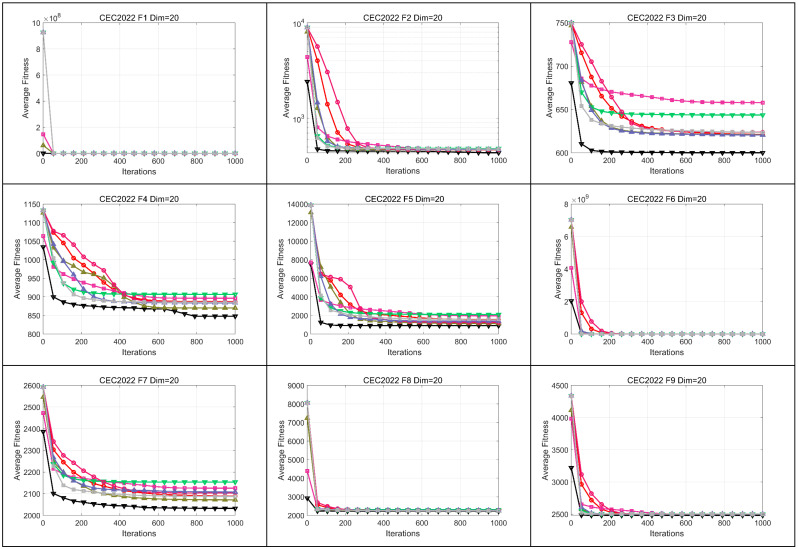

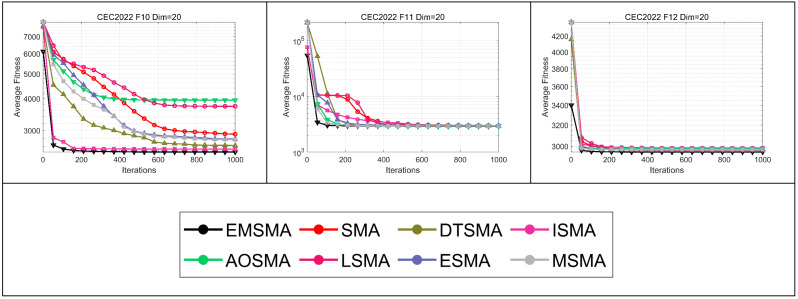
Convergence curves of EMSMA and SMA variants for CEC 2022 (Dim = 20).

In this subsection, the mean fitness convergence curves of 51 independent runs obtained by EMSMA and SMA variants are used to analyze the convergence performance of EMSMA. Since there are a total of 29 × 4 + 12 × 2 = 140 results for the two test suites, for simplicity, the convergence curves of the eight algorithms on the four functions of CEC2017 and the two functions of CEC2022 are shown in Figure 8. The rest of the convergence graphs can be obtained in Figure A1, Figure A2, Figure A3, Figure A4, Figure A5 and Figure A6 in Appendix B. As can be seen by analyzing Figure 8, EMSMA has the fastest convergence rate and the best convergence accuracy on the six functions. Therefore, it can be concluded that EMSMA has better convergence performance on the CEC2017 and CEC2022 test sets.

#### 4.5.4. Analysis Using the Box Plots

**Figure 9:**
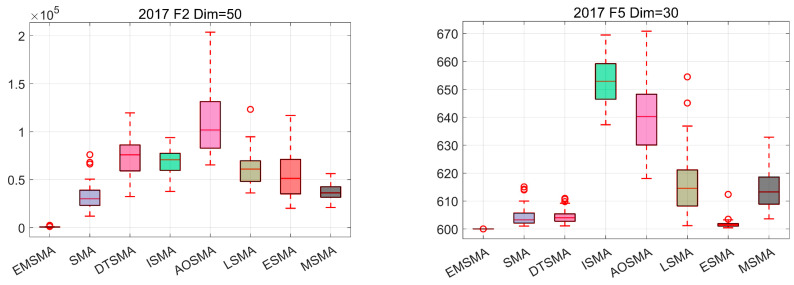

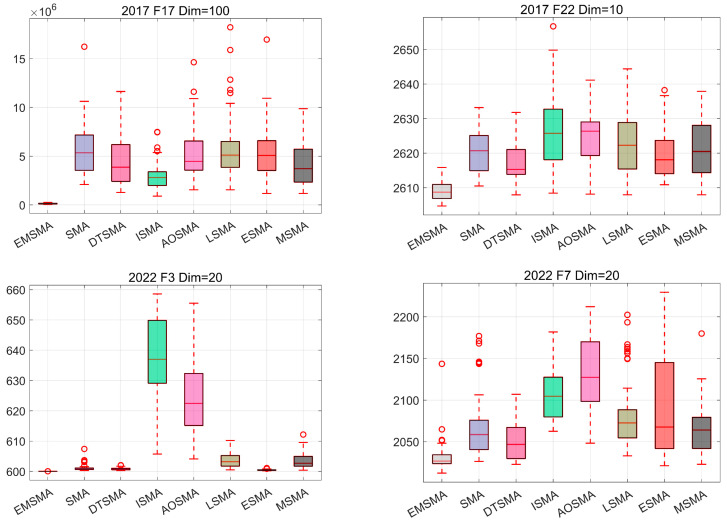
Boxplots of EMSMA and SMA variants.

**Figure A7:**
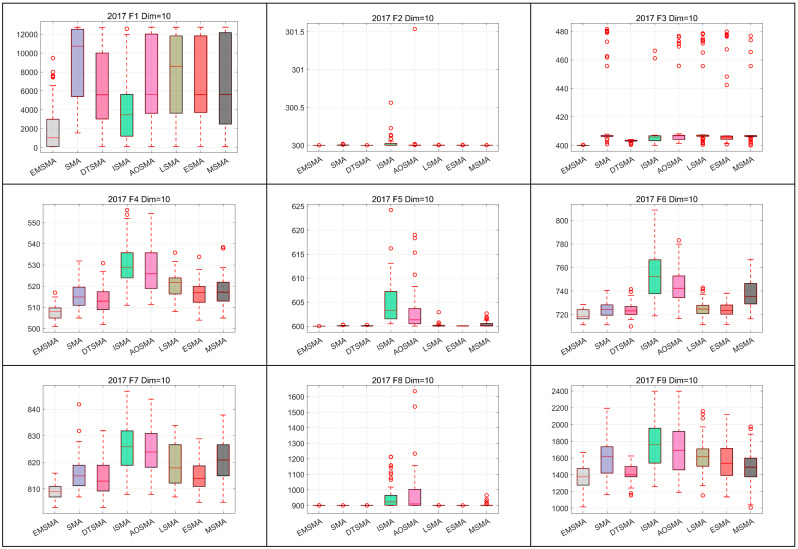

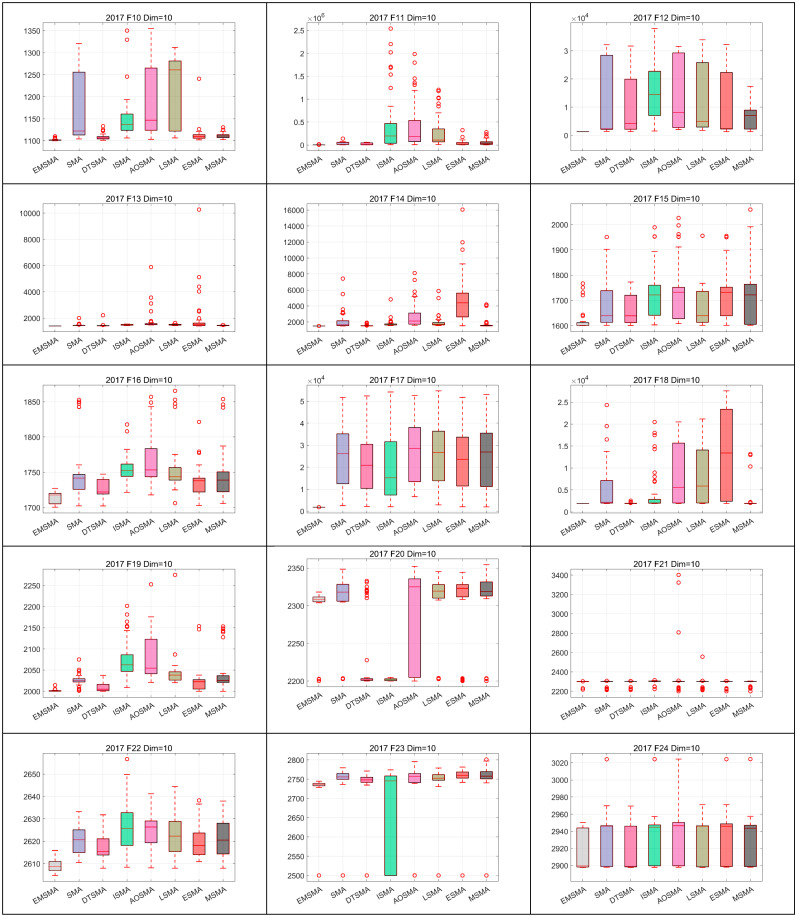

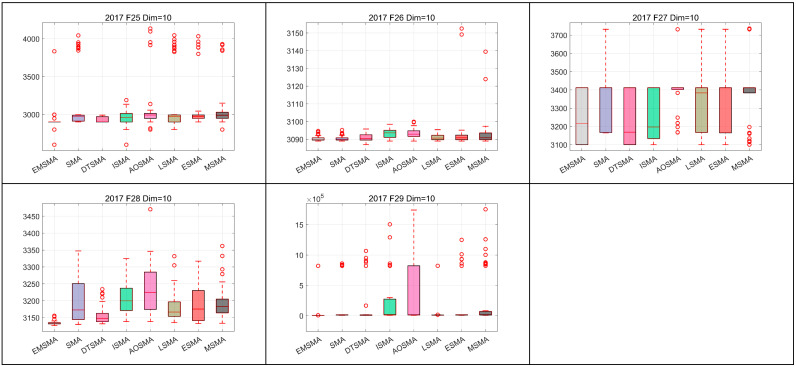
Boxplots of EMSMA and SMA variants for CEC 2017 (Dim = 10).

**Figure A8:**
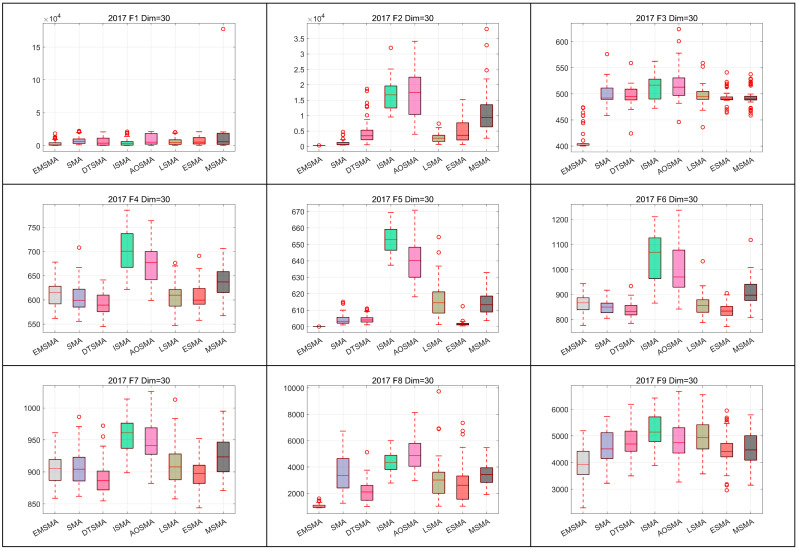

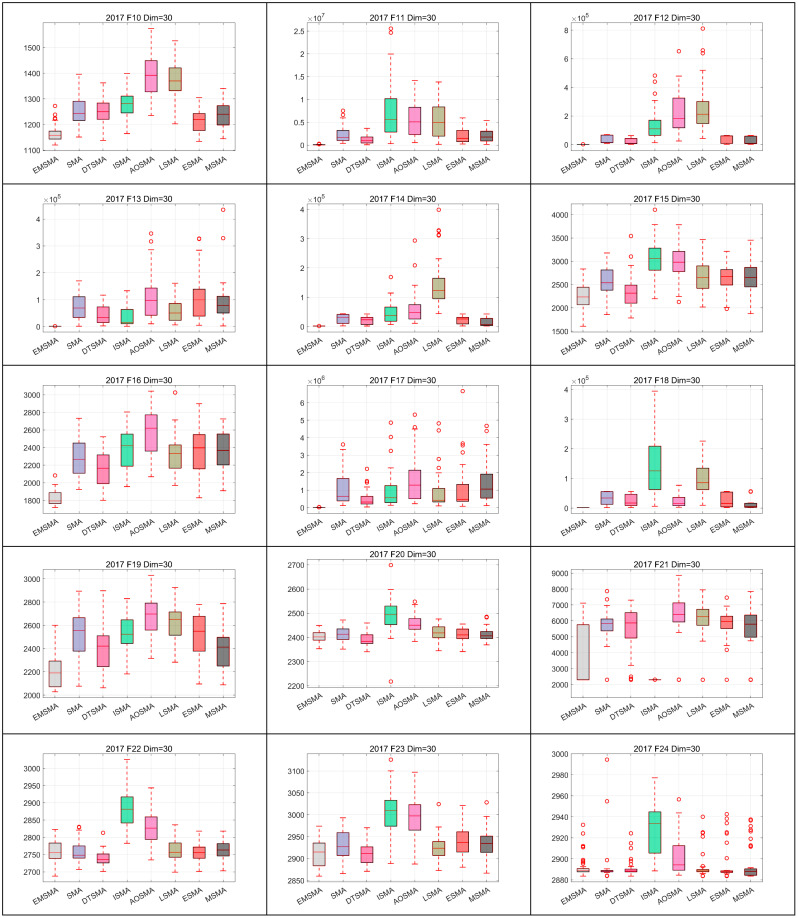

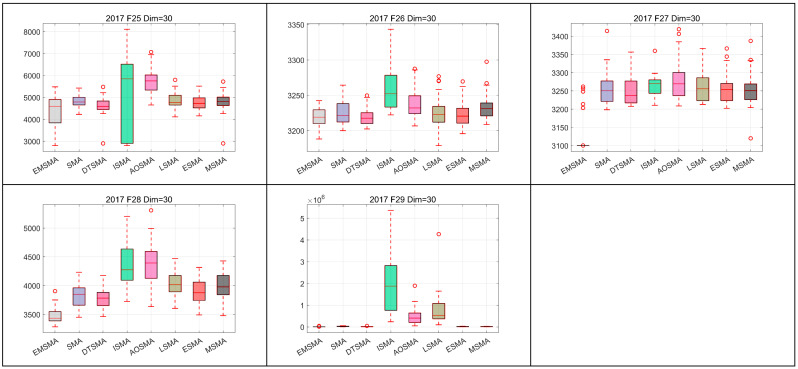
Boxplots of EMSMA and SMA variants for CEC 2017 (Dim = 30).

**Figure A9:**
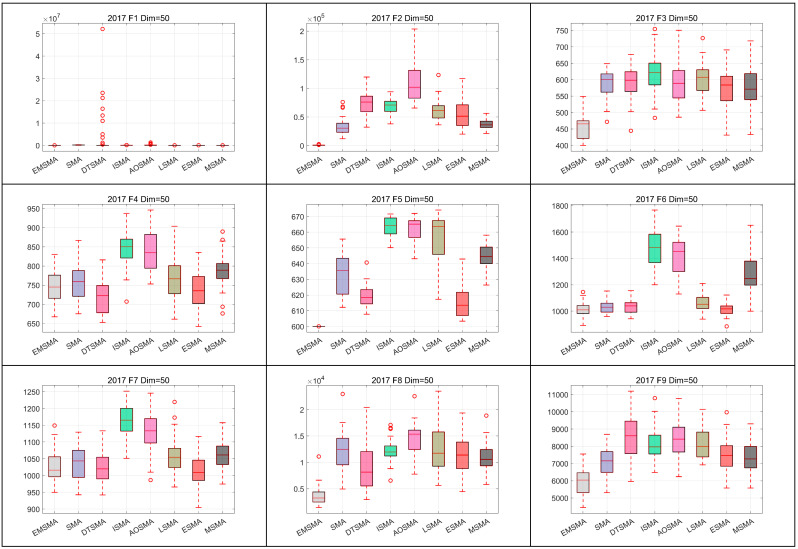

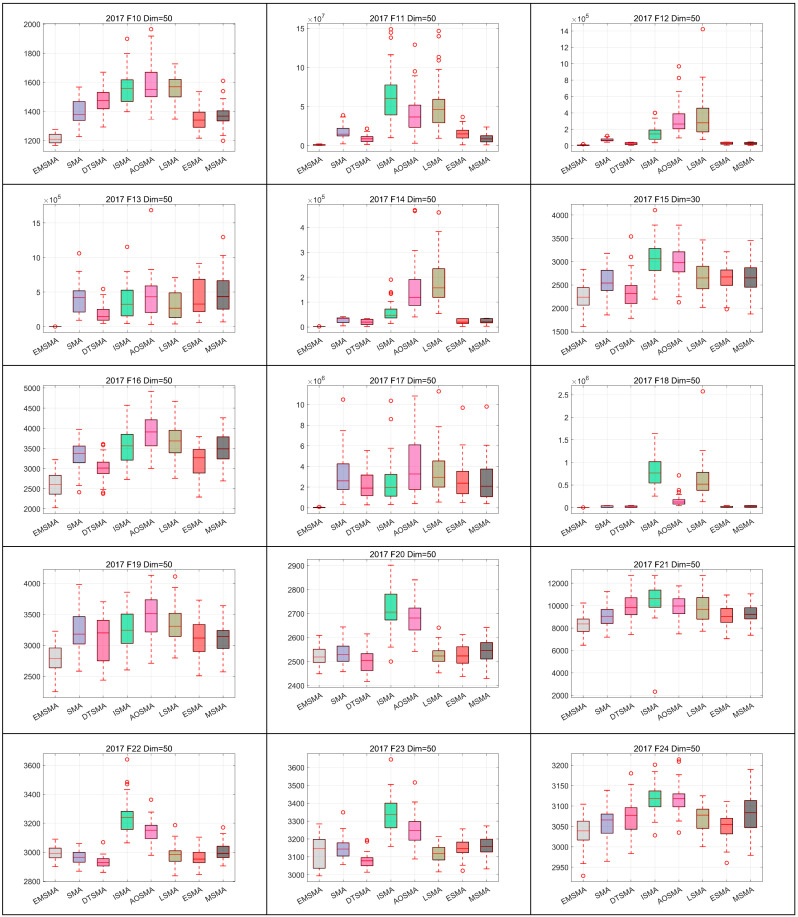

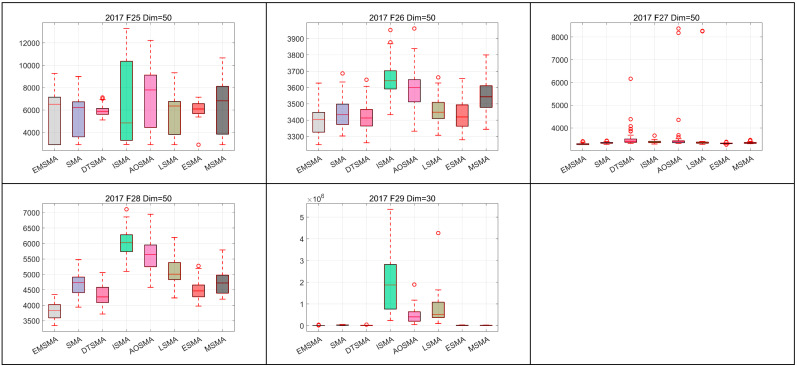
Boxplots of EMSMA and SMA variants for CEC 2017 (Dim = 50).

**Figure A10:**
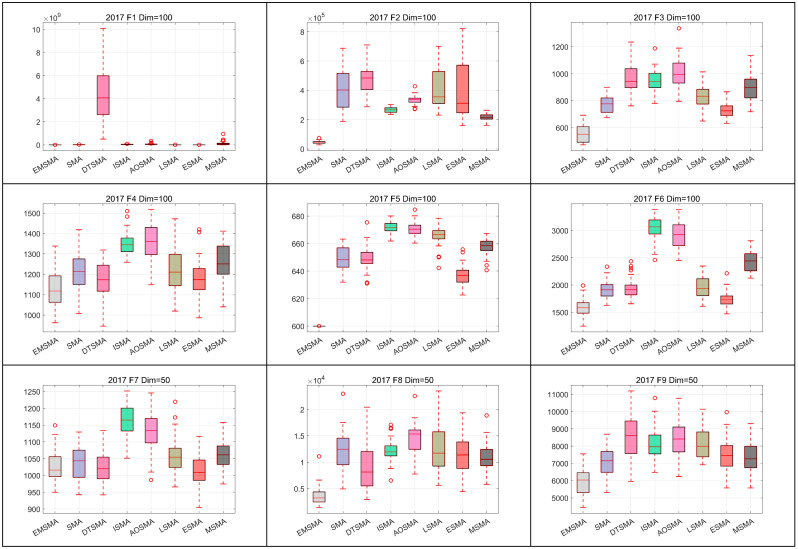

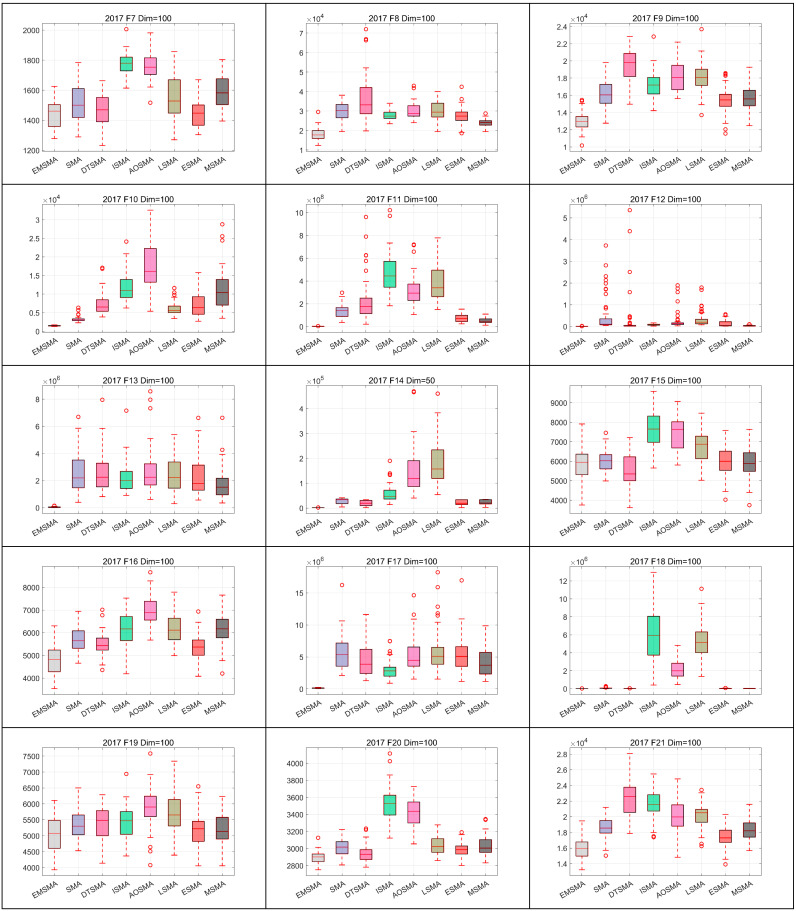

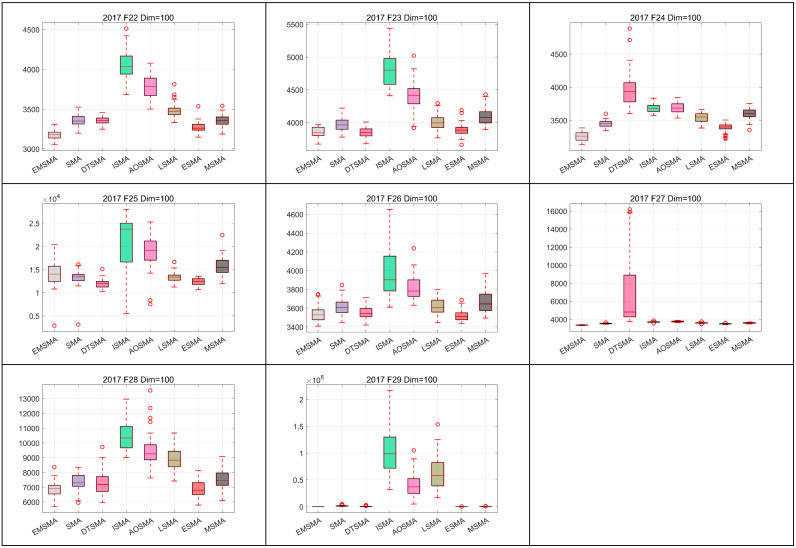
Boxplots of EMSMA and SMA variants for CEC 2017 (Dim = 100).

**Figure A11:**
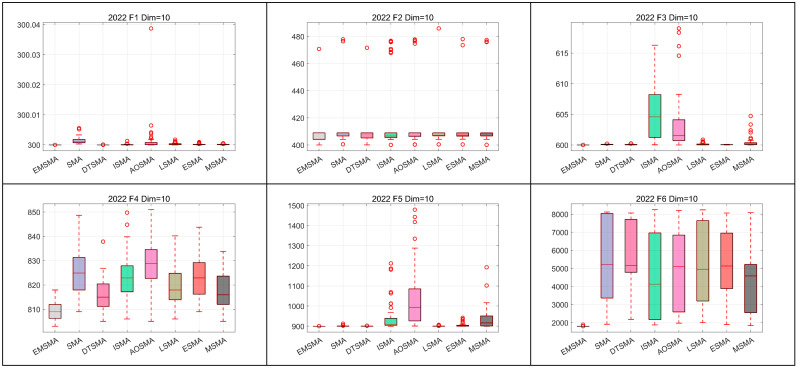

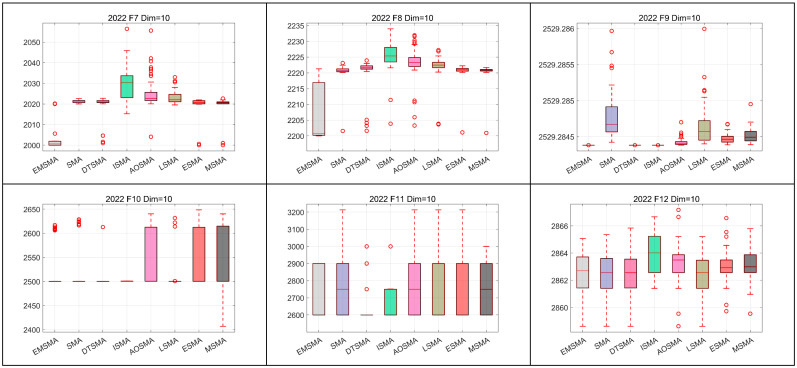
Boxplots of EMSMA and SMA variants for CEC 2022 (Dim = 10).

**Figure A12:**
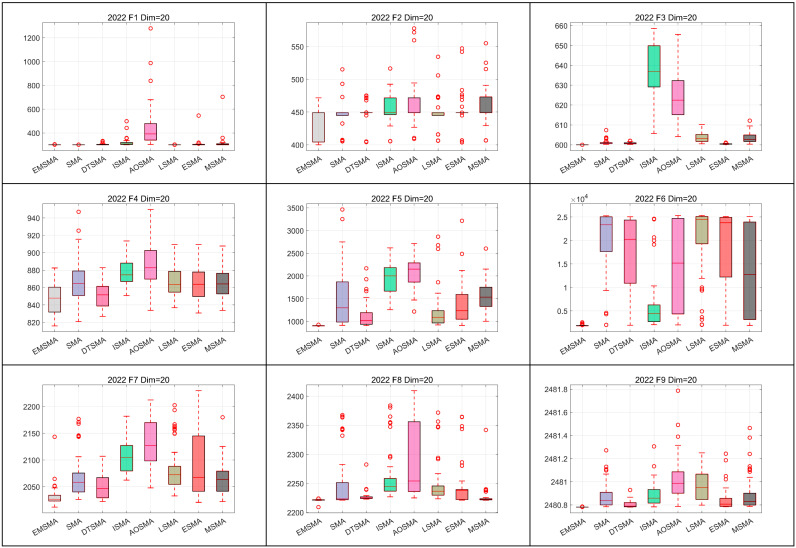

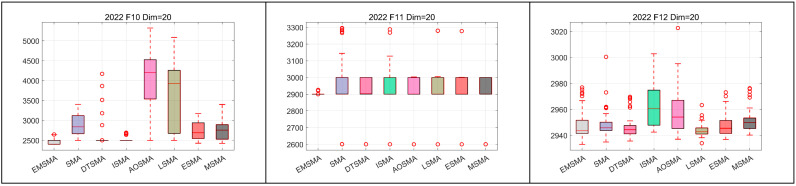
Boxplots of EMSMA and SMA variants for CEC 2022 (Dim = 20).

In addition to convergence analysis, robustness is also an important metric for algorithms. Box plots are often used to show how centralized the results are. Narrow box plots indicate that the results are more concentrated, and box plots with lower positions indicate that the algorithms achieve better quality solutions. Figure 9 illustrates the distribution of solutions for the eight algorithms when solving CEC2017 and CEC2022. Similar to Section 4.5.3, some of the functions are selected for display and the remaining graphs can be found in Figure A7, Figure A8, Figure A9, Figure A10, Figure A11 and Figure A12 in Appendix B. As can be seen in Figure 9, EMSMA provides higher-quality solutions with denser distributions, reflecting the robustness of EMSMA.

## 5. Conclusions

In this paper, we propose a variant of SMA called EMSMA with significant performance in different scenarios. Performance evaluation of the proposed EMSMA is carried out in this paper using the CEC2017 and CEC2022 test sets, and the effects of parameter selection and improvement strategies on the SMA are discussed. Comparison results with other SMA variants show that EMSMA has better convergence performance and robustness than DTSMA, ISMA, AOSMA, LSMA, ESMA, and MSMA. The leader covariance learning strategy, improved non-monopoly search mechanism, and random differential restart mechanism can significantly boost the SMA performance. In addition, the Wilcoxon rank sum test and Friedman test were utilized to confirm the significant difference between EMSMA and competitors.

In future work, more complex optimization problems will be applied to test the efficacy of EMSMA. For example, task planning, image segmentation, wireless sensor network coverage problems, feature selection, etc.

## Figures and Tables

**Table 1:** Parameter settings of eight algorithms.

Algorithms	Parameter Settings	Improvement Strategies
EMSMA	$z=0.3,\left|S\right|=0.5N$	LCLS; INSM; RDRM
SMA	z=0.03	N/A
DTSMA [61]	z=0.03,Cr=0.5,q=0.9	Dominant swarm; mutation mechanism, greedy strategy
ISMA [62]	$z=0.03,S_{\max}=N,S_{\min}=0.5N$	Chaotic opposition-based learning strategy; self-adaptive strategy; spiral search strategy
AOSMA [63]	z=0.03	Opposition-based learning; adaptive decision strategy
LSMA [54]	z=0.03	Leaders of the slime mould concentration
ESMA [64]	z=0.03	Hybridizing the equilibrium optimizer
MSMA [65]	z=0.03,E=100,Ns=10	Mutation strategy; gbest-guided mechanism; adaptive mutation probability

**Table 2:** Descriptions of CEC-2017 benchmark test functions.

No.		Functions	Search Range	Dim	f_min_
Unimodal functions	F1	Shifted and Rotated Bent Cigar Function	[−100,100]	10/30/50/100	100
	F2	Shifted and Rotated Zakharov Function	[−100,100]	10/30/50/100	300
Multimodal functions	F3	Shifted and Rotated Rosenbrock’s Function	[−100,100]	10/30/50/100	400
	F4	Shifted and Rotated Rastrigin’s Function	[−100,100]	10/30/50/100	500
	F5	Shifted and Rotated Expanded Scaffer’s F6 Function	[−100,100]	10/30/50/100	600
	F6	Shifted and Rotated Lunacek Bi_Rastrigin’s Function	[−100,100]	10/30/50/100	700
	F7	Shifted and Rotated Non-Continuous Rastrigin’s Function	[−100,100]	10/30/50/100	800
	F8	Shifted and Rotated Levy Function	[−100,100]	10/30/50/100	900
	F9	Shifted and Rotated Schwefel’s Function	[−100,100]	10/30/50/100	1000
Hybrid functions	F10	Hybrid Function 1 (N = 3)	[−100,100]	10/30/50/100	1100
	F11	Hybrid Function 2 (N = 3)	[−100,100]	10/30/50/100	1200
	F12	Hybrid Function 3 (N = 3)	[−100,100]	10/30/50/100	1300
	F13	Hybrid Function 4 (N = 4)	[−100,100]	10/30/50/100	1400
	F14	Hybrid Function 5 (N = 4)	[−100,100]	10/30/50/100	1500
	F15	Hybrid Function 6 (N = 4)	[−100,100]	10/30/50/100	1600
	F16	Hybrid Function 6 (N = 5)	[−100,100]	10/30/50/100	1700
	F17	Hybrid Function 6 (N = 5)	[−100,100]	10/30/50/100	1800
	F18	Hybrid Function 6 (N = 5)	[−100,100]	10/30/50/100	1900
	F19	Hybrid Function 6 (N = 6)	[−100,100]	10/30/50/100	2000
Composition functions	F20	Composition Function 1 (N = 3)	[−100,100]	10/30/50/100	2100
	F21	Composition Function 2 (N = 3)	[−100,100]	10/30/50/100	2200
	F22	Composition Function 3 (N = 4)	[−100,100]	10/30/50/100	2300
	F23	Composition Function 4 (N = 4)	[−100,100]	10/30/50/100	2400
	F24	Composition Function 5 (N = 5)	[−100,100]	10/30/50/100	2500
	F25	Composition Function 6 (N = 5)	[−100,100]	10/30/50/100	2600
	F26	Composition Function 7 (N = 6)	[−100,100]	10/30/50/100	2700
	F27	Composition Function 8 (N = 6)	[−100,100]	10/30/50/100	2800
	F28	Composition Function 9 (N = 3)	[−100,100]	10/30/50/100	2900
	F29	Composition Function 10 (N = 3)	[−100,100]	10/30/50/100	3000

**Table 3:** Descriptions of CEC-2022 benchmark test functions.

No.		Functions	Search Range	Dim	f_min_
Unimodal functions	F1	Shifted and full Rotated Zakharov Function	[−100,100]	10/20	300
Multimodal functions	F2	Shifted and full Rotated Rosenbrock’s Function	[−100,100]	10/20	400
	F3	Shifted and full Rotated Expanded Schaffer’s f6 Function	[−100,100]	10/20	600
	F4	Shifted and full Rotated Non-Continuous Rastrigin’s Function	[−100,100]	10/20	800
	F5	Shifted and full Rotated Levy Function	[−100,100]	10/20	900
Hybrid functions	F6	Hybrid Function 1 (N = 3)	[−100,100]	10/20	1800
	F7	Hybrid Function 2 (N = 6	[−100,100]	10/20	2000
	F8	Hybrid Function 3 (N = 5)	[−100,100]	10/20	2200
Composition functions	F9	Composition Function 1 (N = 5)	[−100,100]	10/20	2300
	F10	Composition Function 2 (N = 4)	[−100,100]	10/20	2400
	F11	Composition Function 3 (N = 5)	[−100,100]	10/20	2600
	F12	Composition Function 4 (N = 6)	[−100,100]	10/20	2700

**Table 4:** Performance comparison of EMSMA with different parameter z.

Test Suite	Dim	SMA	z = 0.1	z = 0.2	z = 0.3	z = 0.4	z = 0.5	z = 0.6	z = 0.7	*p*-Value
CEC2017	10	7.41	3.48	3.55	3.72	4.38	4.62	4.31	4.52	2.81E-09
	30	7.14	4.41	4.62	4.38	4.10	4.52	3.41	3.41	8.83E-08
	50	7.34	5.52	5.07	4.07	3.69	3.69	3.31	3.31	7.14E-12
	100	7.69	5.59	5.45	3.41	4.62	3.62	3.38	2.24	1.39E-18
CEC2022	10	6.75	2.58	3.00	3.25	4.58	5.50	5.21	5.13	1.49E-04
	20	7.50	3.75	4.67	4.33	3.50	3.58	3.83	4.83	1.13E-03
Average rank of CEC2017		7.40	4.75	4.67	3.90	4.20	4.11	3.60	3.37	2.28E-08
Average rank of CEC2022		7.13	3.17	3.83	3.79	4.04	4.54	4.52	4.98	6.38E-04
Average rank of total		7.31	4.22	4.39	3.86	4.15	4.26	3.91	3.91	2.13E-04

**Table 5:** The rankings of EMSMA and its variants.

Algorithm	CEC2017 Test Suite				CEC2022 Test Suite		Average Rank of CEC2017	Average Rank of CEC2022	Average Rank of Total
	10	30	50	100	10	20			
SMA	4.52	4.48	4.41	4.59	4.25	4.50	4.50	4.38	4.45
EMSMA-1	2.28	2.66	2.83	3.03	2.00	2.08	2.70	2.04	2.45
EMSMA-2	3.90	3.38	3.14	2.69	4.00	3.25	3.28	3.63	3.41
EMSMA-3	2.45	2.45	2.69	2.90	2.50	3.08	2.62	2.79	2.68
EMSMA	1.86	2.03	1.93	1.79	2.25	2.08	1.91	2.17	2.00
*p*-value	2.11E-12	1.09E-08	1.06E-07	1.30E-09	3.17E-04	7.18E-04	N/A	N/A	N/A

**Table 6:** The results of Wilcoxon rank sum test between EMSMA and competitors (a = 0.05).

EMSMA vs. +/=/−	CEC2017 Test Suite				CEC2022 Test Suite	
	Dim = 10	Dim = 30	Dim = 50	Dim = 100	Dim = 10	Dim = 20
SMA	26/1/2	23/4/2	22/6/1	27/2/0	10/1/1	10/2/0
DTSMA	23/3/3	18/7/4	20/5/4	24/4/1	8/4/0	10/2/0
ISMA	26/1/2	27/2/0	29/0/0	29/0/0	10/0/2	12/0/0
AOSMA	29/0/0	29/0/0	29/0/0	29/0/0	10/1/1	12/0/0
LSMA	27/0/2	22/7/0	25/4/0	28/1/0	9/2/1	10/2/0
ESMA	27/0/2	24/4/1	21/7/1	22/6/1	11/1/0	11/1/0
MSMA	28/0/1	26/3/0	28/1/0	27/2/0	11/1/0	12/0/0

**Table 7:** The results of Friedman test between EMSMA and competitors (a = 0.05).

Algorithm	CEC2017				CEC2022 with		Average Rank of CEC2017	Average Rank of CEC2022	Average Rank of Total
	10	30	50	100	10	20			
EMSMA	1.45	1.66	1.52	1.24	2.00	1.33	1.47	1.67	1.54
SMA	4.86	4.28	4.07	4.41	5.50	4.83	4.41	5.17	4.69
DTSMA	2.45	2.41	3.45	4.69	2.75	2.83	3.25	2.79	3.08
ISMA	5.59	6.66	6.90	6.34	5.25	6.17	6.37	5.71	6.12
AOSMA	6.90	7.21	7.00	6.69	6.42	7.08	6.95	6.75	6.87
LSMA	5.31	5.45	5.52	5.69	4.42	4.75	5.49	4.58	5.15
ESMA	4.83	3.86	3.10	2.79	5.00	4.42	3.65	4.71	4.04
MSMA	4.62	4.48	4.45	4.14	4.67	4.58	4.42	4.63	4.50
*p*-value	2.33E-19	8.53E-24	3.46E-23	2.51E-21	1.09E-04	1.44E-07	N/A	N/A	N/A

## Data Availability

The datasets generated during and/or analyzed during the current study are available from the corresponding author upon reasonable request.

## Appendix A

**Table A1:** Statistical results obtained from EMSMA and EMSMA variants for CEC 2017 (Dim = 10).

Function	Index	SMA	EMSMA-1	EMSMA-2	EMSMA-3	EMSMA
F1	Best	1.55E+03	1.00E+02	1.00E+02	1.00E+02	1.00E+02
	Mean	8.60E+03	6.53E+02	1.56E+03	2.18E+03	2.13E+03
	Std	3.92E+03	2.44E+03	1.77E+03	2.87E+03	2.56E+03
F2	Best	3.00E+02	3.00E+02	3.00E+02	3.00E+02	3.00E+02
	Mean	3.00E+02	3.00E+02	3.00E+02	3.00E+02	3.00E+02
	Std	3.36E-03	2.64E-06	8.75E-02	1.03E-02	3.92E-07
F3	Best	4.01E+02	4.00E+02	4.00E+02	4.00E+02	4.00E+02
	Mean	4.19E+02	4.00E+02	4.06E+02	4.05E+02	4.00E+02
	Std	2.75E+01	1.14E-01	1.08E+01	1.54E+00	8.39E-02
F4	Best	5.05E+02	5.04E+02	5.06E+02	5.01E+02	5.01E+02
	Mean	5.16E+02	5.11E+02	5.15E+02	5.06E+02	5.08E+02
	Std	7.28E+00	4.09E+00	6.76E+00	2.45E+00	3.25E+00
F5	Best	6.00E+02	6.00E+02	6.00E+02	6.00E+02	6.00E+02
	Mean	6.00E+02	6.00E+02	6.00E+02	6.00E+02	6.00E+02
	Std	4.45E-02	6.48E-03	7.43E-03	1.41E-02	1.04E-03
F6	Best	7.11E+02	7.12E+02	7.11E+02	7.10E+02	7.11E+02
	Mean	7.24E+02	7.21E+02	7.26E+02	7.16E+02	7.20E+02
	Std	6.18E+00	5.17E+00	9.17E+00	2.18E+00	4.57E+00
F7	Best	8.07E+02	8.03E+02	8.06E+02	8.02E+02	8.03E+02
	Mean	8.16E+02	8.11E+02	8.17E+02	8.06E+02	8.09E+02
	Std	6.80E+00	4.98E+00	6.33E+00	2.69E+00	3.07E+00
F8	Best	9.00E+02	9.00E+02	9.00E+02	9.00E+02	9.00E+02
	Mean	9.00E+02	9.00E+02	9.00E+02	9.00E+02	9.00E+02
	Std	2.43E-02	4.37E-06	2.76E-01	8.11E-02	1.09E-01
F9	Best	1.16E+03	1.02E+03	1.14E+03	1.02E+03	1.02E+03
	Mean	1.60E+03	1.36E+03	1.58E+03	1.33E+03	1.37E+03
	Std	2.49E+02	1.64E+02	2.15E+02	1.22E+02	1.55E+02
F10	Best	1.10E+03	1.10E+03	1.10E+03	1.10E+03	1.10E+03
	Mean	1.17E+03	1.10E+03	1.11E+03	1.10E+03	1.10E+03
	Std	7.47E+01	1.67E+00	3.58E+00	1.92E+00	1.91E+00
F11	Best	2.79E+03	1.20E+03	1.82E+03	1.21E+03	1.20E+03
	Mean	4.00E+04	1.27E+03	2.23E+04	3.50E+03	2.40E+03
	Std	2.64E+04	5.65E+01	2.32E+04	5.01E+03	2.33E+03
F12	Best	1.39E+03	1.30E+03	1.30E+03	1.30E+03	1.30E+03
	Mean	1.21E+04	1.31E+03	2.14E+03	1.31E+03	1.31E+03
	Std	1.28E+04	3.07E+00	3.45E+03	3.27E+00	3.48E+00
F13	Best	1.43E+03	1.40E+03	1.40E+03	1.40E+03	1.40E+03
	Mean	1.46E+03	1.40E+03	1.41E+03	1.40E+03	1.40E+03
	Std	8.22E+01	2.44E+00	5.39E+00	2.23E+00	1.30E+00
F14	Best	1.51E+03	1.50E+03	1.50E+03	1.50E+03	1.50E+03
	Mean	2.11E+03	1.50E+03	1.57E+03	1.50E+03	1.50E+03
	Std	1.06E+03	4.71E-01	4.44E+02	2.04E+00	8.30E-01
F15	Best	1.60E+03	1.60E+03	1.60E+03	1.60E+03	1.60E+03
	Mean	1.69E+03	1.62E+03	1.67E+03	1.61E+03	1.62E+03
	Std	8.85E+01	3.93E+01	9.20E+01	2.18E+01	3.84E+01
F16	Best	1.70E+03	1.70E+03	1.70E+03	1.70E+03	1.70E+03
	Mean	1.75E+03	1.72E+03	1.72E+03	1.71E+03	1.71E+03
	Std	3.81E+01	7.84E+00	1.93E+01	8.46E+00	8.05E+00
F17	Best	2.50E+03	1.80E+03	1.80E+03	1.80E+03	1.80E+03
	Mean	2.60E+04	1.80E+03	4.17E+03	1.80E+03	1.80E+03
	Std	1.39E+04	2.92E+00	4.66E+03	6.71E+00	1.03E+00
F18	Best	1.91E+03	1.90E+03	1.90E+03	1.90E+03	1.90E+03
	Mean	5.16E+03	1.90E+03	1.90E+03	1.90E+03	1.90E+03
	Std	5.11E+03	6.56E-01	4.29E+00	7.95E-01	5.99E-01
F19	Best	2.00E+03	2.00E+03	2.00E+03	2.00E+03	2.00E+03
	Mean	2.03E+03	2.00E+03	2.00E+03	2.01E+03	2.00E+03
	Std	1.25E+01	2.43E+00	5.57E+00	8.12E+00	2.27E+00
F20	Best	2.20E+03	2.20E+03	2.20E+03	2.20E+03	2.20E+03
	Mean	2.29E+03	2.28E+03	2.29E+03	2.28E+03	2.29E+03
	Std	5.20E+01	5.03E+01	4.68E+01	4.76E+01	4.37E+01
F21	Best	2.21E+03	2.20E+03	2.22E+03	2.20E+03	2.22E+03
	Mean	2.29E+03	2.29E+03	2.30E+03	2.29E+03	2.30E+03
	Std	2.75E+01	2.83E+01	1.41E+01	2.20E+01	1.51E+01
F22	Best	2.61E+03	2.60E+03	2.61E+03	2.30E+03	2.60E+03
	Mean	2.62E+03	2.61E+03	2.62E+03	2.60E+03	2.61E+03
	Std	5.96E+00	3.60E+00	7.70E+00	4.35E+01	3.01E+00
F23	Best	2.50E+03	2.50E+03	2.50E+03	2.50E+03	2.50E+03
	Mean	2.75E+03	2.73E+03	2.64E+03	2.70E+03	2.69E+03
	Std	3.75E+01	5.75E+01	1.21E+02	8.69E+01	9.53E+01
F24	Best	2.90E+03	2.90E+03	2.90E+03	2.90E+03	2.90E+03
	Mean	2.93E+03	2.92E+03	2.93E+03	2.93E+03	2.92E+03
	Std	2.93E+01	2.28E+01	2.33E+01	2.11E+01	2.28E+01
F25	Best	2.90E+03	2.80E+03	2.60E+03	2.90E+03	2.60E+03
	Mean	3.12E+03	2.92E+03	2.96E+03	2.91E+03	2.92E+03
	Std	3.74E+02	1.28E+02	2.33E+02	2.38E+01	1.42E+02
F26	Best	3.09E+03	3.09E+03	3.09E+03	3.09E+03	3.09E+03
	Mean	3.09E+03	3.09E+03	3.09E+03	3.09E+03	3.09E+03
	Std	1.31E+00	3.32E-01	1.03E+01	1.66E+00	1.38E+00
F27	Best	3.16E+03	3.10E+03	3.10E+03	3.10E+03	3.10E+03
	Mean	3.32E+03	3.30E+03	3.32E+03	3.29E+03	3.25E+03
	Std	1.44E+02	1.82E+02	1.50E+02	1.27E+02	1.50E+02
F28	Best	3.13E+03	3.13E+03	3.13E+03	3.13E+03	3.13E+03
	Mean	3.20E+03	3.13E+03	3.18E+03	3.13E+03	3.13E+03
	Std	6.40E+01	4.34E+00	4.14E+01	4.39E+00	5.32E+00
F29	Best	6.14E+03	3.40E+03	3.57E+03	3.46E+03	3.42E+03
	Mean	1.71E+05	3.55E+04	1.63E+05	2.29E+05	1.32E+05
	Std	3.28E+05	1.60E+05	3.37E+05	3.96E+05	3.00E+05

**Table A2:** Statistical results obtained from EMSMA and EMSMA variants for CEC 2017 (Dim = 30).

Function	Index	SMA	EMSMA-1	EMSMA-2	EMSMA-3	EMSMA
F1	Best	1.04E+03	1.03E+02	1.10E+02	5.43E+02	1.09E+02
	Mean	8.18E+03	3.28E+03	3.37E+03	1.38E+04	3.60E+03
	Std	6.79E+03	3.94E+03	3.60E+03	1.22E+04	4.28E+03
F2	Best	3.81E+02	3.00E+02	3.01E+02	3.11E+02	3.01E+02
	Mean	1.11E+03	3.02E+02	3.10E+02	3.59E+02	3.05E+02
	Std	9.08E+02	7.49E-01	6.13E+00	5.79E+01	3.07E+00
F3	Best	4.59E+02	4.04E+02	4.00E+02	4.71E+02	4.00E+02
	Mean	4.98E+02	4.70E+02	4.19E+02	5.03E+02	4.12E+02
	Std	1.87E+01	3.84E+01	2.60E+01	1.99E+01	2.13E+01
F4	Best	5.56E+02	5.30E+02	5.43E+02	5.24E+02	5.62E+02
	Mean	6.05E+02	5.97E+02	6.23E+02	5.42E+02	6.14E+02
	Std	2.91E+01	3.63E+01	3.30E+01	1.01E+01	2.64E+01
F5	Best	6.01E+02	6.00E+02	6.00E+02	6.01E+02	6.00E+02
	Mean	6.05E+02	6.00E+02	6.00E+02	6.03E+02	6.00E+02
	Std	3.48E+00	2.28E-02	4.66E-03	7.16E-01	3.60E-03
F6	Best	8.05E+02	7.74E+02	7.83E+02	7.51E+02	7.77E+02
	Mean	8.48E+02	8.53E+02	8.65E+02	7.77E+02	8.64E+02
	Std	2.84E+01	3.18E+01	3.72E+01	1.31E+01	3.78E+01
F7	Best	8.62E+02	8.39E+02	8.59E+02	8.24E+02	8.59E+02
	Mean	9.08E+02	9.05E+02	9.05E+02	8.46E+02	9.04E+02
	Std	2.83E+01	3.22E+01	2.35E+01	1.24E+01	2.27E+01
F8	Best	1.26E+03	9.08E+02	1.01E+03	9.03E+02	9.02E+02
	Mean	3.56E+03	9.86E+02	1.90E+03	9.17E+02	1.05E+03
	Std	1.46E+03	7.90E+01	6.19E+02	1.37E+01	1.53E+02
F9	Best	3.23E+03	2.53E+03	2.86E+03	2.57E+03	2.30E+03
	Mean	4.59E+03	4.05E+03	4.02E+03	4.15E+03	3.99E+03
	Std	6.33E+02	5.97E+02	5.20E+02	6.24E+02	5.70E+02
F10	Best	1.15E+03	1.12E+03	1.12E+03	1.15E+03	1.12E+03
	Mean	1.26E+03	1.16E+03	1.16E+03	1.23E+03	1.16E+03
	Std	5.43E+01	3.11E+01	2.34E+01	4.26E+01	3.07E+01
F11	Best	3.92E+05	6.06E+03	1.47E+04	1.11E+04	7.96E+03
	Mean	2.28E+06	9.58E+04	6.95E+04	2.47E+05	6.62E+04
	Std	1.68E+06	1.54E+05	3.70E+04	2.12E+05	5.04E+04
F12	Best	7.65E+03	1.49E+03	1.35E+03	1.45E+03	1.33E+03
	Mean	3.24E+04	8.56E+03	7.93E+03	4.71E+03	1.45E+03
	Std	2.51E+04	1.77E+04	1.07E+04	9.70E+03	1.30E+02
F13	Best	1.85E+03	1.43E+03	1.44E+03	1.44E+03	1.42E+03
	Mean	7.55E+04	1.46E+03	1.48E+03	1.47E+03	1.44E+03
	Std	4.72E+04	1.67E+01	3.30E+01	2.01E+01	1.20E+01
F14	Best	1.87E+03	1.53E+03	1.51E+03	1.55E+03	1.51E+03
	Mean	2.71E+04	3.56E+03	1.60E+03	1.65E+03	1.54E+03
	Std	1.51E+04	8.00E+03	1.84E+02	5.22E+01	2.47E+01
F15	Best	1.86E+03	1.96E+03	1.92E+03	1.63E+03	1.61E+03
	Mean	2.56E+03	2.33E+03	2.43E+03	1.99E+03	2.25E+03
	Std	2.94E+02	2.32E+02	3.06E+02	2.16E+02	3.03E+02
F16	Best	1.92E+03	1.75E+03	1.74E+03	1.74E+03	1.72E+03
	Mean	2.29E+03	1.95E+03	1.99E+03	1.82E+03	1.83E+03
	Std	2.08E+02	1.86E+02	2.00E+02	8.91E+01	8.18E+01
F17	Best	1.21E+05	1.85E+03	2.73E+03	1.89E+03	1.83E+03
	Mean	1.08E+06	1.91E+03	2.59E+04	4.92E+03	6.42E+03
	Std	9.27E+05	3.13E+01	1.78E+04	4.34E+03	5.52E+03
F18	Best	2.08E+03	1.91E+03	1.91E+03	1.92E+03	1.91E+03
	Mean	3.26E+04	3.93E+03	2.09E+03	1.95E+03	1.92E+03
	Std	2.20E+04	8.36E+03	6.14E+02	1.93E+01	7.12E+00
F19	Best	2.08E+03	2.04E+03	2.04E+03	2.03E+03	2.03E+03
	Mean	2.52E+03	2.32E+03	2.35E+03	2.17E+03	2.20E+03
	Std	1.86E+02	1.82E+02	2.07E+02	1.48E+02	1.37E+02
F20	Best	2.35E+03	2.32E+03	2.35E+03	2.33E+03	2.35E+03
	Mean	2.41E+03	2.40E+03	2.41E+03	2.34E+03	2.40E+03
	Std	2.82E+01	3.40E+01	3.19E+01	9.61E+00	2.36E+01
F21	Best	2.30E+03	2.30E+03	2.30E+03	2.30E+03	2.30E+03
	Mean	5.78E+03	3.21E+03	4.76E+03	3.42E+03	3.95E+03
	Std	8.18E+02	1.50E+03	1.61E+03	1.57E+03	1.80E+03
F22	Best	2.71E+03	2.68E+03	2.72E+03	2.67E+03	2.69E+03
	Mean	2.76E+03	2.77E+03	2.78E+03	2.69E+03	2.76E+03
	Std	2.81E+01	2.87E+01	3.31E+01	1.25E+01	3.27E+01
F23	Best	2.87E+03	2.86E+03	2.87E+03	2.83E+03	2.86E+03
	Mean	2.93E+03	2.93E+03	2.97E+03	2.86E+03	2.91E+03
	Std	3.12E+01	3.81E+01	5.07E+01	1.26E+01	3.23E+01
F24	Best	2.88E+03	2.88E+03	2.88E+03	2.88E+03	2.88E+03
	Mean	2.89E+03	2.89E+03	2.90E+03	2.90E+03	2.89E+03
	Std	1.75E+01	3.54E+00	1.67E+01	1.88E+01	9.62E+00
F25	Best	4.23E+03	2.80E+03	2.80E+03	2.81E+03	2.80E+03
	Mean	4.82E+03	4.62E+03	4.41E+03	4.03E+03	4.28E+03
	Std	2.95E+02	6.27E+02	1.21E+03	2.21E+02	8.81E+02
F26	Best	3.20E+03	3.18E+03	3.20E+03	3.18E+03	3.19E+03
	Mean	3.22E+03	3.21E+03	3.23E+03	3.22E+03	3.22E+03
	Std	1.63E+01	1.25E+01	1.68E+01	1.21E+01	1.33E+01
F27	Best	3.20E+03	3.10E+03	3.10E+03	3.20E+03	3.10E+03
	Mean	3.25E+03	3.15E+03	3.13E+03	3.24E+03	3.13E+03
	Std	4.23E+01	5.09E+01	5.09E+01	2.45E+01	5.31E+01
F28	Best	3.45E+03	3.32E+03	3.37E+03	3.35E+03	3.28E+03
	Mean	3.83E+03	3.59E+03	3.63E+03	3.50E+03	3.47E+03
	Std	2.07E+02	1.70E+02	1.55E+02	9.03E+01	1.19E+02
F29	Best	9.77E+03	5.72E+03	5.36E+03	5.69E+03	5.03E+03
	Mean	2.75E+04	1.06E+04	9.35E+03	1.05E+04	7.46E+03
	Std	1.13E+04	5.60E+03	4.16E+03	4.42E+03	5.81E+03

**Table A3:** Statistical results obtained from EMSMA and EMSMA variants for CEC 2017 (Dim = 50).

Function	Index	SMA	EMSMA-1	EMSMA-2	EMSMA-3	EMSMA
F1	Best	6.43E+04	1.33E+02	1.48E+02	2.38E+05	1.08E+02
	Mean	1.65E+05	4.32E+03	3.90E+03	1.05E+06	3.37E+03
	Std	5.83E+04	5.43E+03	3.95E+03	6.08E+05	3.52E+03
F2	Best	1.20E+04	4.43E+02	4.71E+02	4.22E+02	3.66E+02
	Mean	3.23E+04	1.26E+03	1.89E+03	1.61E+03	7.04E+02
	Std	1.33E+04	5.72E+02	1.10E+03	1.21E+03	4.08E+02
F3	Best	4.72E+02	4.00E+02	4.00E+02	4.70E+02	4.00E+02
	Mean	5.89E+02	4.95E+02	4.50E+02	5.90E+02	4.55E+02
	Std	3.83E+01	4.92E+01	3.78E+01	4.98E+01	4.50E+01
F4	Best	6.76E+02	5.95E+02	6.62E+02	5.79E+02	6.67E+02
	Mean	7.59E+02	7.56E+02	7.30E+02	6.13E+02	7.42E+02
	Std	4.52E+01	7.02E+01	4.24E+01	2.04E+01	3.96E+01
F5	Best	6.12E+02	6.00E+02	6.00E+02	6.04E+02	6.00E+02
	Mean	6.33E+02	6.00E+02	6.00E+02	6.06E+02	6.00E+02
	Std	1.27E+01	5.98E-03	5.82E-03	1.19E+00	5.58E-03
F6	Best	9.59E+02	8.90E+02	8.98E+02	8.30E+02	8.92E+02
	Mean	1.03E+03	1.02E+03	1.00E+03	8.76E+02	1.01E+03
	Std	4.49E+01	5.99E+01	5.92E+01	2.72E+01	5.16E+01
F7	Best	9.43E+02	8.84E+02	9.37E+02	8.78E+02	9.50E+02
	Mean	1.04E+03	1.03E+03	1.04E+03	9.14E+02	1.03E+03
	Std	4.82E+01	6.68E+01	4.80E+01	2.53E+01	4.43E+01
F8	Best	4.96E+03	1.05E+03	2.07E+03	9.52E+02	1.45E+03
	Mean	1.20E+04	2.06E+03	5.72E+03	1.24E+03	3.71E+03
	Std	3.71E+03	6.47E+02	2.14E+03	2.88E+02	1.67E+03
F9	Best	5.31E+03	4.32E+03	4.75E+03	4.95E+03	4.45E+03
	Mean	7.08E+03	6.12E+03	6.13E+03	7.45E+03	5.99E+03
	Std	8.11E+02	7.22E+02	5.25E+02	1.03E+03	7.57E+02
F10	Best	1.23E+03	1.16E+03	1.17E+03	1.29E+03	1.17E+03
	Mean	1.40E+03	1.23E+03	1.21E+03	1.42E+03	1.21E+03
	Std	8.34E+01	3.25E+01	2.98E+01	8.33E+01	2.97E+01
F11	Best	2.17E+06	3.76E+05	7.99E+04	6.85E+05	1.32E+05
	Mean	1.73E+07	2.26E+06	9.85E+05	6.49E+06	8.01E+05
	Std	8.46E+06	1.39E+06	7.05E+05	4.31E+06	5.51E+05
F12	Best	4.28E+04	1.44E+03	1.44E+03	3.58E+03	1.45E+03
	Mean	7.08E+04	1.47E+04	8.45E+03	1.15E+04	5.50E+03
	Std	1.78E+04	1.25E+04	8.18E+03	7.48E+03	4.82E+03
F13	Best	9.21E+04	1.48E+03	1.54E+03	1.51E+03	1.46E+03
	Mean	4.00E+05	1.57E+03	3.82E+03	1.63E+03	1.52E+03
	Std	2.12E+05	4.21E+01	5.22E+03	7.13E+01	3.11E+01
F14	Best	4.62E+03	1.83E+03	1.55E+03	1.91E+03	1.54E+03
	Mean	2.71E+04	7.90E+03	3.44E+03	2.65E+03	1.63E+03
	Std	1.12E+04	8.50E+03	4.29E+03	1.37E+03	1.07E+02
F15	Best	2.58E+03	2.38E+03	2.54E+03	1.94E+03	2.08E+03
	Mean	3.40E+03	3.40E+03	3.41E+03	2.56E+03	2.99E+03
	Std	3.44E+02	4.64E+02	4.49E+02	3.03E+02	4.76E+02
F16	Best	2.41E+03	2.14E+03	2.15E+03	1.91E+03	2.03E+03
	Mean	3.31E+03	2.79E+03	2.90E+03	2.46E+03	2.60E+03
	Std	3.30E+02	3.38E+02	3.25E+02	2.39E+02	3.09E+02
F17	Best	3.30E+05	2.60E+03	2.60E+04	9.67E+03	6.42E+03
	Mean	3.24E+06	5.28E+03	8.02E+04	4.46E+04	3.14E+04
	Std	2.11E+06	2.50E+03	4.28E+04	4.38E+04	1.87E+04
F18	Best	2.14E+03	1.99E+03	1.93E+03	2.00E+03	1.92E+03
	Mean	1.61E+04	1.70E+04	2.96E+03	2.48E+03	1.95E+03
	Std	1.74E+04	1.56E+04	4.92E+03	1.27E+03	2.36E+01
F19	Best	2.58E+03	2.15E+03	2.25E+03	2.16E+03	2.25E+03
	Mean	3.25E+03	2.96E+03	3.05E+03	2.58E+03	2.77E+03
	Std	2.96E+02	2.96E+02	3.26E+02	2.16E+02	2.48E+02
F20	Best	2.46E+03	2.37E+03	2.46E+03	2.37E+03	2.45E+03
	Mean	2.53E+03	2.52E+03	2.54E+03	2.41E+03	2.52E+03
	Std	4.69E+01	4.62E+01	4.93E+01	1.90E+01	3.79E+01
F21	Best	7.18E+03	6.59E+03	6.55E+03	6.93E+03	6.46E+03
	Mean	9.05E+03	8.13E+03	8.03E+03	8.95E+03	8.34E+03
	Std	9.69E+02	7.82E+02	7.57E+02	9.99E+02	7.90E+02
F22	Best	2.87E+03	2.85E+03	2.88E+03	2.77E+03	2.90E+03
	Mean	2.97E+03	2.99E+03	2.99E+03	2.82E+03	3.00E+03
	Std	4.62E+01	5.45E+01	5.35E+01	2.25E+01	4.69E+01
F23	Best	3.06E+03	2.96E+03	3.15E+03	2.95E+03	2.99E+03
	Mean	3.15E+03	3.07E+03	3.30E+03	2.98E+03	3.13E+03
	Std	5.73E+01	8.89E+01	1.16E+02	2.22E+01	8.68E+01
F24	Best	2.96E+03	2.96E+03	2.96E+03	2.99E+03	2.93E+03
	Mean	3.06E+03	3.06E+03	3.04E+03	3.09E+03	3.03E+03
	Std	3.26E+01	3.60E+01	4.44E+01	3.08E+01	4.08E+01
F25	Best	2.90E+03	2.90E+03	2.90E+03	2.92E+03	2.90E+03
	Mean	5.68E+03	5.02E+03	6.68E+03	4.83E+03	5.79E+03
	Std	1.76E+03	1.56E+03	1.93E+03	4.49E+02	1.95E+03
F26	Best	3.30E+03	3.24E+03	3.29E+03	3.25E+03	3.25E+03
	Mean	3.44E+03	3.36E+03	3.47E+03	3.40E+03	3.40E+03
	Std	8.23E+01	7.93E+01	8.47E+01	8.47E+01	7.71E+01
F27	Best	3.28E+03	3.26E+03	3.25E+03	3.28E+03	3.26E+03
	Mean	3.35E+03	3.30E+03	3.30E+03	3.35E+03	3.29E+03
	Std	3.55E+01	2.50E+01	4.42E+01	3.41E+01	3.41E+01
F28	Best	3.94E+03	3.35E+03	3.55E+03	3.42E+03	3.34E+03
	Mean	4.69E+03	4.02E+03	4.13E+03	3.85E+03	3.82E+03
	Std	3.38E+02	3.69E+02	3.75E+02	2.19E+02	2.67E+02
F29	Best	1.73E+06	5.93E+05	5.83E+05	9.65E+05	5.82E+05
	Mean	3.05E+06	8.88E+05	6.89E+05	1.69E+06	6.25E+05
	Std	6.71E+05	2.64E+05	1.09E+05	4.32E+05	5.84E+04

**Table A4:** Statistical results obtained from EMSMA and EMSMA variants for CEC 2017 (Dim = 100).

Function	Index	SMA	EMSMA-1	EMSMA-2	EMSMA-3	EMSMA
F1	Best	9.78E+06	1.56E+02	1.95E+03	1.42E+07	1.51E+03
	Mean	1.86E+07	7.31E+03	8.09E+03	3.47E+07	7.32E+03
	Std	4.56E+06	5.72E+03	5.88E+03	1.32E+07	5.98E+03
F2	Best	1.87E+05	4.39E+04	3.48E+04	5.21E+04	2.75E+04
	Mean	4.03E+05	7.73E+04	6.56E+04	7.24E+04	4.54E+04
	Std	1.39E+05	1.49E+04	1.25E+04	1.32E+04	9.35E+03
F3	Best	6.73E+02	5.02E+02	4.12E+02	7.03E+02	4.73E+02
	Mean	7.72E+02	6.27E+02	5.71E+02	8.15E+02	5.55E+02
	Std	6.34E+01	4.61E+01	6.22E+01	5.31E+01	5.89E+01
F4	Best	1.01E+03	1.04E+03	9.43E+02	7.78E+02	9.63E+02
	Mean	1.21E+03	1.23E+03	1.11E+03	8.97E+02	1.12E+03
	Std	8.82E+01	9.93E+01	7.69E+01	5.23E+01	8.96E+01
F5	Best	6.32E+02	6.00E+02	6.00E+02	6.09E+02	6.00E+02
	Mean	6.49E+02	6.00E+02	6.00E+02	6.14E+02	6.00E+02
	Std	8.37E+00	4.13E-03	3.50E-03	2.97E+00	4.38E-03
F6	Best	1.63E+03	1.25E+03	1.39E+03	1.15E+03	1.25E+03
	Mean	1.91E+03	1.68E+03	1.60E+03	1.28E+03	1.59E+03
	Std	1.63E+02	1.88E+02	1.11E+02	5.97E+01	1.53E+02
F7	Best	1.29E+03	1.04E+03	1.29E+03	1.07E+03	1.28E+03
	Mean	1.52E+03	1.45E+03	1.44E+03	1.18E+03	1.44E+03
	Std	1.20E+02	1.76E+02	6.64E+01	5.46E+01	9.06E+01
F8	Best	1.94E+04	6.67E+03	1.12E+04	5.02E+03	1.23E+04
	Mean	3.00E+04	1.97E+04	1.85E+04	1.06E+04	1.81E+04
	Std	4.33E+03	5.15E+03	3.40E+03	3.30E+03	3.08E+03
F9	Best	1.28E+04	1.06E+04	1.08E+04	1.40E+04	1.02E+04
	Mean	1.61E+04	1.32E+04	1.32E+04	1.86E+04	1.29E+04
	Std	1.47E+03	1.17E+03	1.12E+03	2.33E+03	1.11E+03
F10	Best	2.30E+03	1.31E+03	1.37E+03	2.36E+03	1.29E+03
	Mean	3.23E+03	1.53E+03	1.50E+03	2.88E+03	1.49E+03
	Std	7.22E+02	9.51E+01	6.64E+01	3.10E+02	9.14E+01
F11	Best	3.52E+07	9.23E+05	7.44E+05	1.60E+07	8.56E+05
	Mean	1.35E+08	3.97E+06	1.99E+06	5.53E+07	1.97E+06
	Std	5.93E+07	2.04E+06	8.44E+05	2.82E+07	8.77E+05
F12	Best	4.78E+04	1.64E+03	1.68E+03	2.19E+04	1.67E+03
	Mean	4.91E+05	6.65E+03	5.61E+03	6.43E+04	5.15E+03
	Std	8.28E+05	7.07E+03	4.39E+03	2.56E+04	4.31E+03
F13	Best	4.03E+05	3.56E+03	1.26E+04	4.38E+03	3.39E+03
	Mean	2.60E+06	9.02E+03	1.14E+05	5.82E+04	4.03E+04
	Std	1.44E+06	4.50E+03	8.17E+04	6.53E+04	3.50E+04
F14	Best	2.45E+04	1.70E+03	1.70E+03	4.49E+03	1.62E+03
	Mean	1.70E+05	4.61E+03	3.55E+03	1.27E+04	4.04E+03
	Std	1.86E+05	4.87E+03	2.09E+03	6.21E+03	3.44E+03
F15	Best	4.98E+03	4.30E+03	3.53E+03	3.34E+03	3.75E+03
	Mean	5.99E+03	6.05E+03	6.01E+03	4.68E+03	5.84E+03
	Std	5.41E+02	7.88E+02	7.20E+02	7.09E+02	8.07E+02
F16	Best	4.66E+03	3.68E+03	3.39E+03	3.35E+03	3.53E+03
	Mean	5.67E+03	4.96E+03	4.89E+03	4.17E+03	4.78E+03
	Std	5.50E+02	5.92E+02	5.24E+02	3.70E+02	6.67E+02
F17	Best	2.09E+06	5.26E+04	3.86E+04	6.80E+04	4.63E+04
	Mean	5.76E+06	1.09E+05	2.54E+05	2.24E+05	1.32E+05
	Std	2.74E+06	3.37E+04	1.06E+05	1.21E+05	5.26E+04
F18	Best	9.42E+03	2.02E+03	2.02E+03	2.66E+03	1.98E+03
	Mean	7.10E+04	5.47E+03	5.62E+03	4.43E+04	4.24E+03
	Std	6.04E+04	5.72E+03	5.81E+03	5.42E+04	3.19E+03
F19	Best	4.53E+03	3.72E+03	3.78E+03	3.16E+03	3.93E+03
	Mean	5.36E+03	5.02E+03	5.12E+03	4.17E+03	5.05E+03
	Std	4.59E+02	5.08E+02	5.60E+02	4.86E+02	5.74E+02
F20	Best	2.81E+03	2.76E+03	2.72E+03	2.57E+03	2.75E+03
	Mean	3.01E+03	2.92E+03	2.90E+03	2.66E+03	2.89E+03
	Std	9.17E+01	8.10E+01	8.59E+01	4.21E+01	7.39E+01
F21	Best	1.50E+04	1.20E+04	1.35E+04	1.42E+04	1.32E+04
	Mean	1.86E+04	1.60E+04	1.59E+04	2.03E+04	1.60E+04
	Std	1.44E+03	1.54E+03	1.37E+03	2.37E+03	1.31E+03
F22	Best	3.20E+03	3.07E+03	3.06E+03	3.04E+03	3.05E+03
	Mean	3.36E+03	3.18E+03	3.18E+03	3.12E+03	3.18E+03
	Std	7.62E+01	4.96E+01	5.78E+01	4.46E+01	5.73E+01
F23	Best	3.78E+03	3.59E+03	3.68E+03	3.50E+03	3.67E+03
	Mean	3.97E+03	3.83E+03	3.86E+03	3.59E+03	3.85E+03
	Std	1.09E+02	8.38E+01	9.54E+01	4.96E+01	8.22E+01
F24	Best	3.35E+03	3.18E+03	3.11E+03	3.31E+03	3.13E+03
	Mean	3.45E+03	3.30E+03	3.27E+03	3.47E+03	3.26E+03
	Std	5.35E+01	5.03E+01	6.10E+01	7.43E+01	6.02E+01
F25	Best	3.13E+03	2.90E+03	2.90E+03	3.92E+03	2.90E+03
	Mean	1.31E+04	1.13E+04	1.47E+04	9.21E+03	1.37E+04
	Std	1.76E+03	2.95E+03	4.04E+03	9.90E+02	3.97E+03
F26	Best	3.45E+03	3.44E+03	3.44E+03	3.41E+03	3.41E+03
	Mean	3.62E+03	3.56E+03	3.56E+03	3.58E+03	3.54E+03
	Std	9.14E+01	7.41E+01	7.65E+01	9.78E+01	8.50E+01
F27	Best	3.46E+03	3.33E+03	3.27E+03	3.43E+03	3.30E+03
	Mean	3.53E+03	3.39E+03	3.35E+03	3.57E+03	3.36E+03
	Std	3.99E+01	3.09E+01	3.78E+01	5.83E+01	2.81E+01
F28	Best	5.95E+03	5.79E+03	5.80E+03	5.35E+03	5.68E+03
	Mean	7.33E+03	7.17E+03	6.93E+03	6.21E+03	6.85E+03
	Std	5.68E+02	6.20E+02	5.71E+02	5.30E+02	5.23E+02
F29	Best	4.15E+05	6.74E+03	6.36E+03	8.42E+05	6.23E+03
	Mean	1.58E+06	1.63E+04	1.36E+04	2.73E+06	1.30E+04
	Std	9.09E+05	5.55E+03	5.52E+03	1.63E+06	5.24E+03

**Table A5:** Statistical results obtained from EMSMA and EMSMA variants for CEC 2022 (Dim = 10).

Function	Index	SMA	EMSMA-1	EMSMA-2	EMSMA-3	EMSMA
F1	Best	3.00E+02	3.00E+02	3.00E+02	3.00E+02	3.00E+02
	Mean	3.00E+02	3.00E+02	3.00E+02	3.00E+02	3.00E+02
	Std	1.14E-03	1.99E-06	1.58E-01	6.20E-02	4.09E-07
F2	Best	4.01E+02	4.00E+02	4.00E+02	4.00E+02	4.00E+02
	Mean	4.12E+02	4.06E+02	4.05E+02	4.05E+02	4.07E+02
	Std	1.66E+01	2.99E+00	3.60E+00	3.35E+00	9.70E+00
F3	Best	6.00E+02	6.00E+02	6.00E+02	6.00E+02	6.00E+02
	Mean	6.00E+02	6.00E+02	6.00E+02	6.00E+02	6.00E+02
	Std	3.76E-02	6.03E-03	8.39E-03	1.72E-02	2.82E-03
F4	Best	8.09E+02	8.04E+02	8.06E+02	8.03E+02	8.03E+02
	Mean	8.25E+02	8.13E+02	8.16E+02	8.07E+02	8.09E+02
	Std	9.75E+00	5.88E+00	7.06E+00	2.53E+00	3.86E+00
F5	Best	9.00E+02	9.00E+02	9.00E+02	9.00E+02	9.00E+02
	Mean	9.01E+02	9.00E+02	9.00E+02	9.00E+02	9.00E+02
	Std	1.99E+00	5.01E-06	3.50E-01	2.18E-01	1.36E-01
F6	Best	1.92E+03	1.80E+03	1.80E+03	1.80E+03	1.80E+03
	Mean	5.50E+03	1.80E+03	2.24E+03	1.80E+03	1.80E+03
	Std	2.35E+03	3.88E-01	8.38E+02	4.51E+00	1.24E+01
F7	Best	2.02E+03	2.00E+03	2.00E+03	2.00E+03	2.00E+03
	Mean	2.02E+03	2.00E+03	2.01E+03	2.01E+03	2.00E+03
	Std	7.79E-01	7.88E+00	9.53E+00	8.52E+00	8.16E+00
F8	Best	2.20E+03	2.20E+03	2.20E+03	2.20E+03	2.20E+03
	Mean	2.22E+03	2.21E+03	2.22E+03	2.20E+03	2.21E+03
	Std	2.79E+00	1.00E+01	7.54E+00	8.13E+00	8.79E+00
F9	Best	2.53E+03	2.53E+03	2.53E+03	2.53E+03	2.53E+03
	Mean	2.53E+03	2.53E+03	2.53E+03	2.53E+03	2.53E+03
	Std	3.28E-04	3.39E-08	2.06E+01	2.41E-05	1.71E-11
F10	Best	2.50E+03	2.50E+03	2.50E+03	2.50E+03	2.50E+03
	Mean	2.52E+03	2.51E+03	2.53E+03	2.50E+03	2.52E+03
	Std	4.24E+01	2.74E+01	5.17E+01	2.23E+01	4.23E+01
F11	Best	2.60E+03	2.60E+03	2.60E+03	2.60E+03	2.60E+03
	Mean	2.79E+03	2.65E+03	2.83E+03	2.67E+03	2.79E+03
	Std	1.84E+02	1.10E+02	1.26E+02	1.23E+02	1.46E+02
F12	Best	2.86E+03	2.86E+03	2.86E+03	2.86E+03	2.86E+03
	Mean	2.86E+03	2.86E+03	2.86E+03	2.86E+03	2.86E+03
	Std	1.50E+00	1.23E+00	1.38E+00	1.63E+00	1.69E+00

**Table A6:** Statistical results obtained from EMSMA and EMSMA variants for CEC 2022 (Dim = 20).

Function	Index	SMA	EMSMA-1	EMSMA-2	EMSMA-3	EMSMA
F1	Best	3.00E+02	3.00E+02	3.00E+02	3.01E+02	3.00E+02
	Mean	3.00E+02	3.00E+02	3.02E+02	3.04E+02	3.00E+02
	Std	2.71E-01	3.45E-02	1.75E+00	3.04E+00	6.50E-01
F2	Best	4.05E+02	4.00E+02	4.00E+02	4.05E+02	4.00E+02
	Mean	4.48E+02	4.44E+02	4.39E+02	4.54E+02	4.36E+02
	Std	1.60E+01	1.70E+01	2.11E+01	1.33E+01	2.19E+01
F3	Best	6.00E+02	6.00E+02	6.00E+02	6.00E+02	6.00E+02
	Mean	6.01E+02	6.00E+02	6.00E+02	6.01E+02	6.00E+02
	Std	1.14E+00	8.39E-03	5.71E-03	3.34E-01	3.99E-03
F4	Best	8.21E+02	8.09E+02	8.33E+02	8.07E+02	8.16E+02
	Mean	8.67E+02	8.46E+02	8.72E+02	8.20E+02	8.48E+02
	Std	2.38E+01	2.48E+01	1.90E+01	6.15E+00	1.87E+01
F5	Best	9.11E+02	9.00E+02	9.00E+02	9.00E+02	9.00E+02
	Mean	1.53E+03	9.03E+02	1.26E+03	9.03E+02	9.04E+02
	Std	6.49E+02	5.05E+00	4.99E+02	2.73E+00	4.16E+00
F6	Best	2.02E+03	1.83E+03	1.83E+03	1.81E+03	1.81E+03
	Mean	2.02E+04	1.86E+03	5.38E+03	2.20E+03	1.90E+03
	Std	6.72E+03	2.76E+01	5.04E+03	1.00E+03	1.32E+02
F7	Best	2.03E+03	2.01E+03	2.02E+03	2.02E+03	2.01E+03
	Mean	2.07E+03	2.04E+03	2.06E+03	2.03E+03	2.03E+03
	Std	4.02E+01	3.28E+01	3.03E+01	5.15E+00	1.85E+01
F8	Best	2.22E+03	2.22E+03	2.22E+03	2.20E+03	2.21E+03
	Mean	2.25E+03	2.22E+03	2.23E+03	2.22E+03	2.22E+03
	Std	4.95E+01	5.28E-01	2.88E+01	4.65E+00	1.86E+00
F9	Best	2.48E+03	2.48E+03	2.48E+03	2.48E+03	2.48E+03
	Mean	2.48E+03	2.48E+03	2.48E+03	2.48E+03	2.48E+03
	Std	1.06E-01	9.19E-04	2.13E-02	1.76E-01	9.87E-04
F10	Best	2.50E+03	2.40E+03	2.40E+03	2.50E+03	2.40E+03
	Mean	2.88E+03	2.46E+03	2.45E+03	2.61E+03	2.47E+03
	Std	2.79E+02	4.98E+01	4.97E+01	2.32E+02	5.87E+01
F11	Best	2.60E+03	2.60E+03	2.60E+03	2.90E+03	2.90E+03
	Mean	2.95E+03	2.92E+03	2.89E+03	2.93E+03	2.90E+03
	Std	1.46E+02	6.11E+01	4.20E+01	7.64E+01	5.39E+00
F12	Best	2.93E+03	2.93E+03	2.93E+03	2.93E+03	2.93E+03
	Mean	2.95E+03	2.94E+03	2.95E+03	2.94E+03	2.95E+03
	Std	1.01E+01	9.30E+00	1.59E+01	6.11E+00	1.11E+01

**Table A7:** Statistical results derived from EMSMA and SMA variants for CEC 2017 (Dim = 10).

Function	Index	EMSMA	SMA	DTSMA	ISMA	AOSMA	LSMA	ESMA	MSMA
F1	Best	1.00E+02	1.55E+03	1.15E+02	1.02E+02	1.01E+02	1.07E+02	1.04E+02	1.00E+02
	Mean	2.13E+03	8.60E+03	6.19E+03	3.90E+03	7.35E+03	7.46E+03	6.96E+03	6.59E+03
	Std	2.56E+03	3.92E+03	4.12E+03	3.23E+03	4.56E+03	4.37E+03	4.22E+03	4.60E+03
F2	Best	3.00E+02	3.00E+02	3.00E+02	3.00E+02	3.00E+02	3.00E+02	3.00E+02	3.00E+02
	Mean	3.00E+02	3.00E+02	3.00E+02	3.00E+02	3.00E+02	3.00E+02	3.00E+02	3.00E+02
	Std	3.92E-07	3.36E-03	2.88E-05	8.70E-02	2.15E-01	8.60E-04	8.20E-04	2.38E-04
F3	Best	4.00E+02	4.01E+02	4.00E+02	4.00E+02	4.01E+02	4.00E+02	4.01E+02	4.00E+02
	Mean	4.00E+02	4.19E+02	4.03E+02	4.07E+02	4.14E+02	4.19E+02	4.16E+02	4.11E+02
	Std	8.39E-02	2.75E+01	7.55E-01	1.17E+01	2.28E+01	2.67E+01	2.55E+01	1.72E+01
F4	Best	5.01E+02	5.05E+02	5.02E+02	5.11E+02	5.11E+02	5.08E+02	5.04E+02	5.05E+02
	Mean	5.08E+02	5.16E+02	5.13E+02	5.31E+02	5.28E+02	5.21E+02	5.17E+02	5.18E+02
	Std	3.25E+00	7.28E+00	5.89E+00	1.06E+01	1.10E+01	5.47E+00	5.90E+00	7.37E+00
F5	Best	6.00E+02	6.00E+02	6.00E+02	6.00E+02	6.00E+02	6.00E+02	6.00E+02	6.00E+02
	Mean	6.00E+02	6.00E+02	6.00E+02	6.05E+02	6.03E+02	6.00E+02	6.00E+02	6.00E+02
	Std	1.04E-03	4.45E-02	5.36E-02	4.64E+00	4.37E+00	4.18E-01	2.01E-02	5.71E-01
F6	Best	7.11E+02	7.11E+02	7.10E+02	7.19E+02	7.17E+02	7.11E+02	7.12E+02	7.16E+02
	Mean	7.20E+02	7.24E+02	7.24E+02	7.53E+02	7.44E+02	7.26E+02	7.24E+02	7.37E+02
	Std	4.57E+00	6.18E+00	6.42E+00	2.00E+01	1.48E+01	6.64E+00	5.83E+00	1.16E+01
F7	Best	8.03E+02	8.07E+02	8.03E+02	8.08E+02	8.08E+02	8.07E+02	8.05E+02	8.05E+02
	Mean	8.09E+02	8.16E+02	8.15E+02	8.26E+02	8.25E+02	8.19E+02	8.15E+02	8.20E+02
	Std	3.07E+00	6.80E+00	6.77E+00	8.49E+00	8.70E+00	8.23E+00	5.46E+00	7.96E+00
F8	Best	9.00E+02	9.00E+02	9.00E+02	9.00E+02	9.00E+02	9.00E+02	9.00E+02	9.00E+02
	Mean	9.00E+02	9.00E+02	9.00E+02	9.59E+02	9.84E+02	9.00E+02	9.00E+02	9.04E+02
	Std	1.09E-01	2.43E-02	1.23E-01	8.95E+01	1.49E+02	1.30E-01	2.67E-04	1.14E+01
F9	Best	1.02E+03	1.16E+03	1.15E+03	1.26E+03	1.19E+03	1.15E+03	1.14E+03	1.01E+03
	Mean	1.37E+03	1.60E+03	1.40E+03	1.75E+03	1.68E+03	1.64E+03	1.56E+03	1.50E+03
	Std	1.55E+02	2.49E+02	1.13E+02	2.73E+02	2.92E+02	2.29E+02	2.42E+02	2.18E+02
F10	Best	1.10E+03	1.10E+03	1.10E+03	1.11E+03	1.10E+03	1.11E+03	1.10E+03	1.10E+03
	Mean	1.10E+03	1.17E+03	1.11E+03	1.15E+03	1.19E+03	1.22E+03	1.11E+03	1.11E+03
	Std	1.91E+00	7.47E+01	6.32E+00	4.69E+01	7.92E+01	7.59E+01	1.92E+01	5.37E+00
F11	Best	1.20E+03	2.79E+03	1.72E+03	5.27E+03	5.65E+03	6.95E+03	2.39E+03	2.18E+03
	Mean	2.40E+03	4.00E+04	2.19E+04	4.59E+05	4.41E+05	2.68E+05	4.37E+04	5.71E+04
	Std	2.33E+03	2.64E+04	2.05E+04	6.70E+05	5.56E+05	3.37E+05	5.09E+04	6.29E+04
F12	Best	1.30E+03	1.39E+03	1.33E+03	1.56E+03	2.07E+03	1.73E+03	1.33E+03	1.32E+03
	Mean	1.31E+03	1.21E+04	1.08E+04	1.58E+04	1.42E+04	1.21E+04	1.10E+04	6.58E+03
	Std	3.48E+00	1.28E+04	1.11E+04	1.02E+04	1.22E+04	1.17E+04	1.21E+04	4.39E+03
F13	Best	1.40E+03	1.43E+03	1.40E+03	1.43E+03	1.47E+03	1.43E+03	1.41E+03	1.41E+03
	Mean	1.40E+03	1.46E+03	1.44E+03	1.49E+03	1.72E+03	1.50E+03	1.91E+03	1.43E+03
	Std	1.30E+00	8.22E+01	1.13E+02	3.36E+01	7.08E+02	4.33E+01	1.41E+03	1.65E+01
F14	Best	1.50E+03	1.51E+03	1.50E+03	1.54E+03	1.59E+03	1.56E+03	1.52E+03	1.50E+03
	Mean	1.50E+03	2.11E+03	1.55E+03	1.79E+03	2.86E+03	1.96E+03	4.82E+03	1.78E+03
	Std	8.30E-01	1.06E+03	9.07E+01	4.66E+02	1.60E+03	7.65E+02	2.93E+03	7.02E+02
F15	Best	1.60E+03	1.60E+03	1.60E+03	1.60E+03	1.61E+03	1.60E+03	1.60E+03	1.60E+03
	Mean	1.62E+03	1.69E+03	1.65E+03	1.72E+03	1.73E+03	1.67E+03	1.72E+03	1.73E+03
	Std	3.84E+01	8.85E+01	5.11E+01	1.03E+02	1.10E+02	8.12E+01	9.74E+01	1.30E+02
F16	Best	1.70E+03	1.70E+03	1.70E+03	1.72E+03	1.72E+03	1.71E+03	1.70E+03	1.71E+03
	Mean	1.71E+03	1.75E+03	1.73E+03	1.75E+03	1.76E+03	1.75E+03	1.74E+03	1.74E+03
	Std	8.05E+00	3.81E+01	1.15E+01	1.67E+01	3.07E+01	3.16E+01	1.97E+01	3.25E+01
F17	Best	1.80E+03	2.50E+03	2.09E+03	2.02E+03	6.55E+03	2.87E+03	2.02E+03	1.98E+03
	Mean	1.80E+03	2.60E+04	2.23E+04	1.94E+04	2.81E+04	2.80E+04	2.27E+04	2.54E+04
	Std	1.03E+00	1.39E+04	1.37E+04	1.37E+04	1.47E+04	1.42E+04	1.26E+04	1.49E+04
F18	Best	1.90E+03	1.91E+03	1.90E+03	1.91E+03	1.92E+03	1.91E+03	1.91E+03	1.90E+03
	Mean	1.90E+03	5.16E+03	1.96E+03	4.07E+03	8.63E+03	8.00E+03	1.30E+04	2.53E+03
	Std	5.99E-01	5.11E+03	1.17E+02	4.65E+03	6.86E+03	6.39E+03	1.01E+04	2.45E+03
F19	Best	2.00E+03	2.00E+03	2.00E+03	2.01E+03	2.02E+03	2.02E+03	2.00E+03	2.00E+03
	Mean	2.00E+03	2.03E+03	2.01E+03	2.07E+03	2.08E+03	2.04E+03	2.02E+03	2.04E+03
	Std	2.27E+00	1.25E+01	9.95E+00	4.27E+01	5.48E+01	3.57E+01	2.84E+01	4.04E+01
F20	Best	2.20E+03	2.20E+03	2.20E+03	2.20E+03	2.20E+03	2.20E+03	2.20E+03	2.20E+03
	Mean	2.29E+03	2.29E+03	2.22E+03	2.20E+03	2.29E+03	2.30E+03	2.30E+03	2.31E+03
	Std	4.37E+01	5.20E+01	4.60E+01	1.39E+00	6.04E+01	5.07E+01	4.97E+01	4.62E+01
F21	Best	2.22E+03	2.21E+03	2.21E+03	2.22E+03	2.20E+03	2.21E+03	2.20E+03	2.20E+03
	Mean	2.30E+03	2.29E+03	2.30E+03	2.30E+03	2.34E+03	2.30E+03	2.29E+03	2.29E+03
	Std	1.51E+01	2.75E+01	2.32E+01	1.58E+01	2.21E+02	4.73E+01	2.53E+01	2.34E+01
F22	Best	2.60E+03	2.61E+03	2.61E+03	2.61E+03	2.61E+03	2.61E+03	2.61E+03	2.61E+03
	Mean	2.61E+03	2.62E+03	2.62E+03	2.63E+03	2.62E+03	2.62E+03	2.62E+03	2.62E+03
	Std	3.01E+00	5.96E+00	5.83E+00	1.05E+01	8.03E+00	8.52E+00	6.95E+00	8.29E+00
F23	Best	2.50E+03	2.50E+03	2.50E+03	2.50E+03	2.50E+03	2.50E+03	2.50E+03	2.50E+03
	Mean	2.69E+03	2.75E+03	2.72E+03	2.65E+03	2.70E+03	2.75E+03	2.76E+03	2.75E+03
	Std	9.53E+01	3.75E+01	8.71E+01	1.24E+02	1.13E+02	5.10E+01	3.76E+01	5.27E+01
F24	Best	2.90E+03	2.90E+03	2.90E+03	2.90E+03	2.90E+03	2.90E+03	2.90E+03	2.90E+03
	Mean	2.92E+03	2.93E+03	2.92E+03	2.93E+03	2.94E+03	2.93E+03	2.93E+03	2.93E+03
	Std	2.28E+01	2.93E+01	2.61E+01	2.71E+01	3.16E+01	2.57E+01	3.29E+01	3.09E+01
F25	Best	2.60E+03	2.90E+03	2.90E+03	2.60E+03	2.80E+03	2.80E+03	2.90E+03	2.80E+03
	Mean	2.92E+03	3.12E+03	2.93E+03	2.95E+03	3.05E+03	3.13E+03	3.07E+03	3.05E+03
	Std	1.42E+02	3.74E+02	3.78E+01	1.08E+02	2.94E+02	3.92E+02	3.15E+02	2.56E+02
F26	Best	3.09E+03	3.09E+03	3.09E+03	3.09E+03	3.09E+03	3.09E+03	3.09E+03	3.09E+03
	Mean	3.09E+03	3.09E+03	3.09E+03	3.09E+03	3.09E+03	3.09E+03	3.09E+03	3.09E+03
	Std	1.38E+00	1.31E+00	2.12E+00	2.44E+00	2.39E+00	1.73E+00	1.18E+01	8.31E+00
F27	Best	3.10E+03	3.16E+03	3.10E+03	3.10E+03	3.17E+03	3.10E+03	3.10E+03	3.10E+03
	Mean	3.25E+03	3.32E+03	3.23E+03	3.24E+03	3.41E+03	3.30E+03	3.35E+03	3.38E+03
	Std	1.50E+02	1.44E+02	1.46E+02	1.27E+02	1.46E+02	1.32E+02	1.56E+02	1.48E+02
F28	Best	3.13E+03	3.13E+03	3.13E+03	3.14E+03	3.14E+03	3.14E+03	3.13E+03	3.13E+03
	Mean	3.13E+03	3.20E+03	3.16E+03	3.21E+03	3.23E+03	3.18E+03	3.19E+03	3.19E+03
	Std	5.32E+00	6.40E+01	2.51E+01	5.09E+01	7.13E+01	4.00E+01	5.72E+01	4.99E+01
F29	Best	3.42E+03	6.14E+03	3.94E+03	4.83E+03	4.34E+03	5.11E+03	5.34E+03	4.38E+03
	Mean	1.32E+05	1.71E+05	1.33E+05	2.44E+05	3.79E+05	2.59E+04	1.56E+05	2.44E+05
	Std	3.00E+05	3.28E+05	3.10E+05	3.97E+05	5.90E+05	1.14E+05	3.44E+05	4.36E+05

**Table A8:** Statistical results derived from EMSMA and SMA variants for CEC 2017 (Dim = 30).

Function	Index	EMSMA	SMA	DTSMA	ISMA	AOSMA	LSMA	ESMA	MSMA
F1	Best	1.09E+02	1.04E+03	1.03E+02	1.27E+02	2.07E+02	1.46E+02	2.22E+02	1.48E+02
	Mean	3.60E+03	8.18E+03	6.58E+03	5.08E+03	8.53E+03	6.39E+03	7.43E+03	1.23E+04
	Std	4.28E+03	6.79E+03	7.41E+03	5.94E+03	7.92E+03	5.94E+03	6.60E+03	2.49E+04
F2	Best	3.01E+02	3.81E+02	5.08E+02	9.51E+03	3.96E+03	6.65E+02	5.83E+02	2.65E+03
	Mean	3.05E+02	1.11E+03	4.74E+03	1.69E+04	1.71E+04	2.81E+03	5.33E+03	1.11E+04
	Std	3.07E+00	9.08E+02	4.20E+03	4.79E+03	7.86E+03	1.53E+03	3.89E+03	6.96E+03
F3	Best	4.00E+02	4.59E+02	4.24E+02	4.73E+02	4.46E+02	4.36E+02	4.64E+02	4.59E+02
	Mean	4.12E+02	4.98E+02	4.96E+02	5.11E+02	5.18E+02	4.98E+02	4.94E+02	4.94E+02
	Std	2.13E+01	1.87E+01	1.92E+01	2.45E+01	3.29E+01	1.78E+01	1.45E+01	1.80E+01
F4	Best	5.62E+02	5.56E+02	5.45E+02	6.21E+02	5.99E+02	5.47E+02	5.58E+02	5.67E+02
	Mean	6.14E+02	6.05E+02	5.93E+02	7.04E+02	6.71E+02	6.07E+02	6.06E+02	6.40E+02
	Std	2.64E+01	2.91E+01	2.38E+01	4.53E+01	4.11E+01	2.77E+01	2.69E+01	3.18E+01
F5	Best	6.00E+02	6.01E+02	6.01E+02	6.37E+02	6.18E+02	6.01E+02	6.00E+02	6.04E+02
	Mean	6.00E+02	6.05E+02	6.05E+02	6.53E+02	6.40E+02	6.16E+02	6.02E+02	6.14E+02
	Std	3.60E-03	3.48E+00	2.50E+00	7.86E+00	1.23E+01	1.14E+01	1.69E+00	6.90E+00
F6	Best	7.77E+02	8.05E+02	7.85E+02	8.65E+02	8.42E+02	7.88E+02	7.72E+02	8.08E+02
	Mean	8.64E+02	8.48E+02	8.38E+02	1.05E+03	1.00E+03	8.58E+02	8.37E+02	9.07E+02
	Std	3.78E+01	2.84E+01	3.04E+01	9.57E+01	9.33E+01	4.22E+01	3.02E+01	5.16E+01
F7	Best	8.59E+02	8.62E+02	8.55E+02	8.99E+02	8.82E+02	8.58E+02	8.44E+02	8.71E+02
	Mean	9.04E+02	9.08E+02	8.90E+02	9.57E+02	9.44E+02	9.12E+02	8.97E+02	9.26E+02
	Std	2.27E+01	2.83E+01	2.48E+01	2.54E+01	3.09E+01	3.10E+01	2.38E+01	2.96E+01
F8	Best	9.02E+02	1.26E+03	1.00E+03	2.79E+03	2.97E+03	1.02E+03	1.02E+03	1.90E+03
	Mean	1.05E+03	3.56E+03	2.20E+03	4.29E+03	5.01E+03	3.12E+03	2.74E+03	3.47E+03
	Std	1.53E+02	1.46E+03	8.99E+02	8.20E+02	1.14E+03	1.59E+03	1.51E+03	8.89E+02
F9	Best	2.30E+03	3.23E+03	3.50E+03	3.89E+03	3.27E+03	3.58E+03	2.96E+03	3.15E+03
	Mean	3.99E+03	4.59E+03	4.86E+03	5.20E+03	4.85E+03	4.97E+03	4.48E+03	4.53E+03
	Std	5.70E+02	6.33E+02	6.22E+02	6.36E+02	7.26E+02	7.51E+02	6.50E+02	6.58E+02
F10	Best	1.12E+03	1.15E+03	1.14E+03	1.16E+03	1.23E+03	1.20E+03	1.13E+03	1.14E+03
	Mean	1.16E+03	1.26E+03	1.25E+03	1.28E+03	1.39E+03	1.37E+03	1.21E+03	1.24E+03
	Std	3.07E+01	5.43E+01	5.45E+01	5.36E+01	8.76E+01	6.60E+01	4.11E+01	5.12E+01
F11	Best	7.96E+03	3.92E+05	5.09E+04	3.06E+05	5.45E+05	1.77E+05	2.37E+05	1.62E+05
	Mean	6.62E+04	2.28E+06	1.19E+06	7.52E+06	5.60E+06	5.57E+06	2.08E+06	1.95E+06
	Std	5.04E+04	1.68E+06	9.36E+05	6.22E+06	3.83E+06	4.08E+06	1.66E+06	1.28E+06
F12	Best	1.33E+03	7.65E+03	1.46E+03	1.26E+04	2.54E+04	4.26E+04	1.56E+03	1.92E+03
	Mean	1.45E+03	3.24E+04	2.06E+04	1.35E+05	2.22E+05	2.50E+05	2.71E+04	2.70E+04
	Std	1.30E+02	2.51E+04	2.26E+04	9.82E+04	1.44E+05	1.60E+05	2.42E+04	2.55E+04
F13	Best	1.42E+03	1.85E+03	2.82E+03	1.74E+03	1.06E+04	6.58E+03	4.67E+03	2.28E+03
	Mean	1.44E+03	7.55E+04	4.28E+04	3.73E+04	1.04E+05	6.02E+04	1.02E+05	9.13E+04
	Std	1.20E+01	4.72E+04	3.20E+04	3.60E+04	7.60E+04	4.33E+04	7.86E+04	7.47E+04
F14	Best	1.51E+03	1.87E+03	1.56E+03	6.98E+03	1.07E+04	4.36E+04	1.69E+03	1.68E+03
	Mean	1.54E+03	2.71E+04	2.14E+04	4.58E+04	6.18E+04	1.40E+05	2.24E+04	1.33E+04
	Std	2.47E+01	1.51E+04	1.44E+04	3.27E+04	5.33E+04	7.92E+04	1.40E+04	1.36E+04
F15	Best	1.61E+03	1.86E+03	1.78E+03	2.19E+03	2.13E+03	2.02E+03	1.98E+03	1.88E+03
	Mean	2.25E+03	2.56E+03	2.35E+03	3.06E+03	2.97E+03	2.67E+03	2.63E+03	2.66E+03
	Std	3.03E+02	2.94E+02	3.25E+02	3.88E+02	3.62E+02	3.45E+02	2.72E+02	3.66E+02
F16	Best	1.72E+03	1.92E+03	1.80E+03	1.96E+03	2.07E+03	1.97E+03	1.83E+03	1.91E+03
	Mean	1.83E+03	2.29E+03	2.15E+03	2.37E+03	2.58E+03	2.32E+03	2.37E+03	2.36E+03
	Std	8.18E+01	2.08E+02	2.03E+02	2.18E+02	2.52E+02	2.06E+02	2.54E+02	2.23E+02
F17	Best	1.83E+03	1.21E+05	3.31E+04	1.32E+05	2.21E+05	1.01E+05	7.61E+04	1.10E+05
	Mean	6.42E+03	1.08E+06	5.02E+05	9.43E+05	1.52E+06	8.58E+05	1.03E+06	1.41E+06
	Std	5.52E+03	9.27E+05	4.57E+05	9.77E+05	1.16E+06	9.82E+05	1.18E+06	1.21E+06
F18	Best	1.91E+03	2.08E+03	1.93E+03	6.26E+03	2.94E+03	9.55E+03	2.05E+03	2.00E+03
	Mean	1.92E+03	3.26E+04	2.46E+04	1.41E+05	2.49E+04	9.23E+04	2.60E+04	1.39E+04
	Std	7.12E+00	2.20E+04	2.00E+04	9.74E+04	2.24E+04	4.92E+04	2.22E+04	1.90E+04
F19	Best	2.03E+03	2.08E+03	2.06E+03	2.18E+03	2.32E+03	2.28E+03	2.10E+03	2.09E+03
	Mean	2.20E+03	2.52E+03	2.41E+03	2.53E+03	2.67E+03	2.62E+03	2.52E+03	2.41E+03
	Std	1.37E+02	1.86E+02	1.81E+02	1.49E+02	1.71E+02	1.53E+02	1.82E+02	1.81E+02
F20	Best	2.35E+03	2.35E+03	2.34E+03	2.22E+03	2.38E+03	2.35E+03	2.34E+03	2.37E+03
	Mean	2.40E+03	2.41E+03	2.39E+03	2.49E+03	2.46E+03	2.42E+03	2.41E+03	2.41E+03
	Std	2.36E+01	2.82E+01	2.86E+01	7.29E+01	3.58E+01	3.04E+01	2.56E+01	2.64E+01
F21	Best	2.30E+03	2.30E+03	2.30E+03	2.30E+03	2.30E+03	2.30E+03	2.30E+03	2.30E+03
	Mean	3.95E+03	5.78E+03	5.31E+03	2.30E+03	6.25E+03	6.06E+03	5.75E+03	5.25E+03
	Std	1.80E+03	8.18E+02	1.63E+03	2.04E+00	1.55E+03	1.18E+03	9.56E+02	1.67E+03
F22	Best	2.69E+03	2.71E+03	2.70E+03	2.78E+03	2.73E+03	2.70E+03	2.70E+03	2.70E+03
	Mean	2.76E+03	2.76E+03	2.74E+03	2.89E+03	2.83E+03	2.76E+03	2.76E+03	2.76E+03
	Std	3.27E+01	2.81E+01	2.16E+01	6.02E+01	4.50E+01	2.84E+01	2.35E+01	2.53E+01
F23	Best	2.86E+03	2.87E+03	2.87E+03	2.89E+03	2.89E+03	2.87E+03	2.88E+03	2.87E+03
	Mean	2.91E+03	2.93E+03	2.91E+03	3.01E+03	2.99E+03	2.92E+03	2.94E+03	2.93E+03
	Std	3.23E+01	3.12E+01	2.50E+01	4.71E+01	4.27E+01	2.83E+01	3.19E+01	2.94E+01
F24	Best	2.88E+03	2.88E+03	2.88E+03	2.89E+03	2.88E+03	2.88E+03	2.88E+03	2.88E+03
	Mean	2.89E+03	2.89E+03	2.89E+03	2.93E+03	2.90E+03	2.89E+03	2.89E+03	2.89E+03
	Std	9.62E+00	1.75E+01	7.44E+00	2.42E+01	1.63E+01	1.11E+01	1.44E+01	1.59E+01
F25	Best	2.80E+03	4.23E+03	2.90E+03	2.80E+03	4.65E+03	4.11E+03	4.16E+03	2.90E+03
	Mean	4.28E+03	4.82E+03	4.63E+03	5.15E+03	5.70E+03	4.86E+03	4.77E+03	4.82E+03
	Std	8.81E+02	2.95E+02	3.60E+02	1.84E+03	5.30E+02	3.36E+02	2.99E+02	4.18E+02
F26	Best	3.19E+03	3.20E+03	3.20E+03	3.22E+03	3.21E+03	3.18E+03	3.20E+03	3.21E+03
	Mean	3.22E+03	3.22E+03	3.22E+03	3.26E+03	3.24E+03	3.23E+03	3.22E+03	3.23E+03
	Std	1.33E+01	1.63E+01	1.19E+01	3.09E+01	2.02E+01	1.96E+01	1.57E+01	1.70E+01
F27	Best	3.10E+03	3.20E+03	3.21E+03	3.21E+03	3.21E+03	3.21E+03	3.20E+03	3.12E+03
	Mean	3.13E+03	3.25E+03	3.25E+03	3.26E+03	3.28E+03	3.26E+03	3.25E+03	3.25E+03
	Std	5.31E+01	4.23E+01	3.54E+01	2.98E+01	5.12E+01	4.30E+01	3.64E+01	4.17E+01
F28	Best	3.28E+03	3.45E+03	3.46E+03	3.73E+03	3.64E+03	3.60E+03	3.49E+03	3.48E+03
	Mean	3.47E+03	3.83E+03	3.77E+03	4.34E+03	4.36E+03	4.02E+03	3.89E+03	4.01E+03
	Std	1.19E+02	2.07E+02	1.61E+02	3.46E+02	3.66E+02	2.34E+02	2.19E+02	2.34E+02
F29	Best	5.03E+03	9.77E+03	5.28E+03	2.40E+05	4.75E+04	1.02E+05	7.47E+03	5.94E+03
	Mean	7.46E+03	2.75E+04	1.45E+04	1.95E+06	4.91E+05	7.72E+05	1.66E+04	1.61E+04
	Std	5.81E+03	1.13E+04	6.75E+03	1.22E+06	3.55E+05	6.67E+05	6.46E+03	5.45E+03

**Table A9:** Statistical results derived from EMSMA and SMA variants for CEC 2017 (Dim = 50).

Function	Index	EMSMA	SMA	DTSMA	ISMA	AOSMA	LSMA	ESMA	MSMA
F1	Best	1.08E+02	6.43E+04	2.26E+02	4.08E+03	1.46E+03	4.09E+02	3.46E+03	1.25E+02
	Mean	3.37E+03	1.65E+05	2.91E+06	5.03E+04	1.26E+05	9.78E+03	1.50E+04	7.82E+03
	Std	3.52E+03	5.83E+04	8.83E+06	2.99E+04	2.17E+05	1.03E+04	9.00E+03	7.99E+03
F2	Best	3.66E+02	1.20E+04	3.23E+04	3.78E+04	6.54E+04	3.62E+04	2.01E+04	2.10E+04
	Mean	7.04E+02	3.23E+04	7.50E+04	6.86E+04	1.07E+05	6.08E+04	5.38E+04	3.68E+04
	Std	4.08E+02	1.33E+04	2.01E+04	1.13E+04	2.93E+04	1.69E+04	2.42E+04	8.43E+03
F3	Best	4.00E+02	4.72E+02	4.44E+02	4.84E+02	4.86E+02	5.06E+02	4.31E+02	4.33E+02
	Mean	4.55E+02	5.89E+02	5.90E+02	6.19E+02	5.85E+02	6.03E+02	5.71E+02	5.72E+02
	Std	4.50E+01	3.83E+01	4.40E+01	5.58E+01	5.92E+01	4.42E+01	5.77E+01	5.56E+01
F4	Best	6.67E+02	6.76E+02	6.53E+02	7.07E+02	7.53E+02	6.61E+02	6.42E+02	6.76E+02
	Mean	7.42E+02	7.59E+02	7.22E+02	8.45E+02	8.40E+02	7.66E+02	7.36E+02	7.89E+02
	Std	3.96E+01	4.52E+01	4.47E+01	4.48E+01	5.22E+01	5.64E+01	5.02E+01	4.22E+01
F5	Best	6.00E+02	6.12E+02	6.08E+02	6.50E+02	6.43E+02	6.17E+02	6.03E+02	6.26E+02
	Mean	6.00E+02	6.33E+02	6.19E+02	6.64E+02	6.62E+02	6.56E+02	6.15E+02	6.44E+02
	Std	5.58E-03	1.27E+01	6.40E+00	5.59E+00	7.19E+00	1.50E+01	1.01E+01	8.49E+00
F6	Best	8.92E+02	9.59E+02	9.42E+02	1.20E+03	1.13E+03	9.39E+02	8.84E+02	1.00E+03
	Mean	1.01E+03	1.03E+03	1.03E+03	1.49E+03	1.41E+03	1.06E+03	1.01E+03	1.29E+03
	Std	5.16E+01	4.49E+01	5.04E+01	1.37E+02	1.40E+02	6.23E+01	4.55E+01	1.40E+02
F7	Best	9.50E+02	9.43E+02	9.42E+02	1.05E+03	9.87E+02	9.66E+02	9.05E+02	9.74E+02
	Mean	1.03E+03	1.04E+03	1.03E+03	1.17E+03	1.13E+03	1.06E+03	1.02E+03	1.06E+03
	Std	4.43E+01	4.82E+01	4.50E+01	4.99E+01	5.78E+01	5.02E+01	4.85E+01	4.35E+01
F8	Best	1.45E+03	4.96E+03	2.98E+03	6.55E+03	7.78E+03	5.63E+03	4.45E+03	5.81E+03
	Mean	3.71E+03	1.20E+04	9.30E+03	1.22E+04	1.45E+04	1.22E+04	1.17E+04	1.09E+04
	Std	1.67E+03	3.71E+03	4.63E+03	1.87E+03	2.90E+03	4.02E+03	3.77E+03	2.44E+03
F9	Best	4.45E+03	5.31E+03	5.96E+03	6.48E+03	6.24E+03	6.92E+03	5.58E+03	5.58E+03
	Mean	5.99E+03	7.08E+03	8.57E+03	8.05E+03	8.46E+03	8.18E+03	7.51E+03	7.32E+03
	Std	7.57E+02	8.11E+02	1.25E+03	8.59E+02	1.05E+03	8.43E+02	9.35E+02	8.62E+02
F10	Best	1.17E+03	1.23E+03	1.29E+03	1.40E+03	1.35E+03	1.35E+03	1.22E+03	1.20E+03
	Mean	1.21E+03	1.40E+03	1.47E+03	1.56E+03	1.59E+03	1.56E+03	1.35E+03	1.37E+03
	Std	2.97E+01	8.34E+01	8.45E+01	1.12E+02	1.31E+02	9.14E+01	7.54E+01	7.32E+01
F11	Best	1.32E+05	2.17E+06	1.60E+06	1.01E+07	2.94E+06	9.52E+06	1.13E+06	9.46E+05
	Mean	8.01E+05	1.73E+07	8.80E+06	6.42E+07	4.15E+07	4.89E+07	1.49E+07	9.31E+06
	Std	5.51E+05	8.46E+06	4.67E+06	3.22E+07	2.48E+07	2.96E+07	7.58E+06	5.19E+06
F12	Best	1.45E+03	4.28E+04	2.48E+03	3.58E+04	9.54E+04	7.46E+04	5.70E+03	3.80E+03
	Mean	5.50E+03	7.08E+04	2.34E+04	1.54E+05	3.17E+05	3.36E+05	2.82E+04	2.56E+04
	Std	4.82E+03	1.78E+04	1.25E+04	9.58E+04	1.72E+05	2.38E+05	1.16E+04	1.27E+04
F13	Best	1.46E+03	9.21E+04	4.42E+04	4.63E+04	3.20E+04	3.80E+04	5.98E+04	6.92E+04
	Mean	1.52E+03	4.00E+05	1.85E+05	3.55E+05	4.29E+05	3.11E+05	4.39E+05	4.75E+05
	Std	3.11E+01	2.12E+05	1.20E+05	2.37E+05	2.86E+05	1.93E+05	2.57E+05	2.66E+05
F14	Best	1.54E+03	4.62E+03	1.93E+03	1.39E+04	4.03E+04	5.41E+04	2.01E+03	2.87E+03
	Mean	1.63E+03	2.71E+04	1.97E+04	5.74E+04	1.48E+05	1.84E+05	2.09E+04	2.17E+04
	Std	1.07E+02	1.12E+04	1.05E+04	3.51E+04	9.18E+04	9.42E+04	9.63E+03	1.00E+04
F15	Best	2.08E+03	2.58E+03	2.36E+03	3.11E+03	3.02E+03	2.83E+03	2.73E+03	2.45E+03
	Mean	2.99E+03	3.40E+03	3.19E+03	4.10E+03	4.07E+03	3.65E+03	3.50E+03	3.48E+03
	Std	4.76E+02	3.44E+02	5.08E+02	4.10E+02	5.59E+02	3.30E+02	4.45E+02	3.91E+02
F16	Best	2.03E+03	2.41E+03	2.37E+03	2.73E+03	3.00E+03	2.76E+03	2.30E+03	2.69E+03
	Mean	2.60E+03	3.31E+03	3.01E+03	3.57E+03	3.88E+03	3.66E+03	3.20E+03	3.49E+03
	Std	3.09E+02	3.30E+02	2.54E+02	4.22E+02	4.34E+02	3.93E+02	4.04E+02	3.52E+02
F17	Best	6.42E+03	3.30E+05	2.79E+05	3.13E+05	4.05E+05	5.59E+05	5.14E+05	4.05E+05
	Mean	3.14E+04	3.24E+06	2.26E+06	2.49E+06	4.10E+06	3.48E+06	2.65E+06	2.52E+06
	Std	1.87E+04	2.11E+06	1.38E+06	2.00E+06	2.87E+06	2.23E+06	1.66E+06	1.81E+06
F18	Best	1.92E+03	2.14E+03	2.15E+03	2.54E+05	4.06E+04	1.29E+05	2.02E+03	2.05E+03
	Mean	1.95E+03	1.61E+04	1.94E+04	8.11E+05	1.50E+05	6.23E+05	1.55E+04	2.57E+04
	Std	2.36E+01	1.74E+04	1.69E+04	3.16E+05	1.13E+05	3.89E+05	1.66E+04	1.75E+04
F19	Best	2.25E+03	2.58E+03	2.44E+03	2.60E+03	2.71E+03	2.80E+03	2.51E+03	2.57E+03
	Mean	2.77E+03	3.25E+03	3.11E+03	3.25E+03	3.49E+03	3.34E+03	3.13E+03	3.12E+03
	Std	2.48E+02	2.96E+02	3.65E+02	3.10E+02	3.32E+02	2.95E+02	3.10E+02	2.49E+02
F20	Best	2.45E+03	2.46E+03	2.42E+03	2.50E+03	2.54E+03	2.45E+03	2.44E+03	2.43E+03
	Mean	2.52E+03	2.53E+03	2.50E+03	2.71E+03	2.67E+03	2.53E+03	2.52E+03	2.54E+03
	Std	3.79E+01	4.69E+01	4.82E+01	7.74E+01	6.91E+01	3.75E+01	4.43E+01	4.97E+01
F21	Best	6.46E+03	7.18E+03	7.41E+03	2.32E+03	7.47E+03	7.71E+03	7.05E+03	7.35E+03
	Mean	8.34E+03	9.05E+03	9.85E+03	1.03E+04	1.00E+04	9.79E+03	9.08E+03	9.22E+03
	Std	7.90E+02	9.69E+02	1.07E+03	2.22E+03	8.97E+02	1.27E+03	8.76E+02	7.49E+02
F22	Best	2.90E+03	2.87E+03	2.86E+03	3.06E+03	2.98E+03	2.84E+03	2.85E+03	2.91E+03
	Mean	3.00E+03	2.97E+03	2.93E+03	3.25E+03	3.15E+03	2.98E+03	2.96E+03	3.01E+03
	Std	4.69E+01	4.62E+01	3.71E+01	1.19E+02	7.35E+01	5.73E+01	5.25E+01	6.25E+01
F23	Best	2.99E+03	3.06E+03	3.01E+03	3.16E+03	3.09E+03	3.02E+03	3.02E+03	3.03E+03
	Mean	3.13E+03	3.15E+03	3.08E+03	3.34E+03	3.25E+03	3.12E+03	3.15E+03	3.16E+03
	Std	8.68E+01	5.73E+01	3.72E+01	9.56E+01	7.89E+01	4.88E+01	4.69E+01	5.15E+01
F24	Best	2.93E+03	2.96E+03	2.98E+03	3.03E+03	3.03E+03	3.00E+03	2.96E+03	2.98E+03
	Mean	3.03E+03	3.06E+03	3.07E+03	3.12E+03	3.12E+03	3.07E+03	3.05E+03	3.08E+03
	Std	4.08E+01	3.26E+01	3.81E+01	3.38E+01	3.23E+01	3.12E+01	2.79E+01	4.67E+01
F25	Best	2.90E+03	2.90E+03	5.11E+03	2.92E+03	2.91E+03	2.90E+03	2.90E+03	2.90E+03
	Mean	5.79E+03	5.68E+03	5.96E+03	6.89E+03	7.11E+03	5.72E+03	5.66E+03	6.37E+03
	Std	1.95E+03	1.76E+03	4.52E+02	3.68E+03	2.67E+03	1.74E+03	1.35E+03	2.18E+03
F26	Best	3.25E+03	3.30E+03	3.26E+03	3.43E+03	3.33E+03	3.31E+03	3.28E+03	3.34E+03
	Mean	3.40E+03	3.44E+03	3.43E+03	3.66E+03	3.59E+03	3.45E+03	3.43E+03	3.55E+03
	Std	7.71E+01	8.23E+01	8.81E+01	1.14E+02	1.22E+02	7.73E+01	9.10E+01	1.07E+02
F27	Best	3.26E+03	3.28E+03	3.32E+03	3.29E+03	3.32E+03	3.27E+03	3.26E+03	3.30E+03
	Mean	3.29E+03	3.35E+03	3.54E+03	3.39E+03	3.61E+03	3.54E+03	3.32E+03	3.35E+03
	Std	3.41E+01	3.55E+01	4.28E+02	6.01E+01	9.65E+02	9.64E+02	2.34E+01	3.76E+01
F28	Best	3.34E+03	3.94E+03	3.72E+03	5.10E+03	4.58E+03	4.24E+03	3.97E+03	4.20E+03
	Mean	3.82E+03	4.69E+03	4.32E+03	6.03E+03	5.64E+03	5.10E+03	4.51E+03	4.74E+03
	Std	2.67E+02	3.38E+02	3.28E+02	4.29E+02	5.16E+02	4.32E+02	3.01E+02	3.87E+02
F29	Best	5.82E+05	1.73E+06	7.60E+05	2.50E+07	9.45E+06	1.35E+07	7.23E+05	6.72E+05
	Mean	6.25E+05	3.05E+06	2.10E+06	5.37E+07	2.23E+07	2.78E+07	1.77E+06	1.72E+06
	Std	5.84E+04	6.71E+05	8.83E+05	1.25E+07	7.12E+06	7.72E+06	4.84E+05	4.84E+05

**Table A10:** Statistical results derived from EMSMA and SMA variants for CEC 2017 (Dim = 100).

Function	Index	EMSMA	SMA	DTSMA	ISMA	AOSMA	LSMA	ESMA	MSMA
F1	Best	1.51E+03	9.78E+06	5.02E+08	1.43E+07	1.06E+07	9.29E+05	1.73E+06	1.89E+05
	Mean	7.32E+03	1.86E+07	4.24E+09	3.98E+07	6.83E+07	2.74E+06	3.03E+06	1.24E+08
	Std	5.98E+03	4.56E+06	2.17E+09	1.69E+07	5.54E+07	1.70E+06	7.64E+05	1.64E+08
F2	Best	2.75E+04	1.87E+05	2.87E+05	2.36E+05	2.72E+05	2.32E+05	1.59E+05	1.61E+05
	Mean	4.54E+04	4.03E+05	4.73E+05	2.67E+05	3.34E+05	4.15E+05	4.07E+05	2.19E+05
	Std	9.35E+03	1.39E+05	8.58E+04	1.80E+04	2.97E+04	1.38E+05	1.89E+05	2.19E+04
F3	Best	4.73E+02	6.73E+02	7.61E+02	7.80E+02	7.96E+02	6.49E+02	6.32E+02	7.19E+02
	Mean	5.55E+02	7.72E+02	9.60E+02	9.48E+02	1.01E+03	8.29E+02	7.30E+02	9.04E+02
	Std	5.89E+01	6.34E+01	1.08E+02	7.83E+01	1.11E+02	7.69E+01	4.67E+01	1.03E+02
F4	Best	9.63E+02	1.01E+03	9.46E+02	1.26E+03	1.15E+03	1.02E+03	9.87E+02	1.04E+03
	Mean	1.12E+03	1.21E+03	1.17E+03	1.35E+03	1.36E+03	1.22E+03	1.18E+03	1.26E+03
	Std	8.96E+01	8.82E+01	9.10E+01	5.43E+01	8.67E+01	1.07E+02	8.48E+01	8.99E+01
F5	Best	6.00E+02	6.32E+02	6.31E+02	6.62E+02	6.60E+02	6.42E+02	6.23E+02	6.41E+02
	Mean	6.00E+02	6.49E+02	6.49E+02	6.72E+02	6.70E+02	6.66E+02	6.37E+02	6.58E+02
	Std	4.38E-03	8.37E+00	8.01E+00	3.66E+00	4.83E+00	6.77E+00	6.70E+00	5.27E+00
F6	Best	1.25E+03	1.63E+03	1.66E+03	2.46E+03	2.45E+03	1.61E+03	1.47E+03	2.13E+03
	Mean	1.59E+03	1.91E+03	1.92E+03	3.05E+03	2.91E+03	1.96E+03	1.74E+03	2.43E+03
	Std	1.53E+02	1.63E+02	1.81E+02	1.85E+02	2.28E+02	1.85E+02	1.36E+02	1.72E+02
F7	Best	1.28E+03	1.29E+03	1.23E+03	1.61E+03	1.52E+03	1.27E+03	1.30E+03	1.39E+03
	Mean	1.44E+03	1.52E+03	1.46E+03	1.78E+03	1.76E+03	1.54E+03	1.44E+03	1.59E+03
	Std	9.06E+01	1.20E+02	1.05E+02	7.28E+01	9.46E+01	1.31E+02	9.25E+01	1.06E+02
F8	Best	1.23E+04	1.94E+04	1.98E+04	2.35E+04	2.40E+04	1.94E+04	1.86E+04	1.94E+04
	Mean	1.81E+04	3.00E+04	3.66E+04	2.78E+04	3.00E+04	3.04E+04	2.77E+04	2.39E+04
	Std	3.08E+03	4.33E+03	1.12E+04	2.30E+03	3.93E+03	4.93E+03	4.31E+03	1.97E+03
F9	Best	1.02E+04	1.28E+04	1.50E+04	1.42E+04	1.56E+04	1.37E+04	1.16E+04	1.25E+04
	Mean	1.29E+04	1.61E+04	1.96E+04	1.73E+04	1.81E+04	1.81E+04	1.55E+04	1.57E+04
	Std	1.11E+03	1.47E+03	1.79E+03	1.63E+03	1.65E+03	1.69E+03	1.52E+03	1.39E+03
F10	Best	1.29E+03	2.30E+03	3.84E+03	6.27E+03	5.42E+03	3.43E+03	2.72E+03	3.51E+03
	Mean	1.49E+03	3.23E+03	7.34E+03	1.17E+04	1.74E+04	6.17E+03	7.24E+03	1.12E+04
	Std	9.14E+01	7.22E+02	2.95E+03	3.61E+03	6.15E+03	1.91E+03	3.52E+03	5.43E+03
F11	Best	8.56E+05	3.52E+07	2.03E+07	1.82E+08	1.05E+08	1.50E+08	2.51E+07	1.30E+07
	Mean	1.97E+06	1.35E+08	2.26E+08	4.67E+08	3.14E+08	3.89E+08	7.45E+07	5.23E+07
	Std	8.77E+05	5.93E+07	1.93E+08	1.77E+08	1.43E+08	1.55E+08	3.18E+07	2.25E+07
F12	Best	1.67E+03	4.78E+04	1.21E+04	1.99E+04	2.96E+04	7.25E+04	1.95E+04	1.05E+04
	Mean	5.15E+03	4.91E+05	3.30E+05	7.79E+04	2.60E+05	3.31E+05	1.37E+05	3.43E+04
	Std	4.31E+03	8.28E+05	1.02E+06	2.93E+04	4.16E+05	3.61E+05	1.69E+05	2.40E+04
F13	Best	3.39E+03	4.03E+05	8.18E+05	9.02E+05	6.06E+05	2.96E+05	5.58E+05	3.53E+05
	Mean	4.03E+04	2.60E+06	2.57E+06	2.14E+06	2.75E+06	2.49E+06	2.36E+06	1.79E+06
	Std	3.50E+04	1.44E+06	1.36E+06	1.11E+06	1.72E+06	1.25E+06	1.46E+06	1.18E+06
F14	Best	1.62E+03	2.45E+04	2.74E+03	2.37E+04	3.27E+04	5.96E+04	3.60E+03	4.26E+03
	Mean	4.04E+03	1.70E+05	4.70E+04	6.43E+04	1.78E+05	1.78E+05	2.69E+04	1.84E+04
	Std	3.44E+03	1.86E+05	6.59E+04	3.15E+04	1.39E+05	1.00E+05	3.23E+04	1.35E+04
F15	Best	3.75E+03	4.98E+03	3.62E+03	5.65E+03	5.80E+03	5.03E+03	4.03E+03	3.75E+03
	Mean	5.84E+03	5.99E+03	5.56E+03	7.69E+03	7.46E+03	6.75E+03	5.98E+03	5.94E+03
	Std	8.07E+02	5.41E+02	7.97E+02	8.71E+02	8.40E+02	7.20E+02	7.38E+02	8.19E+02
F16	Best	3.53E+03	4.66E+03	4.36E+03	4.19E+03	5.68E+03	4.99E+03	4.08E+03	4.20E+03
	Mean	4.78E+03	5.67E+03	5.49E+03	6.09E+03	7.01E+03	6.19E+03	5.38E+03	6.11E+03
	Std	6.67E+02	5.50E+02	5.26E+02	8.31E+02	6.52E+02	6.41E+02	5.78E+02	6.71E+02
F17	Best	4.63E+04	2.09E+06	1.28E+06	8.97E+05	1.55E+06	1.56E+06	1.18E+06	1.18E+06
	Mean	1.32E+05	5.76E+06	4.59E+06	3.05E+06	5.44E+06	5.91E+06	5.52E+06	4.18E+06
	Std	5.26E+04	2.74E+06	2.50E+06	1.48E+06	2.69E+06	3.33E+06	2.71E+06	2.20E+06
F18	Best	1.98E+03	9.42E+03	2.68E+03	4.02E+05	4.61E+05	1.35E+06	2.30E+03	2.87E+03
	Mean	4.24E+03	7.10E+04	1.56E+04	6.10E+06	2.13E+06	5.27E+06	2.14E+04	1.63E+04
	Std	3.19E+03	6.04E+04	1.08E+04	2.98E+06	1.01E+06	1.78E+06	1.94E+04	1.38E+04
F19	Best	3.93E+03	4.53E+03	4.14E+03	4.36E+03	4.08E+03	4.39E+03	4.06E+03	4.06E+03
	Mean	5.05E+03	5.36E+03	5.38E+03	5.43E+03	5.86E+03	5.77E+03	5.18E+03	5.20E+03
	Std	5.74E+02	4.59E+02	5.97E+02	5.02E+02	6.14E+02	6.01E+02	5.76E+02	4.81E+02
F20	Best	2.75E+03	2.81E+03	2.78E+03	3.12E+03	3.05E+03	2.86E+03	2.80E+03	2.83E+03
	Mean	2.89E+03	3.01E+03	2.95E+03	3.53E+03	3.43E+03	3.04E+03	2.98E+03	3.03E+03
	Std	7.39E+01	9.17E+01	9.94E+01	2.05E+02	1.57E+02	1.04E+02	8.42E+01	1.18E+02
F21	Best	1.32E+04	1.50E+04	1.79E+04	1.74E+04	1.48E+04	1.63E+04	1.39E+04	1.57E+04
	Mean	1.60E+04	1.86E+04	2.24E+04	2.16E+04	2.02E+04	2.02E+04	1.75E+04	1.83E+04
	Std	1.31E+03	1.44E+03	2.12E+03	1.81E+03	2.00E+03	1.58E+03	1.28E+03	1.32E+03
F22	Best	3.05E+03	3.20E+03	3.25E+03	3.68E+03	3.50E+03	3.33E+03	3.15E+03	3.19E+03
	Mean	3.18E+03	3.36E+03	3.36E+03	4.06E+03	3.79E+03	3.48E+03	3.28E+03	3.36E+03
	Std	5.73E+01	7.62E+01	5.14E+01	1.74E+02	1.30E+02	8.37E+01	6.43E+01	7.03E+01
F23	Best	3.67E+03	3.78E+03	3.67E+03	4.41E+03	3.91E+03	3.77E+03	3.66E+03	3.89E+03
	Mean	3.85E+03	3.97E+03	3.85E+03	4.80E+03	4.40E+03	4.00E+03	3.89E+03	4.09E+03
	Std	8.22E+01	1.09E+02	7.37E+01	2.51E+02	2.07E+02	1.23E+02	9.59E+01	1.32E+02
F24	Best	3.13E+03	3.35E+03	3.60E+03	3.57E+03	3.54E+03	3.38E+03	3.22E+03	3.35E+03
	Mean	3.26E+03	3.45E+03	3.97E+03	3.68E+03	3.69E+03	3.54E+03	3.39E+03	3.59E+03
	Std	6.02E+01	5.35E+01	2.56E+02	6.64E+01	7.28E+01	7.32E+01	6.39E+01	8.00E+01
F25	Best	2.90E+03	3.13E+03	1.03E+04	5.55E+03	7.52E+03	1.12E+04	1.06E+04	1.19E+04
	Mean	1.37E+04	1.31E+04	1.19E+04	1.99E+04	1.89E+04	1.34E+04	1.24E+04	1.58E+04
	Std	3.97E+03	1.76E+03	9.59E+02	7.96E+03	3.62E+03	1.05E+03	7.77E+02	1.87E+03
F26	Best	3.41E+03	3.45E+03	3.42E+03	3.61E+03	3.63E+03	3.44E+03	3.43E+03	3.50E+03
	Mean	3.54E+03	3.62E+03	3.55E+03	3.97E+03	3.82E+03	3.61E+03	3.52E+03	3.66E+03
	Std	8.50E+01	9.14E+01	6.14E+01	2.45E+02	1.28E+02	8.48E+01	5.67E+01	1.05E+02
F27	Best	3.30E+03	3.46E+03	3.73E+03	3.56E+03	3.62E+03	3.47E+03	3.41E+03	3.47E+03
	Mean	3.36E+03	3.53E+03	6.87E+03	3.71E+03	3.76E+03	3.61E+03	3.50E+03	3.60E+03
	Std	2.81E+01	3.99E+01	3.43E+03	5.63E+01	6.92E+01	5.32E+01	3.61E+01	5.34E+01
F28	Best	5.68E+03	5.95E+03	5.95E+03	9.02E+03	7.62E+03	7.42E+03	5.79E+03	6.09E+03
	Mean	6.85E+03	7.33E+03	7.25E+03	1.04E+04	9.43E+03	8.86E+03	6.89E+03	7.52E+03
	Std	5.23E+02	5.68E+02	7.61E+02	9.65E+02	1.13E+03	7.55E+02	5.57E+02	6.27E+02
F29	Best	6.23E+03	4.15E+05	3.19E+04	3.22E+07	4.95E+06	1.68E+07	5.95E+04	5.22E+04
	Mean	1.30E+04	1.58E+06	4.65E+05	1.00E+08	4.01E+07	6.34E+07	1.78E+05	2.52E+05
	Std	5.24E+03	9.09E+05	4.98E+05	4.14E+07	2.16E+07	3.15E+07	1.10E+05	1.97E+05

**Table A11:** Statistical results derived from EMSMA and SMA variants for CEC 2022 (Dim = 10).

Function	Index	EMSMA	SMA	DTSMA	ISMA	AOSMA	LSMA	ESMA	MSMA
F1	Best	3.00E+02	3.00E+02	3.00E+02	3.00E+02	3.00E+02	3.00E+02	3.00E+02	3.00E+02
	Mean	3.00E+02	3.00E+02	3.00E+02	3.00E+02	3.00E+02	3.00E+02	3.00E+02	3.00E+02
	Std	4.09E-07	1.14E-03	1.70E-05	2.04E-04	5.46E-03	3.20E-04	2.11E-04	1.18E-04
F2	Best	4.00E+02	4.01E+02	4.00E+02	4.00E+02	4.00E+02	4.00E+02	4.00E+02	4.00E+02
	Mean	4.07E+02	4.12E+02	4.09E+02	4.17E+02	4.14E+02	4.09E+02	4.10E+02	4.12E+02
	Std	9.70E+00	1.66E+01	9.35E+00	2.45E+01	2.12E+01	1.11E+01	1.35E+01	1.65E+01
F3	Best	6.00E+02	6.00E+02	6.00E+02	6.00E+02	6.00E+02	6.00E+02	6.00E+02	6.00E+02
	Mean	6.00E+02	6.00E+02	6.00E+02	6.05E+02	6.03E+02	6.00E+02	6.00E+02	6.00E+02
	Std	2.82E-03	3.76E-02	5.31E-02	4.22E+00	4.56E+00	1.78E-01	2.44E-02	8.88E-01
F4	Best	8.03E+02	8.09E+02	8.05E+02	8.06E+02	8.05E+02	8.06E+02	8.09E+02	8.05E+02
	Mean	8.09E+02	8.25E+02	8.16E+02	8.24E+02	8.28E+02	8.20E+02	8.24E+02	8.17E+02
	Std	3.86E+00	9.75E+00	6.31E+00	9.14E+00	9.48E+00	8.58E+00	9.17E+00	7.17E+00
F5	Best	9.00E+02	9.00E+02	9.00E+02	9.00E+02	9.01E+02	9.00E+02	9.00E+02	9.00E+02
	Mean	9.00E+02	9.01E+02	9.00E+02	9.42E+02	1.04E+03	9.01E+02	9.05E+02	9.35E+02
	Std	1.36E-01	1.99E+00	5.54E-01	7.46E+01	1.53E+02	1.52E+00	9.55E+00	5.38E+01
F6	Best	1.80E+03	1.92E+03	2.18E+03	1.88E+03	1.97E+03	2.00E+03	1.90E+03	1.84E+03
	Mean	1.80E+03	5.50E+03	5.63E+03	4.50E+03	4.95E+03	5.19E+03	5.15E+03	4.49E+03
	Std	1.24E+01	2.35E+03	1.79E+03	2.35E+03	2.17E+03	2.07E+03	1.91E+03	2.06E+03
F7	Best	2.00E+03	2.02E+03	2.00E+03	2.02E+03	2.00E+03	2.02E+03	2.00E+03	2.00E+03
	Mean	2.00E+03	2.02E+03	2.02E+03	2.03E+03	2.03E+03	2.02E+03	2.02E+03	2.02E+03
	Std	8.16E+00	7.79E-01	5.01E+00	7.78E+00	7.61E+00	2.87E+00	4.07E+00	4.83E+00
F8	Best	2.20E+03	2.20E+03	2.20E+03	2.20E+03	2.20E+03	2.20E+03	2.20E+03	2.20E+03
	Mean	2.21E+03	2.22E+03	2.22E+03	2.23E+03	2.22E+03	2.22E+03	2.22E+03	2.22E+03
	Std	8.79E+00	2.79E+00	5.02E+00	4.70E+00	5.45E+00	3.98E+00	2.84E+00	2.82E+00
F9	Best	2.53E+03	2.53E+03	2.53E+03	2.53E+03	2.53E+03	2.53E+03	2.53E+03	2.53E+03
	Mean	2.53E+03	2.53E+03	2.53E+03	2.53E+03	2.53E+03	2.53E+03	2.53E+03	2.53E+03
	Std	1.71E-11	3.28E-04	7.65E-10	3.29E-11	5.59E-05	2.84E-04	6.80E-05	1.05E-04
F10	Best	2.50E+03	2.50E+03	2.50E+03	2.50E+03	2.50E+03	2.50E+03	2.50E+03	2.41E+03
	Mean	2.52E+03	2.52E+03	2.50E+03	2.50E+03	2.53E+03	2.51E+03	2.54E+03	2.54E+03
	Std	4.23E+01	4.24E+01	1.57E+01	1.45E-01	5.67E+01	2.91E+01	5.49E+01	6.12E+01
F11	Best	2.60E+03	2.60E+03	2.60E+03	2.60E+03	2.60E+03	2.60E+03	2.60E+03	2.60E+03
	Mean	2.79E+03	2.79E+03	2.66E+03	2.66E+03	2.75E+03	2.72E+03	2.79E+03	2.73E+03
	Std	1.46E+02	1.84E+02	1.35E+02	1.16E+02	1.56E+02	1.63E+02	1.72E+02	1.46E+02
F12	Best	2.86E+03	2.86E+03	2.86E+03	2.86E+03	2.86E+03	2.86E+03	2.86E+03	2.86E+03
	Mean	2.86E+03	2.86E+03	2.86E+03	2.86E+03	2.86E+03	2.86E+03	2.86E+03	2.86E+03
	Std	1.69E+00	1.50E+00	1.48E+00	1.53E+00	1.66E+00	1.64E+00	1.20E+00	1.19E+00

**Table A12:** Statistical results derived from EMSMA and SMA variants for CEC 2022 (Dim = 20).

Function	Index	EMSMA	SMA	DTSMA	ISMA	AOSMA	LSMA	ESMA	MSMA
F1	Best	3.00E+02	3.00E+02	3.00E+02	3.00E+02	3.04E+02	3.00E+02	3.00E+02	3.00E+02
	Mean	3.00E+02	3.00E+02	3.03E+02	3.18E+02	4.47E+02	3.00E+02	3.07E+02	3.13E+02
	Std	6.50E-01	2.71E-01	6.62E+00	3.42E+01	1.81E+02	3.86E-01	3.43E+01	5.66E+01
F2	Best	4.00E+02	4.05E+02	4.04E+02	4.05E+02	4.09E+02	4.06E+02	4.04E+02	4.07E+02
	Mean	4.36E+02	4.48E+02	4.50E+02	4.56E+02	4.62E+02	4.50E+02	4.51E+02	4.56E+02
	Std	2.19E+01	1.60E+01	1.21E+01	1.91E+01	3.17E+01	1.97E+01	2.50E+01	2.81E+01
F3	Best	6.00E+02	6.00E+02	6.00E+02	6.06E+02	6.04E+02	6.01E+02	6.00E+02	6.00E+02
	Mean	6.00E+02	6.01E+02	6.01E+02	6.38E+02	6.24E+02	6.04E+02	6.00E+02	6.03E+02
	Std	3.99E-03	1.14E+00	4.50E-01	1.28E+01	1.14E+01	2.47E+00	2.07E-01	2.49E+00
F4	Best	8.16E+02	8.21E+02	8.27E+02	8.51E+02	8.34E+02	8.37E+02	8.31E+02	8.34E+02
	Mean	8.48E+02	8.67E+02	8.50E+02	8.76E+02	8.87E+02	8.66E+02	8.64E+02	8.65E+02
	Std	1.87E+01	2.38E+01	1.36E+01	1.54E+01	2.46E+01	1.77E+01	1.92E+01	1.69E+01
F5	Best	9.00E+02	9.11E+02	9.06E+02	1.26E+03	1.22E+03	9.19E+02	9.10E+02	1.00E+03
	Mean	9.04E+02	1.53E+03	1.11E+03	1.94E+03	2.07E+03	1.23E+03	1.37E+03	1.57E+03
	Std	4.16E+00	6.49E+02	2.68E+02	3.37E+02	3.34E+02	4.36E+02	4.49E+02	3.08E+02
F6	Best	1.81E+03	2.02E+03	1.96E+03	2.07E+03	2.01E+03	2.05E+03	1.97E+03	1.91E+03
	Mean	1.90E+03	2.02E+04	1.71E+04	6.02E+03	1.36E+04	2.06E+04	1.82E+04	1.34E+04
	Std	1.32E+02	6.72E+03	8.55E+03	5.70E+03	9.42E+03	7.26E+03	8.41E+03	9.67E+03
F7	Best	2.01E+03	2.03E+03	2.02E+03	2.06E+03	2.05E+03	2.03E+03	2.02E+03	2.02E+03
	Mean	2.03E+03	2.07E+03	2.05E+03	2.11E+03	2.13E+03	2.08E+03	2.09E+03	2.07E+03
	Std	1.85E+01	4.02E+01	2.42E+01	2.95E+01	4.69E+01	4.38E+01	5.50E+01	2.94E+01
F8	Best	2.21E+03	2.22E+03	2.22E+03	2.23E+03	2.22E+03	2.22E+03	2.22E+03	2.22E+03
	Mean	2.22E+03	2.25E+03	2.23E+03	2.26E+03	2.28E+03	2.25E+03	2.24E+03	2.23E+03
	Std	1.86E+00	4.95E+01	8.57E+00	4.63E+01	6.04E+01	3.48E+01	3.67E+01	1.76E+01
F9	Best	2.48E+03	2.48E+03	2.48E+03	2.48E+03	2.48E+03	2.48E+03	2.48E+03	2.48E+03
	Mean	2.48E+03	2.48E+03	2.48E+03	2.48E+03	2.48E+03	2.48E+03	2.48E+03	2.48E+03
	Std	9.87E-04	1.06E-01	3.27E-02	1.02E-01	1.82E-01	1.24E-01	9.74E-02	1.55E-01
F10	Best	2.40E+03	2.50E+03	2.50E+03	2.50E+03	2.50E+03	2.50E+03	2.43E+03	2.42E+03
	Mean	2.47E+03	2.88E+03	2.60E+03	2.52E+03	3.93E+03	3.71E+03	2.76E+03	2.74E+03
	Std	5.87E+01	2.79E+02	3.40E+02	5.11E+01	8.09E+02	8.00E+02	2.19E+02	2.19E+02
F11	Best	2.90E+03	2.60E+03	2.60E+03	2.60E+03	2.60E+03	2.60E+03	2.60E+03	2.60E+03
	Mean	2.90E+03	2.95E+03	2.91E+03	2.95E+03	2.91E+03	2.93E+03	2.93E+03	2.91E+03
	Std	5.39E+00	1.46E+02	8.97E+01	9.86E+01	1.02E+02	1.06E+02	8.25E+01	1.01E+02
F12	Best	2.93E+03	2.93E+03	2.94E+03	2.94E+03	2.94E+03	2.93E+03	2.94E+03	2.94E+03
	Mean	2.95E+03	2.95E+03	2.95E+03	2.96E+03	2.96E+03	2.94E+03	2.95E+03	2.95E+03
	Std	1.11E+01	1.01E+01	8.31E+00	1.63E+01	1.64E+01	5.05E+00	8.94E+00	8.87E+00
